# Supplementary material for: Economic impact of cholera in households in rural southern Malawi: a prospective study
Source: BMJ Open. 2022 Jun 1;12(6):e052337. doi: 10.1136/bmjopen-2021-052337 (PMC9161053; doi:10.1136/bmjopen-2021-052337)
Supplement: Supplementary data [file bmjopen-2021-052337supp001.pdf]

Cholera Surveillance In Malawi (CSIMA) Program

NSANJE DISTRICT USE ONLY

Form S.I.#:__________________ **Study label**

(To be filled by Data Management) **N**☐☐-☐☐☐☐

D-1: Cost-of-Illness Survey
First Interview (Day 0,1,2,3, 4, 5, 6, or 7)
Case Group: ☐Clinical Case ☐RDT Case ☐Laboratory Case

Place of interview: ☐Health facility / ☐Home / ☐Other, specify: ________________________

If at health facility, indicate point of care: ☐Out-Patient / ☐In-Patient

If In-Patient, name of admission ward: ________________________

Date of Admission (dd/mm/yyyy): _____/_____/__________

Date of Discharge (dd/mm/yyyy): _____/_____/__________ [Ç: Look this up in the study facility record]

For SURVEY FIELD WORKER and PATIENT IDENTITY VERIFICATION
Please fill out this section with relevant information from the Facility Recruitment Form (# refers to question number on Recruitment Form).

Patient's Full Name (#2 & #3): ____

Patient's Clan (*mfunda*) Name (#5): ____

Contact Phone Number (#8): ____

Health facility where patient was recruited (See Study ID 2-digit):____________________________

Patient's Sex (#10): ☐ Male ☐ Female

Patient's Age (#11, #11.1): ☐☐ Years **OR** ☐☐ Months (if child is ≤24 months)

Scheduled Date of Interview (DD/MM/YYYY) (#35): ☐☐/☐☐/☐☐☐☐

For INTERVIEWER

Your Name: ____

Have you (interviewer) called to confirm the interview 1 day in advance? ☐ Yes ☐ No

Did you take an empty bucket with you? ☐ Yes ☐ No ☐ None available

Instructions for Interviewer:

*Please check that you have completed the **SES-WASH Survey** before starting this **D-1 Cost-of-Illness Survey, First Interview**.*

Use this instrument to interview an adult (head of household, parent, or guardian) that is familiar with the patient and his/her treatment over the last few days. If the patient is ≥18 years old, attempt to interview the patient unless s/he requires a caretaker (e.g., patient is elderly and/or has mental illness).

*Any places that say "**SCRIPT**" should be read out loud by you, the interviewer, to the patient or person answering on behalf of the patient. Any places that say "**Interviewer**" are notes for you to be aware of before continuing to the next survey question.*

Cholera Surveillance In Malawi (CSIMA) Program

NSANJE DISTRICT USE ONLY

Form S.I.#:__________________ **Study label**

(To be filled by Data Management) **N**☐☐-☐☐☐☐

**Verifying Patient Identity**		
**SCRIPT:** My records show that you (or the patient) went or were taken to the [**health facility**] about 0 to 7 days ago. Can you please tell me your name in the village or your father's name to verify your identify?<br><br>***Interviewer:*** *Please also verify visually the patient's sex as indicated above. The age should be within 1 year.*		
1	Is this correct?	☐ Yes → **continue to 2** ☐ No ☐ Don't know
Interviewer: *The patient (or parent/guardian) should have responded "**Yes**" to question 1. If s/he did not, ask if the patient is another household member or if there has been a mistake. If the problem cannot be resolved and the individual who was treated at the health facility cannot be identified,* ***STOP the interview****.*		
2	Could you please tell me your relationship to the patient?	☐ 1 = Self (patient) ☐ 2 = Mother ☐ 3 = Father<br>☐ 4 = Sister ☐ 5 = Brother ☐ 6 = Grandmother<br>☐ 7 = Grandfather<br>☐ 8 = Other (specify): ____________________

SCRIPT: As you may recall, when you visited the [**health facility**] 0–7 days ago, you were read an informed consent form that described participation in this survey and study. We would like to remind you that there are no direct risks from participation in this study. Participation is free of charge, and will not affect your treatment in any way now nor in the future at any health facility.

The purpose of this current survey is to understand how your (or the patient's) cholera episode has affected him/her financially. The results of this study will help local health staff in improving our knowledge of how cholera affects people in Malawi so that we can be prepared for future cholera outbreaks.

As a thank you for participating in this Cost-of-Illness Survey, we will give you a bucket that you may use in various ways, such as drawing water or for washing your hands at the end of the survey. If you have a **health passport** available, it may be helpful to use it to help you answer these questions.

**SECTION A: Patient History Before Day 0**		
**SCRIPT:** After the doctor examined you clinically / after taking a stool sample recently during your (or the patient's) visit to [**health facility**], the doctor suspected / we found the cholera bacteria present. I am going to ask you about how this recent cholera episode affected you (or the patient) and anyone that may have gone with you (or the patient) while seeking treatment.<br><br>First, I would like to understand how much you (or the patient) had to spend on any healthcare services (including traditional healers) **before** visiting the health facility on [**Day 0**].		
3	How many ***days*** were you (or the patient) sick with diarrhea before going to [**health facility**] 3 to 7 days ago where you were diagnosed?	☐☐ Days<br>☐ 99 = Don't know

Cholera Surveillance In Malawi (CSIMA) Program
NSANJE DISTRICT USE ONLY

Form S.I.#:__________________ **Study label**

(To be filled by Data Management) **N☐☐-☐☐☐☐**

4	Before going to [**health facility**] for care 3 to 7 days ago, did you (or the patient) receive treatment for illness somewhere else?	☐ 1 = Yes<br>☐ 2 = No → **skip to 34 (SECTION C)**<br>☐ 9 = Don't know → **skip to 34 (SECTION C)**
5	How many treatment locations did you visit?<br><br>*Examples of treatment locations: traditional healer, CHAM facility, public facility, and private hospital. Each of these should be counted as separate treatment locations.*	☐☐ Treatment location(s)

SECTION B: Health Facility Visits Before Day 0

Instructions: *Please fill out the entire column (top to bottom) for each time the patient sought care at a treatment location before Day 0. Please provide the following details in* ***local Malawian kwacha (MWK) currency****. If additional days are needed, please use the additional pages provided.*

Questions #6 through #33 *must be filled out for each visit. The first column should be the patient's earliest visit. The last column filled out should be the visit closest to the health facility visit in which the patient was enrolled. Do not include the Day 0 health facility visit.*

Example: *If there was a visit to a traditional healer and a health facility on a single day, then this is 2 visits (2 columns) that must be recorded.*

Item		Visits Before Day 0			
		**Visit 1 (Earliest Visit)**	**Visit 2**	**Visit 3**	**Visit 4**
6	Where did you seek treatment?<br><br>**Code:**<br>1 = District hospital<br>2 = Health centre/post<br>3 = CHAM/private<br>4 = Traditional healer/herbalist<br>5 = Other (specify)<br>9 = Don't know	☐<br>If "**5**" entered, specify:<br>__________	☐<br>If "**5**" entered, specify:<br>__________	☐<br>If "**5**" entered, specify:<br>__________	☐<br>If "**5**" entered, specify:<br>__________
7	During this visit, did you (or the patient) have to buy food and/or water for yourself?	☐ 1 = Yes<br>☐ 2 = No<br>→ **skip to 9**<br>☐ 9 = Don't know<br>→ **skip to 9**	☐ 1 = Yes<br>☐ 2 = No<br>→ **skip to 9**<br>☐ 9 = Don't know<br>→ **skip to 9**	☐ 1 = Yes<br>☐ 2 = No<br>→ **skip to 9**<br>☐ 9 = Don't know<br>→ **skip to 9**	☐ 1 = Yes<br>☐ 2 = No<br>→ **skip to 9**<br>☐ 9 = Don't know<br>→ **skip to 9**
8	How much did you pay for the food and/or water?<br><br>*Write "0" if nothing was paid.*	**MWK**<br>☐ 9 = Don't know	**MWK**<br>☐ 9 = Don't know	**MWK**<br>☐ 9 = Don't know	**MWK**<br>☐ 9 = Don't know

IVI_CSIMA_ v4.1, 24-April-2017

Cholera Surveillance In Malawi (CSIMA) Program
NSANJE DISTRICT USE ONLY

Form S.I.#:__________________ **Study label**

(To be filled by Data Management) **N☐☐-☐☐☐☐**

9	Not including food and/or water, how much did you pay for this visit?<br><br>*Write "0" if nothing was paid.*	**MWK**<br>☐ 9 = Don't know	**MWK**<br>☐ 9 = Don't know	**MWK**<br>☐ 9 = Don't know	**MWK**<br>☐ 9 = Don't know
10	What was this payment for?<br>*(Specify up to 3 choices)*<br><br>**Code:**<br>1 = Medicine/drugs<br>2 = Diagnostic/lab fee<br>3 = Doctor, nurse, or herbalist fee<br>4 = Other (specify)<br>6 = Does not apply<br>9 = Don't know	Response 1: ☐<br>Response 2: ☐<br>Response 3: ☐<br><br>If "**4**" entered, specify:<br>____________	Response 1: ☐<br>Response 2: ☐<br>Response 3: ☐<br><br>If "**4**" entered, specify:<br>____________	Response 1: ☐<br>Response 2: ☐<br>Response 3: ☐<br><br>If "**4**" entered, specify:<br>____________	Response 1: ☐<br>Response 2: ☐<br>Response 3: ☐<br><br>If "**4**" entered, specify:<br>____________
11	Did you pay for anything outside of this treatment location?<br><br>*Example: the healer asked you to buy **roots** for medicinal use after your visit.*	☐ 1 = Yes (specify):<br>____________<br><br>☐ 2 = No<br>→ **skip to 13**<br><br>☐ 9 = Don't know<br>→ **skip to 13**	☐ 1 = Yes (specify):<br>____________<br><br>☐ 2 = No<br>→ **skip to 13**<br><br>☐ 9 = Don't know<br>→ **skip to 13**	☐ 1 = Yes (specify):<br>____________<br><br>☐ 2 = No<br>→ **skip to 13**<br><br>☐ 9 = Don't know<br>→ **skip to 13**	☐ 1 = Yes (specify):<br>____________<br><br>☐ 2 = No<br>→ **skip to 13**<br><br>☐ 9 = Don't know<br>→ **skip to 13**
12	How much did it cost?	**MWK**<br>☐ 9 = Don't know	**MWK**<br>☐ 9 = Don't know	**MWK**<br>☐ 9 = Don't know	**MWK**<br>☐ 9 = Don't know
13	Who paid for the visit?<br>*(Specify up to 3 choices)*	Response 1: ☐<br>Response 2: ☐	Response 1: ☐<br>Response 2: ☐	Response 1: ☐<br>Response 2: ☐	Response 1: ☐<br>Response 2: ☐

IVI_CSIMA_ v4.1, 24-April-2017

Hsiao A, *et al. BMJ Open* 2022; 12:e052337. doi: 10.1136/bmjopen-2021-052337

Cholera Surveillance In Malawi (CSIMA) Program
NSANJE DISTRICT USE ONLY

Form S.I.#:__________________ **Study label**

(To be filled by Data Management) **N☐☐-☐☐☐☐**

	**Code:**<br>1 = Self<br>2 = Patient's family<br>3 = Other (specify), including insurance<br>6 = Did not pay anything<br>9 = Don't know	Response 3: ☐<br>If "**3**" entered, specify:<br>________________	Response 3: ☐<br>If "**3**" entered, specify:<br>________________	Response 3: ☐<br>If "**3**" entered, specify:<br>________________	Response 3: ☐<br>If "**3**" entered, specify:<br>________________
**<u>SCRIPT</u>:** Now, I would like to understand how you **<u>got to the treatment location and then back home</u>**. I will also ask you questions about **<u>whether anyone came with you</u>** to the treatment location.					
14	How did you get to the treatment facility?<br>*(Specify up to 3 choices)*<br>**Code:**<br>1 = On foot/walking<br>2 = Bicycle<br>3 = Motorcycle<br>4 = Minibus<br>5 = Private car<br>6 = Horse cart<br>7 = Boat<br>8 = Other (specify)<br>9 = Don't know	**Travelled by:**<br>Response 1: ☐<br>Response 2: ☐<br>Response 3: ☐<br>If "**8**" entered, specify:<br>________________	**Travelled by:**<br>Response 1: ☐<br>Response 2: ☐<br>Response 3: ☐<br>If "**8**" entered, specify:<br>________________	**Travelled by:**<br>Response 1: ☐<br>Response 2: ☐<br>Response 3: ☐<br>If "**8**" entered, specify:<br>________________	**Travelled by:**<br>Response 1: ☐<br>Response 2: ☐<br>Response 3: ☐<br>If "**8**" entered, specify:<br>________________
15	How long did it take for the patient to get there?	**Travel time:**<br>☐☐☐ **Minutes**	**Travel time:**<br>☐☐☐ **Minutes**	**Travel time:**<br>☐☐☐ **Minutes**	**Travel time:**<br>☐☐☐ **Minutes**
16	How much did it cost for the patient to get there?<br>*Write "0" if nothing was paid.*	**MWK**<br>☐ 999 = Don't know	**MWK**<br>☐ 999 = Don't know	**MWK**<br>☐ 999 = Don't know	**MWK**<br>☐ 999 = Don't know
17	Did you travel to the location <u>alone</u>?	☐ 1 = Yes<br>→ **skip to 20**<br>☐ 2 = No	☐ 1 = Yes<br>→ **skip to 20**<br>☐ 2 = No	☐ 1 = Yes<br>→ **skip to 20**<br>☐ 2 = No	☐ 1 = Yes<br>→ **skip to 20**<br>☐ 2 = No
18	How many people came with you (<u>excluding</u> the patient)?	☐☐ People<br>☐ 99 = Don't know	☐☐ People<br>☐ 99 = Don't know	☐☐ People<br>☐ 99 = Don't know	☐☐ People<br>☐ 99 = Don't know

Cholera Surveillance In Malawi (CSIMA) Program

NSANJE DISTRICT USE ONLY

Form S.I.#:__________________ **Study label**

(To be filled by Data Management) N☐☐-☐☐☐☐

	*If patient answered "No" to 17, this **cannot** be "00"*				
19	How much did it cost for <u>each person</u> that came with you?<br><br>*Write "0" if nothing was paid.*	**MWK Per person**<br>☐ 999 = Don't know	**MWK Per person**<br>☐ 999 = Don't know	**MWK Per person**<br>☐ 999 = Don't know	**MWK Per person**<br>☐ 999 = Don't know
20	Did anybody follow you to the treatment location (did they come separately)?	☐ 1 = Yes<br>☐ 2 = No<br>→ **skip to 25**	☐ 1 = Yes<br>☐ 2 = No<br>→ **skip to 25**	☐ 1 = Yes<br>☐ 2 = No<br>→ **skip to 25**	☐ 1 = Yes<br>☐ 2 = No<br>→ **skip to 25**
21	How many people met you at the location?<br><br>*If patient answered "Yes" to 20, this **cannot** be "00"*	☐☐ People<br>☐ 99 = Don't know	☐☐ People<br>☐ 99 = Don't know	☐☐ People<br>☐ 99 = Don't know	☐☐ People<br>☐ 99 = Don't know
22	How did the person(s) get to the location? *(Specify up to 3 choices)*<br><br>**Code:**<br>1 = On foot/walking<br>2 = Bicycle<br>3 = Motorcycle<br>4 = Minibus<br>5 = Private car<br>6 = Horse cart<br>7 = Boat<br>8 = Other (specify)<br>9 = Don't know	**Travelled by:**<br>Response 1: ☐<br>Response 2: ☐<br>Response 3: ☐<br>If "**8**" entered, specify:<br>____________________	**Travelled by:**<br>Response 1: ☐<br>Response 2: ☐<br>Response 3: ☐<br>If "**8**" entered, specify:<br>____________________	**Travelled by:**<br>Response 1: ☐<br>Response 2: ☐<br>Response 3: ☐<br>If "**8**" entered, specify:<br>____________________	**Travelled by:**<br>Response 1: ☐<br>Response 2: ☐<br>Response 3: ☐<br>If "**8**" entered, specify:<br>____________________
23	How long did it take the person(s)?<br><br>*Enter time <u>per person</u>.*	**Travel time:**<br>☐☐☐<br>**Minutes per person**<br>☐ 999 = Don't know	**Travel time:**<br>☐☐☐<br>**Minutes per person**<br>☐ 999 = Don't know	**Travel time:**<br>☐☐☐<br>**Minutes per person**<br>☐ 999 = Don't know	**Travel time:**<br>☐☐☐<br>**Minutes per person**<br>☐ 999 = Don't know

Cholera Surveillance In Malawi (CSIMA) Program

NSANJE DISTRICT USE ONLY

Form S.I.#:__________________ **Study label**

(To be filled by Data Management) N☐☐-☐☐☐☐

24	How much did it cost the person(s)?<br><br>*Enter cost per person. Write "0" if nothing was paid.*	**MWK Per person**<br><br>☐ 999 = Don't know	**MWK Per person**<br><br>☐ 999 = Don't know	**MWK Per person**<br><br>☐ 999 = Don't know	**MWK Per person**<br><br>☐ 999 = Don't know
25	While at the facility, how much did everyone who came with you or met you at the treatment facility spend on food and/or water (excluding patient)?<br><br>*Enter cost for all persons combined. Write "0" if no food and/or water were purchased.*	**MWK Total for all persons**<br><br>☐ Respondent unsure, but estimated<br><br>☐ 666 = Went alone<br>☐ 999 = Don't know	**MWK Total for all persons**<br><br>☐ Respondent unsure, but estimated<br><br>☐ 666 = Went alone<br>☐ 999 = Don't know	**MWK Total for all persons**<br><br>☐ Respondent unsure, but estimated<br><br>☐ 666 = Went alone<br>☐ 999 = Don't know	**MWK Total for all persons**<br><br>☐ Respondent unsure, but estimated<br><br>☐ 666 = Went alone<br>☐ 999 = Don't know
26	How much did the person(s) spend on lodging (total) if an overnight stay was needed?<br><br>*Enter cost for all persons combined. Write "0" if no overnight stay was needed.*	**MWK Total for all persons**<br><br>☐ 666 = Was alone<br>☐ 999 = Don't know	**MWK Total for all persons**<br><br>☐ 666 = Was alone<br>☐ 999 = Don't know	**MWK Total for all persons**<br><br>☐ 666 = Was alone<br>☐ 999 = Don't know	**MWK Total for all persons**<br><br>☐ 666 = Was alone<br>☐ 999 = Don't know
**SCRIPT:** Now, I would like to understand how you (or the patient) **travelled back home after the visit** to the treatment location.					
27	How did you get home?<br>*(Specify up to 3 choices)*	**Travelled by:**<br><br>Response 1: ☐	**Travelled by:**<br><br>Response 1: ☐	**Travelled by:**<br><br>Response 1: ☐	**Travelled by:**<br><br>Response 1: ☐

Cholera Surveillance In Malawi (CSIMA) Program
NSANJE DISTRICT USE ONLY

Form S.I.#:__________________ **Study label**

(To be filled by Data Management) **N☐☐-☐☐☐☐**

	**Code:**<br>1 = On foot/walking<br>2 = Bicycle<br>3 = Motorcycle<br>4 = Minibus<br>5 = Private car<br>6 = Horse cart<br>7 = Boat<br>8 = Other (specify)<br>9 = Don't know	Response 2: ☐<br>Response 3: ☐<br>If "**8**" entered, specify:<br>________________	Response 2: ☐<br>Response 3: ☐<br>If "**8**" entered, specify:<br>________________	Response 2: ☐<br>Response 3: ☐<br>If "**8**" entered, specify:<br>________________	Response 2: ☐<br>Response 3: ☐<br>If "**8**" entered, specify:<br>________________
28	How long did it take for the patient?	**Travel time:**<br>☐☐☐ **Minutes**<br>☐ 999 = Don't know	**Travel time:**<br>☐☐☐ **Minutes**<br>☐ 999 = Don't know	**Travel time:**<br>☐☐☐ **Minutes**<br>☐ 999 = Don't know	**Travel time:**<br>☐☐☐ **Minutes**<br>☐ 999 = Don't know
29	How much did it cost for the patient?<br>*Write "0" if nothing was paid.*	**MWK**<br>☐ 999 = Don't know	**MWK**<br>☐ 999 = Don't know	**MWK**<br>☐ 999 = Don't know	**MWK**<br>☐ 999 = Don't know
30	Did you travel back to your home <u>alone</u>?	☐ 1 = Yes<br>→ **skip to 33**<br>☐ 2 = No	☐ 1 = Yes<br>→ **skip to 33**<br>☐ 2 = No	☐ 1 = Yes<br>→ **skip to 33**<br>☐ 2 = No	☐ 1 = Yes<br>→ **skip to 33**<br>☐ 2 = No
31	How many people travelled home with you (<u>excluding</u> you)?<br>*If patient answered "No" to 30, this **cannot** be "00"*	☐☐ People<br>☐ 99 = Don't know	☐☐ People<br>☐ 99 = Don't know	☐☐ People<br>☐ 99 = Don't know	☐☐ People<br>☐ 99 = Don't know
32	How much did it cost for <u>each person</u> that came with you?<br>*Write "0" if nothing was paid.*	**MWK Per person**<br>☐ 999 = Don't know	**MWK Per person**<br>☐ 999 = Don't know	**MWK Per person**<br>☐ 999 = Don't know	**MWK Per person**<br>☐ 999 = Don't know
Interviewer: *Please add any additional notes regarding travelling to and from the treatment center in* ***Question #33.***<br><br>***For example:*** *"While patient was at CHAM facility with his/her mother, the patient required food. The mother traveled home by foot to bring the home-cooked food to the CHAM facility for the patient to eat."*					
33	Additional notes regarding patient or companion travel.				

CHECK for Interviewer

Cholera Surveillance In Malawi (CSIMA) Program
NSANJE DISTRICT USE ONLY

Form S.I.#:__________________ **Study label**

(To be filled by Data Management) N☐☐-☐☐☐☐

How many treatment locations did the patient specify in Question #5?

☐☐ Treatment location(s)

Please check that the **number of treatment location(s) matches the number of columns** that you have filled out in **Section B** before proceeding to **Section C**. **Questions #6 through #33** must be filled out for each visit. If there was a visit to a traditional healer and a health facility on a single day, then this is 2 visits (2 columns) that must be recorded.

Have you verified?

☐ Yes → **continue to Section C**

SECTION C: Health Facility Visits Starting at Day 0

SCRIPT: Now, I would like to understand how much you (or the patient) had to spend on any healthcare services (including traditional healers) **starting on the day you first visited the health facility** where we diagnosed and treated you on Day 0.

Instructions: *Please fill out the entire column (top to bottom) for each time the patient sought care at a treatment location starting at **Day 0**. Please provide the following details in **local Malawian kwacha (MWK) currency**. If additional days are needed, please use the additional pages provided.*

Questions #34 through #61 *must be filled out for each visit. The first column should be the patient's first visit to the health facility in which the patient was enrolled (i.e., Day 0). The last column filled out should be the visit closest to today's interview. Do **not** include any visits to any type of treatment location today.*

Item		Visits Starting at Day 0			
		**Day 0**	**Visit 1**	**Visit 2**	**Visit 3**
34	Where did you seek treatment?<br><br>**Code:**<br>1 = District hospital<br>2 = Health centre/post<br>3 = CHAM/private<br>4 = Traditional healer/herbalist<br>5 = Other (specify)<br>9 = Don't know	☐<br>If "**5**" entered, specify:<br>________	☐<br>If "**5**" entered, specify:<br>________	☐<br>If "**5**" entered, specify:<br>________	☐<br>If "**5**" entered, specify:<br>________
35	During this visit, did you (or the patient) have to buy food and/or water for yourself?	☐ 1 = Yes<br>☐ 2 = No → **skip to 37**<br>☐ 9 = Don't know → **skip to 37**	☐ 1 = Yes<br>☐ 2 = No → **skip to 37**<br>☐ 9 = Don't know → **skip to 37**	☐ 1 = Yes<br>☐ 2 = No → **skip to 37**<br>☐ 9 = Don't know → **skip to 37**	☐ 1 = Yes<br>☐ 2 = No → **skip to 37**<br>☐ 9 = Don't know → **skip to 37**
36	How much did you pay for the food and/or water?	**MWK**	**MWK**	**MWK**	**MWK**

IVI_CSIMA_ v4.1, 24-April-2017

Cholera Surveillance In Malawi (CSIMA) Program

NSANJE DISTRICT USE ONLY

Form S.I.#:__________________ **Study label**

(To be filled by Data Management) **N☐☐-☐☐☐☐**

		☐ 9 = Don't know	☐ 9 = Don't know	☐ 9 = Don't know	☐ 9 = Don't know
37	Not including food and/or water, how much did you pay for this visit?<br><br>*Write "0" if nothing was paid.*	**MWK**<br>☐ 9 = Don't know	**MWK**<br>☐ 9 = Don't know	**MWK**<br>☐ 9 = Don't know	**MWK**<br>☐ 9 = Don't know
38	What was this payment for? *(Specify up to 3 choices)*<br><br>**Code:**<br>1 = Medicine/drugs<br>2 = Diagnostic/lab fee<br>3 = Doctor, nurse, or herbalist fee<br>4 = Other (specify)<br>6 = Does not apply<br>9 = Don't know	Response 1: ☐<br>Response 2: ☐<br>Response 3: ☐<br>If "**4**" entered, specify:<br>__________	Response 1: ☐<br>Response 2: ☐<br>Response 3: ☐<br>If "**4**" entered, specify:<br>__________	Response 1: ☐<br>Response 2: ☐<br>Response 3: ☐<br>If "**4**" entered, specify:<br>__________	Response 1: ☐<br>Response 2: ☐<br>Response 3: ☐<br>If "**4**" entered, specify:<br>__________
39	Did you pay for anything outside of this treatment location?<br><br>*Example: the healer asked you to buy **roots** for medicinal use after your visit.*	☐ 1 = Yes (specify):<br>__________<br>☐ 2 = No<br>→ **skip to 41**<br>☐ 9 = Don't know<br>→ **skip to 41**	☐ 1 = Yes (specify):<br>__________<br>☐ 2 = No<br>→ **skip to 41**<br>☐ 9 = Don't know<br>→ **skip to 41**	☐ 1 = Yes (specify):<br>__________<br>☐ 2 = No<br>→ **skip to 41**<br>☐ 9 = Don't know<br>→ **skip to 41**	☐ 1 = Yes (specify):<br>__________<br>☐ 2 = No<br>→ **skip to 41**<br>☐ 9 = Don't know<br>→ **skip to 41**
40	How much did it cost?	**MWK**<br>☐ 9 = Don't know	**MWK**<br>☐ 9 = Don't know	**MWK**<br>☐ 9 = Don't know	**MWK**<br>☐ 9 = Don't know
41	Who paid for the visit?	Response 1: ☐	Response 1: ☐	Response 1: ☐	Response 1: ☐

IVI_CSIMA_ v4.1, 24-April-2017

Cholera Surveillance In Malawi (CSIMA) Program

NSANJE DISTRICT USE ONLY

Form S.I.#:__________________ **Study label**

(To be filled by Data Management) **N☐☐-☐☐☐☐**

	(Specify up to 3 choices)<br><br>**Code:**<br>1 = Self<br>2 = Patient's family<br>3 = Other (specify), including insurance<br>6 = Did not pay anything<br>9 = Don't know	Response 2: ☐<br>Response 3: ☐<br><br>If "**3**" entered, specify:<br>____________	Response 2: ☐<br>Response 3: ☐<br><br>If "**3**" entered, specify:<br>____________	Response 2: ☐<br>Response 3: ☐<br><br>If "**3**" entered, specify:<br>____________	Response 2: ☐<br>Response 3: ☐<br><br>If "**3**" entered, specify:<br>____________
**SCRIPT:** Now, I would like to understand how you **got to the treatment location and then back home**. I will also ask you questions about **whether anyone came with you** to the treatment location.					
42	How did you get to the treatment facility?<br>*(Specify up to 3 choices)*<br><br>**Code:**<br>1 = On foot/walking<br>2 = Bicycle<br>3 = Motorcycle<br>4 = Minibus<br>5 = Private car<br>6 = Horse cart<br>7 = Boat<br>8 = Other (specify)<br>9 = Don't know	**Travelled by:**<br>Response 1: ☐<br>Response 2: ☐<br>Response 3: ☐<br><br>If "**8**" entered, specify:<br>____________	**Travelled by:**<br>Response 1: ☐<br>Response 2: ☐<br>Response 3: ☐<br><br>If "**8**" entered, specify:<br>____________	**Travelled by:**<br>Response 1: ☐<br>Response 2: ☐<br>Response 3: ☐<br><br>If "**8**" entered, specify:<br>____________	**Travelled by:**<br>Response 1: ☐<br>Response 2: ☐<br>Response 3: ☐<br><br>If "**8**" entered, specify:<br>____________
43	How long did it take for the patient to get there?	**Travel time:**<br>☐☐☐ **Minutes**	**Travel time:**<br>☐☐☐ **Minutes**	**Travel time:**<br>☐☐☐ **Minutes**	**Travel time:**<br>☐☐☐ **Minutes**
44	How much did it cost for the patient to get there?<br><br>*Write "0" if nothing was paid.*	**MWK**<br><br>☐ 999 = Don't know	**MWK**<br><br>☐ 999 = Don't know	**MWK**<br><br>☐ 999 = Don't know	**MWK**<br><br>☐ 999 = Don't know
45	Did you travel to the location alone?	☐ 1 = Yes<br>→ **skip to 48**<br><br>☐ 2 = No	☐ 1 = Yes<br>→ **skip to 48**<br><br>☐ 2 = No	☐ 1 = Yes<br>→ **skip to 48**<br><br>☐ 2 = No	☐ 1 = Yes<br>→ **skip to 48**<br><br>☐ 2 = No
46	How many people came with you	☐☐ People	☐☐ People	☐☐ People	☐☐ People

Cholera Surveillance In Malawi (CSIMA) Program

NSANJE DISTRICT USE ONLY

Form S.I.#:__________________ **Study label**

(To be filled by Data Management) **N☐☐-☐☐☐☐**

	(excluding the patient)?<br><br>*If patient answered "No" to 45, this **cannot** be "00"*	☐ 99 = Don't know	☐ 99 = Don't know	☐ 99 = Don't know	☐ 99 = Don't know
47	How much did it cost for each person that came with you?<br><br>*Write "0" if nothing was paid.*	**MWK Per person**<br><br>☐ 999 = Don't know	**MWK Per person**<br><br>☐ 999 = Don't know	**MWK Per person**<br><br>☐ 999 = Don't know	**MWK Per person**<br><br>☐ 999 = Don't know
48	Did anybody follow you to the treatment location (did they come separately)?	☐ 1 = Yes<br>☐ 2 = No<br>→ **skip to 53**	☐ 1 = Yes<br>☐ 2 = No<br>→ **skip to 53**	☐ 1 = Yes<br>☐ 2 = No<br>→ **skip to 53**	☐ 1 = Yes<br>☐ 2 = No<br>→ **skip to 53**
49	How many people met you at the location?<br><br>*If patient answered "Yes" to 48, this **cannot** be "00"*	☐☐ People<br><br>☐ 99 = Don't know	☐☐ People<br><br>☐ 99 = Don't know	☐☐ People<br><br>☐ 99 = Don't know	☐☐ People<br><br>☐ 99 = Don't know
50	How did the person(s) get to the location?<br>*(Specify up to 3 choices)*<br><br>**Code:**<br>1 = On foot/walking<br>2 = Bicycle<br>3 = Motorcycle<br>4 = Minibus<br>5 = Private car<br>6 = Horse cart<br>7 = Boat<br>8 = Other (specify)<br>9 = Don't know	**Travelled by:**<br>Response 1: ☐<br>Response 2: ☐<br>Response 3: ☐<br>If "**8**" entered, specify:<br>____________	**Travelled by:**<br>Response 1: ☐<br>Response 2: ☐<br>Response 3: ☐<br>If "**8**" entered, specify:<br>____________	**Travelled by:**<br>Response 1: ☐<br>Response 2: ☐<br>Response 3: ☐<br>If "**8**" entered, specify:<br>____________	**Travelled by:**<br>Response 1: ☐<br>Response 2: ☐<br>Response 3: ☐<br>If "**8**" entered, specify:<br>____________
51	How long did it take the person(s)?	**Travel time:**<br>☐☐☐<br>**Minutes per person**	**Travel time:**<br>☐☐☐<br>**Minutes per person**	**Travel time:**<br>☐☐☐<br>**Minutes per person**	**Travel time:**<br>☐☐☐<br>**Minutes per person**

IVI_CSIMA_ v4.1, 24-April-2017

Hsiao A, *et al. BMJ Open* 2022; 12:e052337. doi: 10.1136/bmjopen-2021-052337

Form S.I.#:__________________ **Study label**

(To be filled by Data Management) **N☐☐-☐☐☐☐**

	Enter time per person.	☐ 999 = Don't know	☐ 999 = Don't know	☐ 999 = Don't know	☐ 999 = Don't know
52	How much did it cost the person(s)?<br><br>*Enter cost per person. Write "0" if nothing was paid.*	**MWK Per person**<br><br>☐ 999 = Don't know	**MWK Per person**<br><br>☐ 999 = Don't know	**MWK Per person**<br><br>☐ 999 = Don't know	**MWK Per person**<br><br>☐ 999 = Don't know
53	While at the facility, how much did everyone who came with you or met you at the treatment facility spend on food and/or water (excluding patient)?<br><br>*Enter cost for all persons combined. Write "0" if no food and/or water were purchased.*	**MWK Total for all persons**<br><br>☐ Respondent unsure, but estimated<br><br>☐ 666 = Went alone<br>☐ 999 = Don't know	**MWK Total for all persons**<br><br>☐ Respondent unsure, but estimated<br><br>☐ 666 = Went alone<br>☐ 999 = Don't know	**MWK Total for all persons**<br><br>☐ Respondent unsure, but estimated<br><br>☐ 666 = Went alone<br>☐ 999 = Don't know	**MWK Total for all persons**<br><br>☐ Respondent unsure, but estimated<br><br>☐ 666 = Went alone<br>☐ 999 = Don't know
54	How much did the person(s) spend on lodging (total) if an overnight stay was needed?<br><br>*Enter cost for all persons combined. Write "0" if no overnight stay was needed.*	**MWK Total for all persons**<br><br>☐ 666 = Was alone<br>☐ 999 = Don't know	**MWK Total for all persons**<br><br>☐ 666 = Was alone<br>☐ 999 = Don't know	**MWK Total for all persons**<br><br>☐ 666 = Was alone<br>☐ 999 = Don't know	**MWK Total for all persons**<br><br>☐ 666 = Was alone<br>☐ 999 = Don't know
**SCRIPT:** Now, I would like to understand how you (or the patient) **travelled back home after the visit** to the treatment location.					
55	How did you get home?	**Travelled by:**	**Travelled by:**	**Travelled by:**	**Travelled by:**

Cholera Surveillance In Malawi (CSIMA) Program
NSANJE DISTRICT USE ONLY

Form S.I.#:__________________ **Study label**

(To be filled by Data Management) N☐☐-☐☐☐☐

	(Specify up to 3 choices)<br>**Code:**<br>1 = On foot/walking<br>2 = Bicycle<br>3 = Motorcycle<br>4 = Minibus<br>5 = Private car<br>6 = Horse cart<br>7 = Boat<br>8 = Other (specify)<br>9 = Don't know	Response 1: ☐<br>Response 2: ☐<br>Response 3: ☐<br>If "**8**" entered, specify:<br>__________	Response 1: ☐<br>Response 2: ☐<br>Response 3: ☐<br>If "**8**" entered, specify:<br>__________	Response 1: ☐<br>Response 2: ☐<br>Response 3: ☐<br>If "**8**" entered, specify:<br>__________	Response 1: ☐<br>Response 2: ☐<br>Response 3: ☐<br>If "**8**" entered, specify:<br>__________
56	How long did it take for the patient?	**Travel time:**<br>☐☐☐ **Minutes**<br>☐ 999 = Don't know	**Travel time:**<br>☐☐☐ **Minutes**<br>☐ 999 = Don't know	**Travel time:**<br>☐☐☐ **Minutes**<br>☐ 999 = Don't know	**Travel time:**<br>☐☐☐ **Minutes**<br>☐ 999 = Don't know
57	How much did it cost for the patient?<br>*Write "0" if nothing was paid.*	**MWK**<br>☐ 999 = Don't know	**MWK**<br>☐ 999 = Don't know	**MWK**<br>☐ 999 = Don't know	**MWK**<br>☐ 999 = Don't know
58	Did you travel back to your home alone?	☐ 1 = Yes<br>→ **skip to 61**<br>☐ 2 = No	☐ 1 = Yes<br>→ **skip to 61**<br>☐ 2 = No	☐ 1 = Yes<br>→ **skip to 61**<br>☐ 2 = No	☐ 1 = Yes<br>→ **skip to 61**<br>☐ 2 = No
59	How many people travelled home with you (excluding you)?<br>*If patient answered "No" to 58, this **cannot** be "00"*	☐☐ People<br>☐ 99 = Don't know	☐☐ People<br>☐ 99 = Don't know	☐☐ People<br>☐ 99 = Don't know	☐☐ People<br>☐ 99 = Don't know
60	How much did it cost for each person that came with you?<br>*Write "0" if nothing was paid.*	**MWK**<br>**Per person**<br>☐ 999 = Don't know	**MWK**<br>**Per person**<br>☐ 999 = Don't know	**MWK**<br>**Per person**<br>☐ 999 = Don't know	**MWK**<br>**Per person**<br>☐ 999 = Don't know
	Interviewer:** Please add any additional notes regarding travelling to and from the treatment center in **Question #61**.*<br>For example:** "While patient was at CHAM facility with his/her mother, the patient required food. The mother traveled home by foot (30 minutes roundtrip) to bring the home-cooked food to the CHAM facility for the patient to eat."*				
61	Additional notes regarding patient or				

IVI_CSIMA_ v4.1, 24-April-2017

Cholera Surveillance In Malawi (CSIMA) Program
NSANJE DISTRICT USE ONLY

Form S.I.#:__________________ **Study label**

(To be filled by Data Management) **N□□-□□□□**

	companion travel.				

CHECK for Interviewer

SCRIPT: Did you go anywhere else to seek treatment for your cholera since your recent health facility visit 3 to 7 days ago? Remember, treatment locations also include the traditional healer and places other than the health facility where we provided you (or the patient) treatment.

Interviewer: *If the patient says "Yes," please fill out another column starting from* ***Question #34 through #61****. Repeat this process until the patient says "No."*

Have you verified that the patient has not gone anywhere else for care since his/her Day 0 visit?

□ Yes → **continue to Section D**

SECTION D: Indirect Costs: Productivity Losses & Opportunity Costs

SCRIPT: Now, I would like to ask you about the ways you (or the patient's) cholera illness affected those around you. Sometimes, people with cholera feel so sick that they cannot perform any of their usual activities. Instead, they have to rest and stay in bed. Other people around the patient may not be as sick, and can still do their usual activities. But, they may not be able to perform their activities as well as normal because they may have to help or take care of the patient.

When you answer these questions, please think about the entire time period when you were sick with cholera, starting with before you (or the patient) went to the health facility for diagnosis.

62	In **total**, how many days have you (or the patient) been sick with cholera since the symptoms started?	□□ Days	□ 99 = Don't know
63	How many days were you (or the patient) **completely unable** to do any of your usual activities while sick?	□□ Days	□ 99 = Don't know
64	How many days were you (or the patient) able to perform **some but not all** of your usual activities while sick?	□□ Days	□ 99 = Don't know
65	How many days were you (or the patient) able to perform **all** of your usual activities while sick?	□□ Days	□ 99 = Don't know

ERROR CHECK: *Please check that the number of days of no activity, some activity, and normal activity add up to the total number of days ill. If the days do not add up correctly, please review* ***Questions #62 through #65*** *with the patient.*

Days of **no activity** while ill (**Question #63**)	+	Days of **some activity** while ill (**Question #64**)	+	Days of **normal activity** while ill (**Question #65**)	=	Total number of days ill (**Question #62**)
□□ Days	+	□□ Days	+	□□ Days	=	□□ Days

66	What would you (or the patient) be doing if you had not been sick?<br><br>*If the patient is an infant/toddler, select "5" for Leisure or play time. Check all that apply.*	□ 1 = Going to school<br>□ 2 = Working for a wage (e.g. for an employer, paid)<br>□ 3 = Working for self (e.g. fishing or farming, unpaid)<br>□ 4 = Attending church<br>□ 5 = Leisure or play time<br>□ 6 = Helping a parent<br>□ 7 = Housework (e.g. cooking, cleaning, child care)<br>□ 8 = Other (specify): ______________________

IVI_CSIMA_ v4.1, 24-April-2017

Hsiao A, *et al. BMJ Open* 2022; 12:e052337. doi: 10.1136/bmjopen-2021-052337

Cholera Surveillance In Malawi (CSIMA) Program

NSANJE DISTRICT USE ONLY

Form S.I.#:__________________ **Study label**

(To be filled by Data Management) **N☐☐-☐☐☐☐**

67	Do you (or the patient) work for a wage (cash or in-kind)?	☐ 1 = Yes, paid in cash (specify amount **per day**): __________________ **MWK**<br>☐ 2 = Yes, paid in-kind (specify what): __________________<br>☐ 3 = No
68	If you work for a wage, did your (or the patient's) employer or boss pay for the days you were <u>sick but did not come to work</u>? *For example, if the patient has sick leave.*	☐ 1 = Yes<br>☐ 2 = No → **skip to 70**<br>☐ 9 = Don't know → **skip to 70**
69	How many sick days did your (or the patient's) employer or boss pay you for?	☐☐ Days<br>☐ 66 = Patient does not work ☐ 99 = Don't know
70	In total, how many days of income did you (or the patient) lose because of this illness <u>since it started</u>?	☐☐ Days<br>☐ 66 = Patient does not work ☐ 99 = Don't know

**SECTION E: Indirect Costs: Caretakers & Substitute Labor ("Helpers")**		
**<u>SCRIPT</u>:** Now, I would like to understand if anyone helped you (or the patient) while you were sick. This includes 2 types of people:<br>1. Someone who may have been paid to complete your usual tasks for you<br>2. Someone who had to help take care of you (or the patient) while you were sick<br>Sometimes, this is the same person.		
71	Were you (or the patient) so sick with cholera that someone had to help you (or the patient) in any way?<br>*This includes helpers who had to skip their own usual work or activities to help you.*	☐ 1 = Yes<br>☐ 2 = No → **skip to 85 (SECTION F)**<br>☐ 9 = Don't know → **skip to 85 (SECTION F)**
72	How many people cared for you (or the patient) or did your usual activities for you while you were sick with cholera?	☐☐ People ☐ 99 = Don't know

Cholera Surveillance In Malawi (CSIMA) Program
NSANJE DISTRICT USE ONLY

Form S.I.#:__________________ **Study label**

(To be filled by Data Management) **N☐☐-☐☐☐☐**

(SECTION E CONTINUED)						
Interviewer: *Please enter responses for the number of people the patient indicated in* **Question #72**. *For example, if the patient responded "3", then lines A, B, and C should be filled out for each question.*						
**Helper number**	73. Patient's relationship with person who helped him/her while sick.<br><br>*Write "hired help" if this is someone who was paid to perform the patient's usual activities, or was hired to provide care for the person.*	74. Is this helper a member of your household?<br><br>**1** = Yes<br>**2** = No<br>**9** = Don't know	75. How old is this helper?<br><br>**1** = Adult (17+)<br>**2** = Teenager (12–16)<br>**3** = Child (<12)<br>**9** = Don't know	76. How many days or hours did s/he help you (or the patient)?<br><br>*Record the number and circle days or hours.*	77. Was this helper paid to help you (or the patient)?<br><br>**1** = Yes<br>**2** = No<br>**→ skip to 79**<br>**9** = Don't know<br>**→ skip to 79**	78. How much was this helper **paid per day** in Malawian kwacha (MWK) to help the patient?
**A**				**DAYS or HOURS**		**MWK**
**B**				**DAYS or HOURS**		**MWK**
**C**				**DAYS or HOURS**		**MWK**
**D**				**DAYS or HOURS**		**MWK**
**E**				**DAYS or HOURS**		**MWK**

Cholera Surveillance In Malawi (CSIMA) Program

NSANJE DISTRICT USE ONLY

Form S.I.#:__________________ **Study label**

(To be filled by Data Management) **N☐☐-☐☐☐☐**

**Helper number**	79. Did this helper perform:<br><br>**1** = All of the patient's activities<br>**2** = Some of the patient's activities<br>**3** = None of the patient's activities<br>**9** = Don't know	80. Did the helper cut back on his/her own usual activities?<br><br>**1** = Yes<br>**2** = No<br>→ **skip to 83**<br>**9** = Don't know<br>→ **skip to 83**	81. How many days did the helper cut back on his/her own duties?<br><br>*Record the number and circle days or hours.*	82. Was the helper able to do:<br><br>**1** = Some of his/her own activities<br>**2** = None of her/her own activities<br>**9** = Don't know	83. What would the helper have been doing if s/he had not been caring for the sick patient?<br><br>*Indicate all that apply.*<br><br>**1** = Going to school<br>**2** = Working for a wage (paid)<br>**3** = Working for self (unpaid)<br>**4** = Attending church<br>**5** = Leisure or play time<br>**6** = Helping parent<br>**7** = Housework<br>**8** = Other (specify)	84. If helper works for a wage, how much is he/she normally **paid per day** for his/her work in Malawian kwacha (MWK)?
**A**			**DAYS or HOURS**			**MWK**
**B**			**DAYS or HOURS**			**MWK**
**C**			**DAYS or HOURS**			**MWK**
**D**			**DAYS or HOURS**			**MWK**
**E**			**DAYS or HOURS**			**MWK**

CHECK for Interviewer

How many helpers did the patient specify in Question #72?

☐☐ People

Please check that the **number of people matches the number of rows** that you have filled out in **Section E** before proceeding to **Section F. Questions #73 through #84** must be filled out for each helper.

Have you verified?

☐ Yes → **continue to Section F**

SECTION F: Financial Impact of Cholera

SCRIPT: We are almost done with the interview. There are just a few more questions I would like to ask you to understand how this cholera event impacted your (or the patient's) financial situation.

85	Did you (or the patient) or your household have to borrow money from anyone in order to pay for the cholera treatment?	☐ 1 = Yes<br>☐ 2 = No → **skip to 89 (SECTION G)**
86	Who did you (or the patient) or your household borrow money from?	☐ 1 = Family member ☐ 2 = Friend<br>☐ 3 = Church ☐ 4 = Neighbors

Form S.I.#:__________________ **Study label**

(To be filled by Data Management) **N☐☐-☐☐☐☐**

	Check all that apply.	☐ 5 = Village chief ☐ 6 = Boss/employer<br>☐ 7 = Social Grouping or Cooperative<br>☐ 8 = Other (specify): ______________________________
87	Did your (or the patient's) household have to sell any items in order to raise money to pay for the cholera treatment?	☐ 1 = Yes<br>☐ 2 = No → **skip to 89 (SECTION G)**
88	What was sold?<br><br>*Check all that apply.*	☐ 1 = Clothing ☐ 2 = Livestock, specify:________<br>☐ 3 = Radio ☐ 4 = Furniture<br>☐ 5 = Other (specify): ______________________________

**SECTION G: Conclusion & Scheduling Next Interview**		
**SCRIPT:** Now, I have some final questions about your (the patient's) current health status.		
89	Compared to how you felt before this illness, how is your overall physical wellness today?	☐ 1 = Worse<br>☐ 2 = The same<br>☐ 3 = Better
90	Do you feel that you (or the patient) has fully recovered from cholera?	☐ 1 = Yes → **continue to SECTION H to Conclude Interview**<br>☐ 2 = No → **continue to below script**<br>☐ 3 = Not sure → **continue to below script**
**SCRIPT:** It sounds like you are still feeling sick because of cholera. So, we would like to follow-up in approximately 7 days with another interview to check on your health. For the next interview, you will not need to answer the questions again about your living arrangements, household belongings, and water, sanitation, and hygiene practices.		

Hsiao A, *et al. BMJ Open* 2022; 12:e052337. doi: 10.1136/bmjopen-2021-052337

Cholera Surveillance In Malawi (CSIMA) Program

NSANJE DISTRICT USE ONLY

Form S.I.#:__________________ **Study label**

(To be filled by Data Management) N☐☐-☐☐☐☐

During the second interview, we will only ask you questions about any health services you have tried to seek between today and the next interview. The survey will be almost exactly like this one. If you complete that second survey, we will give you a bar of soap for your time and participation.

What is a convenient time for you? We will attempt to contact you at least 1 day in advance to remind you of the interview.

91	Date for second interview	**(DD/MM/YYYY):** ☐☐/☐☐/☐☐☐☐<br>**(HH:MM, 12-hr format):** ☐☐:☐☐ **AM** ☐ **PM** ☐

SCRIPT: At this second interview, I will ask you again to think about any places you have gone to seek additional treatment or care for this episode of cholera.

To help you remember these visits, here is a diary card where you can record basic information about the visit. If you are unable to fill out the card, please let me know. You will still receive a bar of soap at the next interview even if you do not fill out this diary card.

Interviewer: *Show the diary card to the patient and instruct the patient (or someone who can fill the card out on behalf of the patient) on how to fill out the card. Please ensure that they have a pen to fill it out. If they do not, please give them a pen. If it is afternoon when you are finishing the interview, you may want to ask if the patient has visited any place for treatment or care today. If the patient says "**yes**," you may as an example walk the patient (or person filling out card) through the card to begin to fill out today.*

92. Was the patient (or household member) given the diary card to fill out?

☐ Yes ☐ No (specify reason): ____________________

Section H: Incentive & Thanking Patient

SCRIPT: Thank you very much for taking the time to answer my questions. That concludes our interview. To thank you for your time, here is a bucket that you may use as you like. We hope that it may be helpful for your household.

Section I: For SURVEY FIELD WORKER

I1. Interviewer Name: ____________________

Interviewer Signature: ____________________

I2. Date of Interview Completion (DD/MM/YYYY): ☐☐/☐☐/☐☐☐☐

I3. Please indicate any additional interview details:

☐ Interview completed in full ☐ Patient declined interview

☐ Patient not home ☐ Could not verify patient identity

☐ Patient asked to reschedule (fill out below) ☐ Other: ____________________

Hsiao A, *et al. BMJ Open* 2022; 12:e052337. doi: 10.1136/bmjopen-2021-052337

Cholera Surveillance In Malawi (CSIMA) Program
NSANJE DISTRICT USE ONLY

Form S.I.#:__________________ **Study label**

(To be filled by Data Management) **N□□-□□□□**

***** If a second interview has been schedule, please be sure to transfer and record the scheduled interview date from Question #91 to the D-2 Cost-of-Illness Survey form. *****

Section J: Rescheduling Interview

***** If the interview must be rescheduled for any other reason (e.g., person not home, person wants to continue interview at a later date, person does not want to be interviewed today), please also enter a date below. *****

J1. Date of Rescheduled Interview (DD/MM/YYYY): □□/□□/□□□□

J2. *Interviewer:* *Please use this section to write any important details about the interview and/or follow-up interviews for this patient that you feel would be important to remember.*

____

____

____

Hsiao A, *et al. BMJ Open* 2022; 12:e052337. doi: 10.1136/bmjopen-2021-052337
