# Supplementary material for: Economic impact of cholera in households in rural southern Malawi: a prospective study
Source: BMJ Open. 2022 Jun 1;12(6):e052337. doi: 10.1136/bmjopen-2021-052337 (PMC9161053; doi:10.1136/bmjopen-2021-052337)
Supplement: Author's manuscript [file bmjopen-2021-052337.draft_revisions.pdf]

BMJ Open

BMJ Open is committed to open peer review. As part of this commitment we make the peer review history of every article we publish publicly available.

When an article is published we post the peer reviewers' comments and the authors' responses online. We also post the versions of the paper that were used during peer review. These are the versions that the peer review comments apply to.

The versions of the paper that follow are the versions that were submitted during the peer review process. They are not the versions of record or the final published versions. They should not be cited or distributed as the published version of this manuscript.

BMJ Open is an open access journal and the full, final, typeset and author-corrected version of record of the manuscript is available on our site with no access controls, subscription charges or pay-per-view fees (http://bmjopen.bmj.com).

If you have any questions on BMJ Open's open peer review process please email info.bmjopen@bmj.com

Cost-of-illness of cholera in rural southern Malawi from the household perspective

Journal:	*BMJ Open*
Manuscript ID	bmjopen-2021-052337
Article Type:	Original research
Date Submitted by the Author:	13-Apr-2021

Complete List of Authors:	Hsiao, Amber; Technische Universität Berlin Fakultät Wirtschaft und Management, Department of Health Care Management<br>Ramani, Enusa; International Vaccine Institute, Policy and Economic Research ; Technische Universität Berlin Fakultät Wirtschaft und Management, Department of Health Care Management<br>Seo, Hye-jin; International Vaccine Institute, Epidemiology, Public Health, Impact<br>Pak, GiDeok; International Vaccine Institute, Biostatistics & Data Management<br>Vuntade, Dan ; University of Malawi the Polytechnic, Department of Environmental Health<br>M'bang'ombe, Maurice; Ministry of Health Malawi<br>Ngwira, Bagrey; University of Malawi the Polytechnic, Department of Environmental Health<br>Quentin, Wilm; Technische Universität Berlin Fakultät Wirtschaft und Management, Department of Health Care Management<br>Marks, Florian ; International Vaccine Institute, Epidemiology, Public Health, Impact; Cambridge Institute of Therapeutic Immunology and Infectious Disease, Cambridge School of Clinical Medicine, Cambridge Biomedical Campus<br>Mogasale, Vittal; International Vaccine Institute, Policy and Economic Research

Keywords:	Public health < INFECTIOUS DISEASES, HEALTH ECONOMICS, Health policy < HEALTH SERVICES ADMINISTRATION & MANAGEMENT, Economics < TROPICAL MEDICINE

SCHOLARONE™ Manuscripts

BMJ

I, the Submitting Author has the right to grant and does grant on behalf of all authors of the Work (as defined in the below author licence), an exclusive licence and/or a non-exclusive licence for contributions from authors who are: i) UK Crown employees; ii) where BMJ has agreed a CC-BY licence shall apply, and/or iii) in accordance with the terms applicable for US Federal Government officers or employees acting as part of their official duties; on a worldwide, perpetual, irrevocable, royalty-free basis to BMJ Publishing Group Ltd ("BMJ") its licensees and where the relevant Journal is co-owned by BMJ to the co-owners of the Journal, to publish the Work in this journal and any other BMJ products and to exploit all rights, as set out in our licence.

The Submitting Author accepts and understands that any supply made under these terms is made by BMJ to the Submitting Author unless you are acting as an employee on behalf of your employer or a postgraduate student of an affiliated institution which is paying any applicable article publishing charge ("APC") for Open Access articles. Where the Submitting Author wishes to make the Work available on an Open Access basis (and intends to pay the relevant APC), the terms of reuse of such Open Access shall be governed by a Creative Commons licence – details of these licences and which Creative Commons licence will apply to this Work are set out in our licence referred to above.

Other than as permitted in any relevant BMJ Author's Self Archiving Policies, I confirm this Work has not been accepted for publication elsewhere, is not being considered for publication elsewhere and does not duplicate material already published. I confirm all authors consent to publication of this Work and authorise the granting of this licence.

For peer review only

Cost-of-illness of cholera in rural southern Malawi from the household perspective

Amber Hsiao,[1] Enusa Ramani,[1,2] Hye Jin Seo,[2] Gi Deok Pak[2], Dan Vuntade[3], Maurice M'bang'ombe,[4] Bagrey Ngwira,[3] Wilm Quentin,[1] Florian Marks,[2,5,6] Vittal Mogasale[2*]

[1] Technische Universität Berlin, Department of Health Care Management, Berlin, Germany

[2] International Vaccine Institute, Seoul, Republic of Korea

[3] University of Malawi, The Polytechnic, Department of Environmental Health, Blantyre, Malawi

[4] Ministry of Health Malawi, Lilongwe, Malawi

[5] Cambridge Institute of Therapeutic Immunology and Infectious Disease, University of Cambridge School of Clinical Medicine, Cambridge Biomedical Campus, Cambridge, UK, CB20AW

[6] University of Antananarivo, Antananarivo, Madagascar

***Corresponding Author:**

Vittal Mogasale, MBBS, MPH, PhD

International Vaccine Institute, Policy & Economic Research Department,

SNU Research Park, 1 Gwanak-ro, Gwanak-gu Seoul 08826. The Republic of Korea.

Email: vmogasale@ivi.int

T +82 2 881 1379; F + 82 2 872 2803

Word count (excluding title page, abstract, references, tables/figures)

For peer review only - http://bmjopen.bmj.com/site/about/guidelines.xhtml

Abstract

Introduction: Cholera remains a significant contributor to diarrheal illness, especially in sub-Saharan Africa. Few studies have estimated the cost of illness (COI) of cholera in Malawi, a cholera-endemic country. The present study estimated the COI of cholera in Nsanje, southern Malawi, as part of the Cholera Surveillance in Malawi (CSIMA) program following a mass cholera vaccination campaign in 2015.

Methods: Patients ≥12 months of age who were recruited as part of CSIMA were invited to participate in the COI survey. The COI tool captured household components of economic burden, including direct medical and non-medical costs, and indirect lost productivity costs.

Results: Between April 2016 to March 2020, 40 cholera cases were enrolled in the study, all of whom participated in the COI survey. Only two patients had any direct medical costs and five patients reported lost wages due to illness. The COI per patient was $8.94, more than half of which was from direct non-medical costs from food, water, and transportation to the health center.

Conclusion: For the majority of Malawians who struggle to subsist on less than $1 a day, the COI of cholera represents a significant cost burden to families. While cholera treatment is provided for free in government-run health centers, additional investments in cholera control and prevention at the community level and financial support beyond direct medical costs may be necessary to alleviate the economic burden of cholera on households in southern Malawi.

For peer review only - http://bmjopen.bmj.com/site/about/guidelines.xhtml

Strengths and limitations of this study

- This study provides out-of-pocket expenses for receiving treatment for cholera based on the survey of patients and their caregivers providing household perspectives from the field settings.
- The sample size availed for the survey and collection of cost of illness was small that limits the generalizability of the findings.
- The sample size of cholera cases that were hospitalized or treated in private health facilities was low to draw any meaningful conclusion on out-of-pocket expenses.

For peer review only

Introduction

Cholera is an infection of the intestine caused by the bacterium *Vibrio cholerae* that leads to acute, rapidly dehydrating diarrheal disease. Annually, an estimated 2.86 million cases of cholera occur that result in 95,000 deaths.[1] A recent study estimated that cholera is especially pervasive throughout sub-Saharan Africa (sSA) where the human and economic burden of cholera is greatest, with more than 200 million people living in areas where cholera is present. Of these 200 million, 87 million live in areas with high incidence, defined as >1 case per 1,000 people per year.[2]

While improvements to water, sanitation, and hygiene (WASH) are the best long-term solution for preventing more diarrheal diseases, these measures remain unavailable to large proportions of people in sub-Saharan Africa, especially among those living in flood-prone rural areas. The availability of low-cost inactivated oral cholera vaccines (OCV) since 2011 has significantly changed the landscape and provided a cost-effective short-to-middle-term approach for cholera prevention and control.[3] A number of mass vaccination campaigns—many of which have taken place in sSA—have demonstrated that it can be successfully administered in a variety of settings, including high-risk settings to prevent an impending outbreak, or to quickly halt the transmission of an ongoing outbreak.[4 5]

In Malawi specifically, cholera has been reported since the 1970s and is endemic to the country.[6-8] The extent of the cholera burden in the country has not been well documented due to the lack of sustainable diagnostic and surveillance capacity. Nevertheless, in recognition of the

For peer review only

high incidence of cholera and the need for additional tools to address the burden, in 2017, the government of Malawi integrated OCVs into its national cholera control plan,[9] and there have been several OCV campaigns implemented since.[10 11]

One of the worst cholera outbreaks to date occurred in 2015 following the nation's worst floods in history, which affected more than 1.1 million people in the Southern Region and displaced at least 230,000 people from their homes.[12] Within a month of the flood, cholera cases were being reported in Nsanje and Chikwawa, two of the worst-hit districts in southern Malawi. The flooding led to a multi-stakeholder response to coordinate a reactive OCV mass vaccination campaign in the region.[10] Due to the lack of diagnostic capacity, cholera cases were being diagnosed based on clinical presentation as per the World Health Organization (WHO) and national guidelines.[13]

The OCV mass vaccination campaign was then implemented primarily in Nsanje, which was identified as having the highest burden of cholera based on prior years' data provided by the Malawian Ministry of Health (MOH). From March 30, 2015, to April 23, 2015, the International Vaccine Institute (IVI), MOH, WHO, and John Snow, Inc. coordinated a reactive mass vaccination campaign that targeted 160,000 people (320,000 total doses of the OCV Shanchol® (Shantha Biotechnics Pvt., Ltd., India) over 1 year of age. Those living in high-risk areas of Nsanje—namely, those living in internally displaced camps and surrounding villages—were the primary targets.[10]

In the course of a 4-year effort to strengthen cholera surveillance capacity and infrastructure in Nsanje following the mass vaccination campaign, we investigated the cost-of-illness (COI) of

For peer review only

For peer review only - http://bmjopen.bmj.com/site/about/guidelines.xhtml

cholera for patients who sought care. Only one study to date has reported on the cost-of-illness from cholera in Malawi (Lake Chilwa in the southeastern area).[14] The objective of this study is to provide additional field data on the economic burden of cholera in Malawi from a household perspective.

METHODS

As part of the Cholera Surveillance in Malawi (CSIMA) program, cholera surveillance began across all Nsanje (n=22; population >292,000) and Chikwawa (n=18; population >533,000) healthcare facilities from April 5, 2016, to March 31, 2020. IVI staff trained health surveillance assistants (HSAs) and health workers at all Nsanje and Chikwawa facilities to identify patients who sought care for acute watery diarrhea. HSAs are community health facilitators that live in the area served by the local healthcare facility who are involved in activities such as WASH and general health promotion.

Patients who met the inclusion criteria (at least 12 months of age at the start of the vaccination campaign, and presented to a Nsanje health facility with acute watery diarrhea indicative of cholera as defined by the WHO[13]) were invited to participate in the diarrheal surveillance study. Participants who provided informed written consent provided a stool specimen to perform a rapid diagnostic test (RDT) for cholera (Crystal® VC RDT; Arkray Healthcare Pvt., India; previously Span Diagnostics, Surat, India) and/or a stool sample for culture confirmation at the national reference laboratory in Lilongwe. The use of RDTs has been accepted for use by the Malawi MOH, and there is documented experience with this test kit in other settings.[15 16] While

For peer review only - http://bmjopen.bmj.com/site/about/guidelines.xhtml

the use of the RDT is not intended to be a substitute for stool culture, the use of the kit in this setting ensured that one would not miss any potential cholera cases during the early surveillance period while logistics for stool transportation and culture were being set-up.

Between April 6, 2016, and December 12, 2017, culture-confirmed and RDT-positive cholera cases were recruited for the overall study, as well as the COI survey component. However, due to low recruitment numbers of RDT- and/or culture-confirmed cholera cases, in part due to limited availability of RDT kits and stool collection logistical challenges, the inclusion criteria were expanded to include clinically-diagnosed cholera cases in an outbreak situation to avoid missing potential cases.

While cholera vaccines were administered to residents in both Nsanje and Chikwawa, the majority of doses were administered to residents of Nsanje. Thus, only patients recruited from Nsanje health centers were invited for the COI portion of the study. The COI from a household perspective was assessed for patients who provided written informed consent (regardless of vaccination status). The COI tool was developed based on a tool that IVI had previously used for other COI studies, with further refinements using the guidelines for cost collection in cholera studies provided by the WHO.[17 18] The survey tool captured household components of economic burden, divided further into direct medical costs (DMC), direct non-medical costs (DNMC), and indirect costs (IC) (as done in the previous studies[19]), with the payer being the patients themselves or caregivers/helpers of the patient (e.g., relatives). Experienced interviewers were trained on how to administer the survey and all survey responses were validated by the

For peer review only - http://bmjopen.bmj.com/site/about/guidelines.xhtml

University of Malawi Polytechnic and MOH collaborators to ensure they were appropriate for the local context.

A series of a maximum of three interviews were planned for the COI assessment: at day 0–7, day 13–15 (beyond the mean duration of an episode of cholera illness), and day 20-22. To minimize recall bias, the first interview took place as soon as cholera was confirmed either by a test or a clinician, with a maximum of 7 days following the first healthcare facility visit. If a patient preferred to be interviewed while at the healthcare facility, the trained interviewer conducted the first COI interview at the time of the initial outpatient or inpatient visit. Patients who were not feeling well enough were contacted by an HSA to schedule a future interview time. Trained interviewers would then visit the patient's home at the scheduled time to conduct the first interview. At the end of each COI assessment, patients were asked whether they felt they had fully recovered from cholera. If the patient reported still being ill, the next interview was scheduled; otherwise, no additional interviews were conducted.

At each of the interviews, patients (or parent/guardian) were asked how much money they spent during each of their visits to a healthcare facility or other treatment locations (e.g., a traditional healer) since becoming ill, including any visits that may have occurred prior to the study enrollment date. DMCs included out-of-pocket payments for services, prescription drugs, and any other health facility fees. DNMCs included transportation and food expenses incurred by the patient and/or caregivers who went to the health facility with the patient.

For peer review only

Finally, IC accounted for the patient's and/or caregivers' productivity losses incurred due to lost wages. We calculated productivity losses using three scenarios based upon (1) self-reported wage losses, (2) minimum wage per day for all workers between 14–50 years of age (the legal minimum and old-age pension eligibility age[20]) who did not report wage losses, and (3) minimum wage per day for all patients >5 years of age who did not report wage losses, given evidence from Africa that children are often involved in economic activities that add to a family's income.[21]

In all scenarios, if patients and/or caregivers reported wage losses, these were calculated based on self-reported daily lost productivity (half or full days of work lost due to taking care of the ill patient) and daily wages. If no wage loss was reported, we applied the minimum wage of 962 Malawian kwacha (MWK) per day ($1.30USD/day) to account for the monetary value of productivity losses.[22] Productivity losses were not calculated for caregivers who indicated that they did not cut back on their usual activities had they not been caring for the patient.

Costs were collected in Malawian kwacha (MWK) and converted to 2019 USD (738.851 MWK=$1 USD). All analyses were conducted in Stata/IC 13.1 (College Station, TX) and Microsoft Excel.

Ethical approvals

This study was approved by the National Health Sciences Research Committee (NHSRC) of the Ministry of Health in Malawi (protocol #16/2/1547; approval #1547) on March 29, 2016. IVI's Institutional Review Board (IRB) approved the study on April 6, 2016. All participants (or a

For peer review only - http://bmjopen.bmj.com/site/about/guidelines.xhtml

parent/guardian) provided written informed consent prior to interviews. The IVI IRB and the NHSRC approved the modification to expand to inclusion of clinically-diagnosed cases on May 31, 2017, and July 31, 2017, respectively. Training of all HSAs on the modification occurred on December 12 and 13, 2017, and the data collection continued until March 31, 2020.

Patient and Public Involvement

Patients including their caretakers were involved in this study as participants in the cost of illness interviews. Cost related to cholera illness were collected from the participants after obtaining informed consent. The public was not involved in this study. Patients and the public were not involved in study development, defining the research question and study design, recruitment, and the conduction of the study. Early findings of the study were disseminated through a workshop attended by country policymakers, health managers, and health facility staff.

RESULTS

During our surveillance period, 2,201 individuals met the diarrheal illness criteria for stool collection and testing for cholera. Of these patients, 40 (1.8%) were identified as cholera cases and were recruited for the surveillance study. All 40 patients also consented for COI interview. Table 1 summarizes selected demographic characteristics, as well as OCV vaccination statuses, of all cases.

Of the 40 cases, 31 (78%) were seen in an outpatient setting. Duration of illness was 5 days (min–max: 2–17 days) in the outpatient setting and 8 days (min–max: 2–19 days) in the inpatient setting. A higher proportion of inpatient cases were males, whereas outpatient cases were

For peer review only

For peer review only - http://bmjopen.bmj.com/site/about/guidelines.xhtml

majority female. Of the 40 cases, 4 were identified clinically, 9 using RDT, and 27 using culture confirmation. A larger proportion of children were seen in the inpatient setting, compared with outpatient. The majority of patients (n=33) recovered within 7 days after becoming symptomatic.

The direct and indirect cost of cholera per patient was $8.94, with the largest proportion of costs being from direct non-medical costs (food, water, and transportation). Direct medical costs for drugs and consumables was zero for nearly all patients; only 2 patients seen in the inpatient setting had any drug costs of $8.12 (6,000 MWK) and $1.08 (800 MWK) each, both of whom were seen at a private CHAM facility; these two patient costs increased the overall average across the cohort to $0.23 as shown in Table 2.

Patient and caregiver food costs were $0.66 and $1.22 on average, respectively. Roundtrip transportation costs were relatively lower for patients at $1.17, compared to caregiver transportation costs at $1.92. Transportation time on average per patient was 112 minutes roundtrip (min, max: 10, 360).

We also performed various scenario analyses for indirect costs (IC) (Table 3) where costs were only averaged across patients and caregivers with non-zero productivity losses in each scenario. Eight patients were excluded from wage loss calculations due to missing age or birthdate. Thirty-four patients had at least one caregiver provide care during their illness, with a total of 53 caregivers across all patients. Caregivers only reported whether they were children or adults; (i.e., exact ages or birthdates were not ascertained); all caregivers reported being adults who we assumed were of working age.

For peer review only - http://bmjopen.bmj.com/site/about/guidelines.xhtml

In scenario 1, productivity losses (lost wages) due to being sick for the duration of illness were $6.90 on average (n=5) for patients who reported wage losses. Only 15 caregivers (who cared for 8 patients) reported $5.70 on average in lost wages due to missing work. In scenario 2 where the minimum wage was assumed for all patients (n=12) and caregivers (n=45) of working age (n=12), productivity losses were $6.02 for patients and $3.37 for caregivers on average. In scenario 3, where the minimum wage was assumed for all patients >5 years of age (n=17), productivity losses were $6.78 for patients on average. Caregiver costs in scenarios 2 and 3 were the same ($3.37) since all caregivers were adults.

Half of all patients, and two-thirds of inpatients, reported having to borrow money from someone to pay for health care, food, or transportation as a result of having cholera (Table 4), but most did not have to sell personal belongings to pay for care. Food insecurity was an issue for nearly all of the patients (n=35).

DISCUSSION

Our study estimated patient and caregiver costs associated with seeking healthcare for the treatment of severe diarrheal illness diagnosed as cholera in Nsanje. We had few diarrheal cases overall. The cost of cholera illness per patient was $8.94, with the largest proportion of costs being from direct non-medical costs (food, water, and transportation), followed by indirect productivity losses ($3.74) in our primary analysis where costs were averaged across all patients in our cohort.

For peer review only - http://bmjopen.bmj.com/site/about/guidelines.xhtml

For peer review only

The majority of our patients were not admitted during their visit to the health center. Patients who sought care in the outpatient setting were symptomatic an average of 1.4 days prior to seeking care. Patients who were admitted were sick for an average of 2.8 days prior to seeking care and had an inpatient stay of 2 days. Our patient population was slightly skewed toward females and of those whose age was reported, most patients were adults aged 18 and older. The majority of patients were not vaccinated during the OCV campaign in 2015.

Overall, only two cholera patients had any DMCs; one was seen in the outpatient setting (800 MWK, or $1.08) and the other was admitted to a private CHAM facility (6000 MWK, or $8.12). On average, patients and their caregivers spent $5.20 in direct costs ($0.23 in medical costs and $4.97 in non-medical costs, including those with zero costs), >95% of which were due to food and/or water and transportation costs. In our scenario analysis when only those with self-reported wages are considered, only 5 cholera patients (all outpatient cases) reported productivity losses of $6.90 on average, and 15 caregivers of patients reported wage losses, averaging $5.74. In our scenario analysis where we applied the minimum wage to all patients and caregivers who theoretically could be contributing to economic activities (i.e., household work), indirect productivity losses were as high as $6.78 per patient and $3.37 per caregiver.

The overall costs found in our study are similar to or substantially less than one study previously reported in rural Malawi,[14] though, since only two patients had any drug and consumable costs in our population, they are difficult to compare. Ilboudo et al. also reported a much larger proportion of hospitalized cases: their population had 100 cases overall, 90 of which were hospitalized, whereas we only had 7 hospitalized cases (only one of whom reported drugs and

For peer review only - http://bmjopen.bmj.com/site/about/guidelines.xhtml

consumables costs). Transportation costs in our study were also lower for both patients and caregivers compared with their study, which may be attributable to differences in transportation methods and travel distances to health centers.

The largest differences in costs between our study and Ilboudo et al. were in food and lost productivity costs, likely also driven by the fact that their study had more hospitalized cases that required more expenses over several days. Their reported indirect costs of patient's productivity were a larger portion of overall costs (35.1% compared to our 9.6%), which may be attributed to their study cohort having longer inpatient stays. The caregiver lost productivity costs as a proportion of overall costs were lower in their study (23.5% compared to our 32.2%). Differences in productivity costs may be attributed to several reasons, such as differences in wages in Nsanje versus Machinga and Zomba districts, unemployment rates, and other socioeconomic factors. Given the relatively small sample size in both studies, however, more data is needed from the region to understand why these costs differed more than other cost components.

We also assessed financial and food insecurity for cases. Half of our cases had to borrow money from someone to pay for costs associated with their cholera illness and treatment, which is similar to the proportion reported by Ilboudo et al. A large proportion of our cases also faced food insecurity at some point in the prior year.

Our study had several limitations. Given the small number of cholera cases, even after expanding the case criteria to clinically-diagnosed cases (of which there were only 4), it may not be

For peer review only - http://bmjopen.bmj.com/site/about/guidelines.xhtml

representative of the general Nsanje population, particularly those who were sick and did not seek care. It is also possible that patients may have shown up for care but were undiagnosed or misdiagnosed for reasons such as diagnostic sensitivity. While we were unable to assess any patterns or variation in the use of the three testing methods (RDT, culture, or clinically-identified) by the health center, it is possible that our recruited patient cohort is biased toward health centers that were better equipped to test, recruit, and treat patients. For example, many of the more remote health centers in Nsanje lacked toilets or latrines for proper stool collection and have limited access to electricity to ensure the viability of stool samples, which may have limited the sensitivity of the stool culture result. Apart from the logistical challenges of recruitment, the area also has a high number of migrants from Mozambique; migrants may be less likely to seek care due to fear of deportation.

Health facility costs were also not included in our costing, which may be a significant portion of the total costs as most patients reported $0 in direct medical costs (since cholera treatment is provided free of charge in Malawi). A recent study in rural Malawi estimated that the average treatment cost to a health facility for a hospitalized cholera case was $59.70.[14] A separate study on cholera treatment costs at the facility level at this study site has estimated an economic cost of $41.86 and a financial cost of $25.43 in US$ 2018.[23] Finally, there was the potential for recall bias, especially for patients who may have had costs prior to the initial visit to the health center from which they were recruited for the study. For costs incurred from the day of enrollment onward, we attempted to minimize the potential for recall bias by following up first within a week of the initial visit.

For peer review only - http://bmjopen.bmj.com/site/about/guidelines.xhtml

Despite the limitations, our study provides one of the few cost of illness studies of cholera illness in sub-Saharan Africa from the household perspective. More studies are needed to more fully evaluate the cholera costs of illness in Malawi. Currently, spending on health accounts for less than 10% of country revenues—below the 15% target set by the Abuja Declaration to which Malawi is a signatory.[24] Funding is insufficient to meet the basic health needs of Malawians at $28 per capita—only 60% of what is necessary to meet its stated goals for the Malawi Health Sector Strategic Plan of 2011–2016.[25] Our study supports the need for further spending in the health sector; for example, building and staffing additional health centers that reduce the amount of time patients have to travel could reduce the cost burden on patients. Our surveillance efforts may also be a positive indication that the cholera vaccine was an effective method to reduce the burden of cholera in the middle-term, as most of our cases were unvaccinated (approximately half of the Nsanje population received the cholera vaccine). Alternatively, given that cholera often occurs cyclically, it is possible that the time period during which surveillance occurred was a low or no cholera transmission interval. Ultimately, investing more heavily in WASH infrastructure would likely be the a most effective, though more costly, solution in the long-run to avert cholera.

In a country where the gross domestic product per capita per day is less than $1 ($339 annually in 2017 USD), a family who has to spend nearly $9 on average for cholera illness will very likely have to make financial sacrifices to access treatment. Even though the direct medical costs associated with cholera care are low, the patient (who is willing and able to go to the health center) must bear a high burden of direct non-medical and indirect lost productivity costs. For those who do not have the means of even going to a health facility to seek care, the consequences

For peer review only - http://bmjopen.bmj.com/site/about/guidelines.xhtml

are likely even more severe; for example, the death of a family member whose wages supported the family has significant long-term impacts beyond a single cholera episode.

In conclusion, we find that households face a significant cost burden when one falls sick with cholera. These costs are significantly high to warrant additional investments in the health system to avert future cases of cholera.

Contributorship statement

AH and VM conceptualized the study, led the protocol development, field training, and survey implementation in collaboration with HJS, GDP, DV, MM, BN, FM, & ER. Data collection was supervised by DV, MM, and BN, and monitored by GDP, AH, VM, ER, and HJS. Data analysis and interpretation were done by AH supported by VM and WQ. The manuscript was written by AH, supported by VM and WQ. All authors have reviewed, edited, and approved the final manuscript.

Data sharing statement

No additional data available

Competing interests

None declared.

Acknolwedgements

We thank cholera patients and their caregivers for providing valuable data on costs related to the illness and all health care providers who directly or indirectly helped with this research.

Funding

For peer review only - http://bmjopen.bmj.com/site/about/guidelines.xhtml

This research was conducted by International Vaccine Institute and funded by Bill & Melinda Gates Foundation [OPP38590] and the UK Foreign, Commonwealth, and Development Office and Wellcome [214662/Z/18/Z]. The International Vaccine Institute acknowledges its donors including the Republic of Korea, Swedish International Development Cooperation Agency, and the Republic of India.

For peer review only

For peer review only - http://bmjopen.bmj.com/site/about/guidelines.xhtml

REFERENCES

1. Ali M, Nelson AR, Lopez AL, et al. Updated global burden of cholera in endemic countries. *PLoS neglected tropical diseases* 2015;9(6):e0003832. doi: 10.1371/journal.pntd.0003832 [published Online First: 2015/06/05]
2. Lessler J, Moore SM, Luquero FJ, et al. Mapping the burden of cholera in sub-Saharan Africa and implications for control: an analysis of data across geographical scales. *Lancet (London, England)* 2018;391(10133):1908-15. doi: 10.1016/s0140-6736(17)33050-7 [published Online First: 2018/03/06]
3. Hsiao A, Hall AH, Mogasale V, et al. The health economics of cholera: A systematic review. *Vaccine* 2018;36(30):4404-24. doi: 10.1016/j.vaccine.2018.05.120 [published Online First: 2018/06/17]
4. Hsiao A, Desai SN, Mogasale V, et al. Lessons learnt from 12 oral cholera vaccine campaigns in resource-poor settings. *Bulletin of the World Health Organization* 2017;95(4):303-12. doi: 10.2471/blt.16.175166 [published Online First: 2017/05/10]
5. Pezzoli L. Global oral cholera vaccine use, 2013-2018. *Vaccine* 2019 doi: 10.1016/j.vaccine.2019.08.086 [published Online First: 2019/09/15]
6. Msyamboza KP, Kagoli M, M'Bang'ombe M, et al. Cholera outbreaks in Malawi in 1998-2012: social and cultural challenges in prevention and control. *Journal of infection in developing countries* 2014;8(6):720-6. doi: 10.3855/jidc.3506 [published Online First: 2014/06/12]
7. Swerdlow DL, Malenga G, Begkoyian G, et al. Epidemic cholera among refugees in Malawi, Africa: treatment and transmission. *Epidemiology and infection* 1997;118(3):207-14. [published Online First: 1997/06/01]
8. World Health Organization. Cholera Country Profile: Malawi 2010.
9. M'Bangombe M, Pezzoli L, Reeder B, et al. Oral cholera vaccine in cholera prevention and control, Malawi. *Bulletin of the World Health Organization* 2018;96(6):428-35. doi: 10.2471/blt.17.207175 [published Online First: 2018/06/16]
10. Msyamboza KP, M'Bang'ombe M, Hausi H, et al. Feasibility and acceptability of oral cholera vaccine mass vaccination campaign in response to an outbreak and floods in Malawi. *The Pan African medical journal* 2016;23:203. doi: 10.11604/pamj.2016.23.203.8346 [published Online First: 2016/06/28]
11. Sauvageot D, Saussier C, Gobeze A, et al. Oral cholera vaccine coverage in hard-to-reach fishermen communities after two mass Campaigns, Malawi, 2016. *Vaccine* 2017;35(38):5194-200. doi: 10.1016/j.vaccine.2017.07.104 [published Online First: 2017/08/15]
12. Government of Malawi. Malawi 2015 Floods Post Disaster Needs Assessment Report, March 2015.
13. World Health Organization. Ending Cholera—A Global Roadmap to 2030, 2017.
14. Ilboudo PG, Huang XX, Ngwira B, et al. Cost-of-illness of cholera to households and health facilities in rural Malawi. *PloS one* 2017;12(9):e0185041. doi: 10.1371/journal.pone.0185041 [published Online First: 2017/09/22]
15. Mukherjee P, Ghosh S, Ramamurthy T, et al. Evaluation of a rapid immunochromatographic dipstick kit for diagnosis of cholera emphasizes its outbreak utility. *Jpn J Infect Dis* 2010;63(4):234-38.

For peer review only - http://bmjopen.bmj.com/site/about/guidelines.xhtml

16. Sinha A, Sengupta S, Ghosh S, et al. Evaluation of a rapid dipstick test for identifying cholera cases during the outbreak. *The Indian journal of medical research* 2012;135(4):523-8. [published Online First: 2012/06/06]

17. World Health Organization, Department of Immunization Vaccines and Biologicals. Guidelines for estimating the economic burden of diarrhoeal disease, with focus on assessing the costs of rotavirus diarrhoea. Geneva, Switzerland, July 2005.

18. Poulos C, Riewpaiboon A, Stewart JF, et al. Costs of illness due to endemic cholera. *Epidemiology and infection* 2012;140(3):500-09.

19. Lee J-S, Mogasale V, Lim JK, et al. A multi-country study of the economic burden of dengue fever based on patient-specific field surveys in Burkina Faso, Kenya, and Cambodia. *PLoS neglected tropical diseases* 2019;13(2):e0007164. doi: 10.1371/journal.pntd.0007164

20. US Social Security Office of Retirement and Disability Policy. Social Security Programs Throughout the World: Africa, 2019 n.d. [Available from: Available at https://www.ssa.gov/policy/docs/progdesc/ssptw/2018-2019/africa/malawi.html.

21. Chima RI, Goodman CA, Mills A. The economic impact of malaria in Africa: a critical review of the evidence. *Health policy (Amsterdam, Netherlands)* 2003;63(1):17-36. doi: 10.1016/s0168-8510(02)00036-2 [published Online First: 2002/12/07]

22. Muheya G. Malawi govt increases minimum wage for all workers from K787.70 to K962.00 per day. *Nyasa Times* 2017.

23. Morgan W, A L, Hutubessy R, et al. Cost of treatment of cholera in rural Malawi Health facilities. (Under Preparation)

24. World Health Organization. The Abuja Declaration: Ten Years On 2011 [Available from: Available at http://www.who.int/healthsystems/publications/abuja_declaration/en/.

25. Ministry of Health. Malawi Health Sector Strategic Plan, 2011-2016. 2011

For peer review only - http://bmjopen.bmj.com/site/about/guidelines.xhtml

TABLES

Table 1. Demographic and medical characteristics of cholera cases in Nsanje District, Malawi, 2016–2020

	Outpatient n (%)	Inpatient n (%)	Total n (%)
**Total**	31 (78)	9 (22)	40 (100)
**Sex**			
Male	9 (29)	6 (67)	15 (38)
Female	17 (55)	3 (33)	20 (50)
Unknown/Missing	5 (16)	0 (0)	5 (13)
**Age**			
<5 years	10 (32)	4 (44)	14 (35)
≥5 years to <18 years	2 (6)	1 (11)	3 (8)
≥18 years to <35 years	9 (29)	0 (0)	9 (23)
≥35 years	3 (10)	3 (33)	6 (15)
Unknown/Missing	7 (23)	1 (11)	8 (20)
**Level of education**			
Never went to school	13 (42)	3 (33)	16 (40)
Preschool	2 (6)	0 (0)	2 (5)
Completed primary	10 (32)	3 (33)	13 (33)
Completed secondary	2 (6)	0 (0)	2 (5)
Unknown/Missing	4 (13)	3 (33)	7 (18)
**Employment status**			
Works for a wage	5 (16)	0 (0)	5 (13)
Does not work for a wage	26 (84)	9 (100)	35 (88)
**Average inpatient stay, days (min-max)**	—	2 (1–4)	—
**Average number of days prior to seeking medical care (min–max)**	1.4 (0–4)	2.8 (1–8)	1.7 (0–8)
**Average number of days sick with cholera**	5 (2–17)	8 (2–19)	6 (2–19)
**Method of transportation for patients to health center**			
Walked	9 (29)	1 (11)	10 (25)
Biked	12 (39)	5 (56)	17 (43)
motorcycle	3 (10)	1 (11)	4 (10)
Minibus	5 (16)	1 (11)	6 (15)
private car	1 (3)	0 (0)	1 (3)
hired canoe	1 (3)	0 (0)	1 (3)
ambulance (from MOH)	0 (0)	1 (11)	1 (3)
**Vaccination status (OCV in 2015)**			
Not vaccinated	16 (52)	5 (56)	21 (53)

One dose	5	(16)	1	(11)	6	(15)
Two doses	6	(19)	0	(0)	6	(15)
Unknown/Missing	4	(13)	3	(33)	7	(18)
**Cholera confirmation**						
Rapid diagnostic test	5	(56)	4	(44)	9	(22)
Lab culture confirmation	25	(93)	2	(7)	27	(68)
Clinically-identified	1	(25)	3	(75)	4	(10)

* Sum of percentages may exceed 100 due to rounding.

For peer review only

Table 2. Mean direct and indirect costs for cholera cases in Nsanje District, Malawi, 2016–2020*

	Inpatient (n=9) MWK (min, max)	2019 USD (min, max)	Outpatient (n=31) MWK (min, max)	2019 USD (min, max)	Total costs (n=40) MWK	2019 USD	% of total
**Direct, medical costs**	**89**	**0.12**	**193**	**0.26**	**170**	**0.23**	**2.6%**
Drugs and consumables[1]	89 (0–800)	0.12 (0–1.08)	193 (0–6,000)	0.26 (0–8.12)	170	0.23	2.6%
**Direct, non-medical costs**	**8,528**	**11.55**	**2,258**	**3.06**	**3,669**	**4.97**	**55.6%**
Patient food and/or water	411 (0–1,100)	0.56 (0–1.49)	509 (0–5,000)	0.69 (0–6.77)	487	0.66	7.4%
Patient transportation (roundtrip)	1,889 (0–4000)	2.56 (0–5.41)	566 (0–8,000)	0.77 (10.83)	864	1.17	13.1%
Caregiver food and/or water	1,483 (0–1,600)	2.01 (0–2.17)	731 (0–6,000)	0.99 (0–8.12)	900	1.22	13.6%
Caregiver transportation (roundtrip)	4,745 (0–11,000)	6.42 (0–14.89)	452 (0–6,000)	0.61 (0–8.12)	1,418	1.92	21.5%
**Indirect (productivity losses)[2]**	**422**	**0.57**	**3,447**	**4.66**	**2,767**	**3.74**	**41.8%**
Patient	0 (0–0)	0 (0–0)	823 (0–7,500)	1.11 (0–10.15)	638	0.86	9.6%
Caregivers	422 (0–2,000)	0.57 (0–13.53)	2,624 (0–15,000)	3.55 (0–20.30)	2,129	2.88	32.2%
**Total costs**	**9,039**	**12.24**	**5,898**	**7.98**	**6,606**	**8.94**	**100.0%**

* All costs are averaged over the total number of patients in each category (i.e., patients or caregivers with zero costs are not excluded in the average)

[1] Only two patients (one inpatient, one outpatient) had any DMCs, but costs are averaged over the total number of patients

[2] Productivity losses are averaged over the total number of patients in each category (not averaged over the total number of caregivers); only two caregivers (of two inpatient patients) had any reported wage losses, but wages are averaged over the total number of inpatients

For peer review only - http://bmjopen.bmj.com/site/about/guidelines.xhtml

Table 3. Scenario analyses of mean indirect costs for cholera cases with reported wage losses in Nsanje District, Malawi, 2016–2020

	Scenario 1: Self-reported wages only		% of total	Scenario 2: Self-reported + minimum wage for patients aged 15–50 years		% of total	Scenario 3: Self-reported + minimum wage for patients aged >5 years		% of total
	MWK[3] (min, max)	2019 USD (min, max)		MWK (min, max)	2019 USD (min, max)		MWK (min, max)	2019 USD (min, max)	
**Indirect, productivity losses**									
Patients[1]	5,100 (3,000–7,500)	6.90 (4.06–10.15)	47%	4,450 (1,924–7,500)	6.02 (2.60–10.15)	58%	5,008 (1,443–11,544)	6.78 (1.95–15.62)	67%
Caregivers[2]	5,543 (600–15,000)	5.76 (0.81–20.30)	41%	3,239 (600–15,000)	3.37 (0.81–20.30)	32%	3,239 (600–15,000)	3.37 (0.81–20.30)	33%
**Total indirect costs**	**10,643**	**12.66**		**7,689**	**9.39**		**8,247**	**10.15**	

[1] All 5 patients who worked for a wage were outpatient; the denominator for each scenario was as follows: n=5 for scenario 1, n=12 for scenario 2, and n=17 for scenario 3
[2] The denominator for each scenario was as follows: n=15 for scenario 1 and n=45 for scenario 2
[3]MWK= Malawian kwacha

For peer review only - http://bmjopen.bmj.com/site/about/guidelines.xhtml

Table 4. Financial and food insecurity of cholera cases in Nsanje District, Malawi, 2016–2020

	Outpatient (n=31) n (%)		Inpatient (n=9) n (%)		Total (n=40) n (%)	
**Borrowing money to pay for health care services**						
Did not borrow money	17	(55)	3	(33)	20	(50)
Borrowed money from a family member	6	(19)	0	(0)	6	(15)
Borrowed money from a friend	4	(13)	5	(56)	9	(23)
Borrowed money from a cooperative	0	(0)	1	(11)	1	(3)
Borrowed money from a neighbor	1	(3)	0	(0)	1	(3)
Borrowed money from the village chief	1	(3)	0	(0)	1	(3)
Borrowed money from boss/employer	1	(3)	0	(0)	1	(3)
Borrowed money from church	1	(3)	0	(0)	1	(3)
**Sold items to pay for health care services**						
Did not sell anything	29	(94)	4	(44)	33	(83)
Rented out rice and maize plots	0	(0)	4	(44)	4	(10)
Sold radio	1	(3)	0	(0)	1	(3)
Sold livestock	1	(3)	1	(11)	2	(5)
**Frequency of household going without food in the past year**						
Never	4	(13)	1	(11)	5	(13)
Once every 3 months	7	(23)	1	(11)	8	(20)
Once per month	3	(10)	2	(22)	5	(13)
Twice per month	2	(6)	1	(11)	3	(8)
Once per week	8	(26)	1	(11)	9	(23)
Almost every day	3	(10)	0	(0)	3	(8)
Unknown/Missing	4	(13)	3	(33)	7	(18)

only

For peer review only - http://bmjopen.bmj.com/site/about/guidelines.xhtml

BMJ Open

The economic impact of cholera in households in rural southern Malawi: a prospective study

Journal:	*BMJ Open*
Manuscript ID	bmjopen-2021-052337.R1
Article Type:	Original research
Date Submitted by the Author:	04-Feb-2022
Complete List of Authors:	Hsiao, Amber; Technische Universität Berlin Fakultät Wirtschaft und Management, Department of Health Care Management<br>Ramani, Enusa; International Vaccine Institute, Policy and Economic Research ; Technische Universität Berlin Fakultät Wirtschaft und Management, Department of Health Care Management<br>Seo, Hye-jin; International Vaccine Institute, Epidemiology, Public Health, Impact<br>Pak, GiDeok; International Vaccine Institute, Biostatistics & Data Management<br>Vuntade, Dan ; University of Malawi the Polytechnic, Department of Environmental Health<br>M'bang'ombe, Maurice; Ministry of Health Malawi<br>Ngwira, Bagrey; University of Malawi the Polytechnic, Department of Environmental Health<br>Quentin, Wilm; Technische Universität Berlin Fakultät Wirtschaft und Management, Department of Health Care Management<br>Marks, Florian ; International Vaccine Institute, Epidemiology, Public Health, Impact; Cambridge Institute of Therapeutic Immunology and Infectious Disease, Cambridge School of Clinical Medicine, Cambridge Biomedical Campus<br>Mogasale, Vittal; International Vaccine Institute, Policy and Economic Research
**Primary Subject Heading**:	Global health
Secondary Subject Heading:	Health economics, Infectious diseases, Health policy
Keywords:	Public health < INFECTIOUS DISEASES, HEALTH ECONOMICS, Health policy < HEALTH SERVICES ADMINISTRATION & MANAGEMENT, Economics < TROPICAL MEDICINE

SCHOLARONE™
Manuscripts

I, the Submitting Author has the right to grant and does grant on behalf of all authors of the Work (as defined in the below author licence), an exclusive licence and/or a non-exclusive licence for contributions from authors who are: i) UK Crown employees; ii) where BMJ has agreed a CC-BY licence shall apply, and/or iii) in accordance with the terms applicable for US Federal Government officers or employees acting as part of their official duties; on a worldwide, perpetual, irrevocable, royalty-free basis to BMJ Publishing Group Ltd ("BMJ") its licensees and where the relevant Journal is co-owned by BMJ to the co-owners of the Journal, to publish the Work in this journal and any other BMJ products and to exploit all rights, as set out in our licence.

The Submitting Author accepts and understands that any supply made under these terms is made by BMJ to the Submitting Author unless you are acting as an employee on behalf of your employer or a postgraduate student of an affiliated institution which is paying any applicable article publishing charge ("APC") for Open Access articles. Where the Submitting Author wishes to make the Work available on an Open Access basis (and intends to pay the relevant APC), the terms of reuse of such Open Access shall be governed by a Creative Commons licence – details of these licences and which Creative Commons licence will apply to this Work are set out in our licence referred to above.

Other than as permitted in any relevant BMJ Author's Self Archiving Policies, I confirm this Work has not been accepted for publication elsewhere, is not being considered for publication elsewhere and does not duplicate material already published. I confirm all authors consent to publication of this Work and authorise the granting of this licence.

For peer review only

The economic impact of cholera in households in rural southern Malawi: a prospective study

Amber Hsiao,[1] Enusa Ramani,[1,2] Hye Jin Seo,[2] Gi Deok Pak[2], Dan Vuntade[3], Maurice M'bang'ombe,[4] Bagrey Ngwira,[3] Wilm Quentin,[1] Florian Marks,[2,5,6] Vittal Mogasale[2*]

[1] Technische Universität Berlin, Department of Health Care Management, Berlin, Germany

[2] International Vaccine Institute, Seoul, Republic of Korea

[3] University of Malawi, Department of Community Health, Blantyre, Malawi

[4] Ministry of Health Malawi, Lilongwe, Malawi

[5] Cambridge Institute of Therapeutic Immunology and Infectious Disease, University of Cambridge School of Clinical Medicine, Cambridge Biomedical Campus, Cambridge, UK, CB20AW

[6] University of Antananarivo, Antananarivo, Madagascar

***Corresponding Author:**

Vittal Mogasale, MBBS, MPH, PhD

International Vaccine Institute, Policy & Economic Research Department,

SNU Research Park, 1 Gwanak-ro, Gwanak-gu Seoul 08826. The Republic of Korea.

T +82 2 881 1379; F + 82 2 872 2803

Email: vmogasale@gmail.com; vmogasale@ivi.int

Word count (excluding title page, abstract, references, tables/figures)

For peer review only - http://bmjopen.bmj.com/site/about/guidelines.xhtml

ABSTRACT

Introduction. Cholera remains a significant contributor to diarrheal illness, especially in sub-Saharan Africa. Few studies have estimated the cost of illness (COI) of cholera in Malawi, a cholera-endemic country. The present study estimated the COI of cholera in Nsanje, southern Malawi, as part of the Cholera Surveillance in Malawi (CSIMA) program following a mass cholera vaccination campaign in 2015.

Methods: Patients ≥12 months of age who were recruited as part of CSIMA were invited to participate in the COI survey. The COI tool captured household components of economic burden, including direct medical and non-medical costs, and indirect lost productivity costs.

Results: Between April 2016 to March 2020, 40 cholera cases were enrolled in the study, all of whom participated in the COI survey. Only two patients had any direct medical costs and five patients reported lost wages due to illness. The COI per patient was $14.34 (in 2020 USD), more than half of which was from direct non-medical costs from food, water, and transportation to the health center.

Conclusion: For the majority of Malawians who struggle to subsist on less than $2 a day, the COI of cholera represents a significant cost burden to families. While cholera treatment is provided for free in government-run health centers, additional investments in cholera control and prevention at the community level and financial support beyond direct medical costs may be necessary to alleviate the economic burden of cholera on households in southern Malawi.

For peer review only - http://bmjopen.bmj.com/site/about/guidelines.xhtml

Strengths and limitations of this study

- We recruited cholera-confirmed patients at the time they first sought care and interviewed them regarding costs incurred due to illness within 7 days following their initial health facility visit, which limited recall bias.
- The sample size was relatively small, which limits the generalizability of the findings.
- The cost of illness estimate is likely to be an underestimate as it only includes out-of-pocket expenditures of patients who sought care.

For peer review only

INTRODUCTION

Cholera is an infection of the intestine caused by the bacterium *Vibrio cholerae* that leads to acute, rapidly-dehydrating diarrheal disease. Annually, an estimated 2.86 million cases of cholera occur in cholera endemic countries that result in 95,000 deaths.[1] A recent study estimated that cholera is especially pervasive throughout sub-Saharan Africa (sSA) where the human and economic burden of cholera is greatest, with more than 200 million people living in areas where cholera is present. Of these 200 million, 87 million live in areas with high incidence, defined as >1 case per 1,000 people per year.[2]

While improvements to water, sanitation, and hygiene (WASH) are the best long-term solution for preventing more diarrheal diseases, these measures remain unavailable to large proportions of people in sub-Saharan Africa, especially among those living in flood-prone rural areas. The availability of low-cost inactivated oral cholera vaccines (OCV) since 2011 has significantly changed the landscape and provided a cost-effective short-to-middle-term approach for cholera prevention and control.[3] A number of mass vaccination campaigns—many of which have taken place in sSA—have demonstrated that it can be successfully administered in a variety of settings, including high-risk settings to prevent an impending outbreak, or to quickly halt the transmission of an ongoing outbreak.[4 5]

In Malawi specifically, cholera has been reported since the 1970s and is endemic to the country.[6-8] The extent of the cholera burden in the country has not been well documented due to the lack of sustainable diagnostic and surveillance capacity.

For peer review only - http://bmjopen.bmj.com/site/about/guidelines.xhtml

One of the worst cholera outbreaks to date occurred in January 2015 following the nation's worst floods in history, which affected more than 1.1 million people in the Southern Region and displaced at least 230,000 people from their homes.[9] Within a month of the flood, cholera cases were being reported in Nsanje and Chikwawa, two of the worst-hit districts in southern Malawi. The flooding led to a multi-stakeholder response to coordinate a reactive OCV mass vaccination campaign in the region.[10] Due to the lack of diagnostic capacity, cholera cases were being diagnosed based on clinical presentation as per the World Health Organization (WHO) and national guidelines.[11]

The OCV mass vaccination campaign was then implemented primarily in Nsanje, which was identified as having the highest burden of cholera based on prior years' data provided by the Malawian Ministry of Health (MOH). From March 30, 2015, to April 23, 2015, the International Vaccine Institute (IVI), MOH, WHO, and John Snow, Inc. coordinated a reactive mass vaccination campaign that targeted 160,000 people (320,000 total doses of the OCV Shanchol® (Shantha Biotechnics Pvt., Ltd., India) over 1 year of age. Those living in high-risk areas of Nsanje—namely, those living in internally displaced camps and surrounding villages—were the primary targets.[10]

In the course of a 4-year effort to strengthen cholera surveillance capacity and infrastructure in Nsanje following the mass vaccination campaign, we investigated the cost-of-illness (COI) of cholera for patients who sought care. Only one study to date has reported on the cost-of-illness from cholera in southeastern Malawi (Lake Chilwa in the districts of Machinga and Zomba).[12]

For peer review only - http://bmjopen.bmj.com/site/about/guidelines.xhtml

The objective of this study is to provide additional field data on the economic burden of cholera in Malawi from a household perspective.

METHODS

As part of the Cholera Surveillance in Malawi (CSIMA) program, cholera surveillance was conducted across 22 Nsanje (population >292,000) and 18 Chikwawa (population >533,000) healthcare facilities from April 5, 2016, to March 31, 2020. IVI staff trained health surveillance assistants (HSAs) and health workers at all Nsanje and Chikwawa facilities to identify patients who sought care for acute watery diarrhea. HSAs are community health facilitators that live in the area served by the local healthcare facility who are involved in activities such as WASH and general health promotion.

Patients who met the inclusion criteria (who were eligible to receive OCV based being at least 12 months of age at the start of the vaccination campaign, and presented to an Nsanje health facility with acute watery diarrhea indicative of cholera as defined by the WHO[11]) were invited to participate in the diarrheal surveillance study. Participants who provided informed written consent provided a stool specimen to perform a rapid diagnostic test (RDT) for cholera (Crystal® VC RDT; Arkray Healthcare Pvt., India; previously Span Diagnostics, Surat, India) and/or a stool sample for culture confirmation at the national reference laboratory in Lilongwe. The use of RDTs has been accepted for use by the Malawi MOH, and there is documented experience with this test kit in other settings.[13, 14] While the use of the RDT is not intended to be a substitute for stool culture, the use of the kit in this setting ensured that one would not miss any

For peer review only - http://bmjopen.bmj.com/site/about/guidelines.xhtml

potential cholera cases during the early surveillance period while logistics for stool transportation and culture were being set-up.

Between April 6, 2016, and December 12, 2017, culture-confirmed and RDT-positive cholera cases were recruited for the overall study, as well as the COI survey component. However, due to low recruitment numbers of RDT- and/or culture-confirmed cholera cases, in part due to limited availability of RDT kits and stool collection logistical challenges, the inclusion criteria were expanded from December 12, 2017, through March 31, 2020, to include clinically-diagnosed cholera cases in an outbreak situation to avoid missing potential cases.

While cholera vaccines were administered to residents in both Nsanje and Chikwawa, the majority of doses were administered to residents of Nsanje. Thus, only patients recruited from Nsanje health centers were invited for the COI portion of the study. The COI from a household perspective was assessed for patients who provided written informed consent (regardless of vaccination status). The COI tool was developed based on a tool that IVI had previously used for other COI studies, with further refinements using the guidelines for cost collection in cholera studies provided by the WHO.[15, 16] The survey tool captured household components of economic burden, divided further into direct medical costs (DMC), direct non-medical costs (DNMC), and indirect costs (IC) (as done in the previous studies[17]), with the payer being the patients themselves or caregivers/helpers of the patient (e.g., relatives). Experienced interviewers were trained on how to administer the survey and all survey responses were validated by the University of Malawi Polytechnic and MOH collaborators to ensure they were appropriate for the local context.

For peer review only - http://bmjopen.bmj.com/site/about/guidelines.xhtml

A series of a maximum of three interviews were planned for the COI assessment, with day 0 (i.e., day of diagnosis) being the day of the patient's health facility visit: at day 0–7, day 13–15 (beyond the mean duration of an episode of cholera illness), and day 20–22. To minimize recall bias, the first interview took place as soon as cholera was confirmed either by a test or a clinician, with a maximum of 7 days following the first healthcare facility visit. If a patient preferred to be interviewed while at the healthcare facility, the trained interviewer conducted the first COI interview at the time of the initial outpatient or inpatient visit. Patients who were not feeling well enough were contacted by an HSA to schedule a future interview time. Trained interviewers would then visit the patient's home at the scheduled time to conduct the first interview. At the end of each COI assessment, patients were asked whether they felt they had fully recovered from cholera. If the patient reported still being ill, the next interview was scheduled; otherwise, no additional interviews were conducted.

At each of the interviews, patients (or parent/guardian) were asked how much money they spent during each of their visits to a healthcare facility or other treatment locations (e.g., a traditional healer) since becoming ill, including any visits that may have occurred prior to the study enrollment date. DMCs included out-of-pocket payments for services, prescription drugs, and any other health facility fees. DNMCs included transportation and food expenses incurred by the patient and/or caregivers who went to the health facility with the patient.

Finally, IC accounted for the patient's and/or caregivers' productivity losses incurred due to lost wages. We calculated productivity losses using three scenarios based upon (1) self-reported wage losses, (2) minimum wage per day for all workers between 14–50 years of age (the legal

For peer review only

For peer review only - http://bmjopen.bmj.com/site/about/guidelines.xhtml

minimum and old-age pension eligibility age[18]) who did not report wage losses, and (3) minimum wage per day for all patients >5 years of age who did not report wage losses, given evidence from Africa that children are often involved in economic activities that add to a family's income.[19]

In all scenarios, if patients and/or caregivers reported wage losses, these were calculated based on self-reported daily lost productivity (half or full days of work lost due to taking care of the ill patient) and daily wages. If no wage loss was reported, we applied the minimum wage of 1,346.16 Malawian kwacha (MWK) per day ($1.83 USD/day as of January 1, 2020) to account for the monetary value of productivity losses.[20] Productivity losses were not calculated for caregivers who indicated that they did not cut back on their usual activities had they not been caring for the patient.

Costs were collected in Malawian kwacha (MWK), inflated to 2020 MWK using World Bank inflation data,[21] and converted to 2020 US$ (736.5803 MWK=$1 US on March 31, 2020).[22] All analyses were conducted in Stata/IC 13.1 (College Station, TX) and Microsoft Excel.

Ethics approval

This study was approved by the National Health Sciences Research Committee (NHSRC) of the Ministry of Health in Malawi (protocol #16/2/1547; approval #1547) on March 29, 2016. IVI's Institutional Review Board (IRB) approved the study on April 6, 2016. All participants (or a parent/guardian) provided written informed consent prior to interviews. The IVI IRB and the NHSRC approved the modification to expand to inclusion of clinically-diagnosed cases on May

For peer review only - http://bmjopen.bmj.com/site/about/guidelines.xhtml

31, 2017, and July 31, 2017, respectively. Training of all HSAs on the modification occurred on December 12 and 13, 2017, and the data collection continued until March 31, 2020.

Patient and Public Involvement

Patients including their caretakers were involved in this study as participants in the cost of illness interviews. Cost related to cholera illness were collected from the participants after obtaining informed consent. The public was not involved in this study. Patients and the public were not involved in study development, defining the research question and study design, recruitment, and the conduction of the study. Early findings of the study were disseminated through a workshop attended by country policymakers, health managers, and health facility staff.

RESULTS

During our surveillance period, 2,201 individuals met the diarrheal illness criteria for stool collection and testing for cholera in Nsanje. Of these patients, 40 (1.8%) were identified as cholera cases and were recruited for the surveillance study. On the day of their health facility visit, all 40 patients also consented for COI interview. Table 1 summarizes selected demographic characteristics, as well as OCV vaccination statuses, of all cases.

Of the 40 cases, 31 (78%) were seen in an outpatient setting. Duration of illness was 5 days (min–max: 2–17 days) in the outpatient setting and 8 days (min–max: 2–19 days) in the inpatient setting. Most patients (n=34) had fully recovered by the time the first interview occurred (scheduled within 7 days of recruitment day 0); 6 patients had a second interview (at day 13–15) and none required a third interview. A higher proportion of inpatient cases were males, whereas outpatient cases were majority female. Of the 40 cases, 4 were identified clinically, 9 using RDT,

For peer review only - http://bmjopen.bmj.com/site/about/guidelines.xhtml

and 27 using culture confirmation. A larger proportion of children were seen in the inpatient setting, compared with outpatient.

The direct and indirect cost of cholera per patient was $14.34 when averaged across all patients, with the largest proportion of costs (57.9%) being from direct non-medical costs (food, water, and transportation) (Table 2). Direct medical costs for drugs and consumables were zero for nearly all patients; only 2 patients seen in the inpatient setting had any drug costs of $11.17 (8,229 MWK) and $1.19 (875 MWK) each, both of whom were seen at a private Christian Health Association of Malawi (CHAM) facility; these two patient costs increased the overall average across the cohort to $0.31.

Patient and caregiver food costs were $0.95 and $1.61 on average, respectively. Roundtrip transportation costs were relatively lower for patients at $1.84, compared to caregiver transportation costs at $3.91. Transportation time on average per patient was 112 minutes roundtrip (min, max: 10, 360).

We also performed various scenario analyses for indirect costs (IC) (Table 3) where costs were only averaged across patients and caregivers with non-zero productivity losses in each scenario. Eight patients were excluded from wage loss calculations due to missing age or birthdate. Thirty-four patients had at least one caregiver provide care during their illness, with a total of 53 caregivers across all patients. Caregivers only reported whether they were children or adults; (i.e., exact ages or birthdates were not ascertained); all caregivers reported being adults who we assumed were of working age.

For peer review only - http://bmjopen.bmj.com/site/about/guidelines.xhtml

In scenario 1, productivity losses (lost wages) due to being sick for the duration of illness were $10.60 on average (n=5) for patients who reported wage losses, though one patient reported receiving paid sick leave for this time ($10.89 USD), which would reduce the average to $10.53 (n=4). Only 15 caregivers (who cared for 8 patients) reported $6.36 on average in lost wages due to missing work. In scenario 2 where the minimum wage was assumed for all patients (n=11) and caregivers (n=45) of working age, productivity losses were $8.83 for patients and $3.93 for caregivers on average. In scenario 3, where the minimum wage was assumed for all patients >5 years of age (n=19), productivity losses were $9.78 for patients on average. In this scenario analysis, there were 2 patients aged 5–14 years ($14.16 on average) and 5 patients aged ≥60 years ($8.59 on average). Caregiver costs in scenarios 2 and 3 were the same ($3.94) since all caregivers were adults.

Half of all patients (n=20), and two-thirds of inpatients (n=6), reported having to borrow money from someone to pay for health care, food, or transportation as a result of having cholera (Table 4), but most did not have to sell personal belongings to pay for care. Food insecurity was an issue for nearly all of the patients (n=35).

DISCUSSION

Our study estimated patient and caregiver costs associated with seeking healthcare for the treatment of severe diarrheal illness diagnosed as cholera in Nsanje. We had few diarrheal cases overall. The cost of cholera illness per patient was $14.34, with the largest proportion of costs being from direct non-medical costs (food, water, and transportation) ($8.30), followed by

For peer review only - http://bmjopen.bmj.com/site/about/guidelines.xhtml

indirect productivity losses ($5.73) in our primary analysis where costs were averaged across all patients in our cohort.

The majority of our patients were not admitted during their visit to the health center. Patients who sought care in the outpatient setting were symptomatic an average of 1.4 days prior to seeking care. Patients who were admitted were sick for an average of 2.8 days prior to seeking care and had an inpatient stay of 2 days. Our patient population was slightly skewed toward females and of those whose age was reported, most patients were adults aged 18 and older. The majority of patients were not vaccinated during the OCV campaign in 2015.

Overall, only two cholera patients had any DMCs; one was seen in the outpatient setting (875 MWK, or $1.19) and the other was admitted to a private CHAM facility (8,229 MWK, or $11.17). This finding was expected, as cholera treatment is provided free of charge in Malawi. On average, patients and their caregivers spent $8.61 in direct costs ($0.31 in medical costs and $8.30 in non-medical costs, including those with zero costs), almost all of which was due to food and/or water and transportation costs. In our scenario analysis when only those with self-reported wages are considered, only 5 cholera patients (all outpatient cases) reported productivity losses of $8.83 on average, and 15 caregivers of patients reported wage losses, averaging $3.93. In our scenario analysis where we applied the minimum wage to all patients and caregivers who theoretically could be contributing to economic activities (i.e., household work), indirect productivity losses were as high as $9.78 per patient and $3.94 per caregiver. This latter scenario may overestimate the number of individuals who experience productivity losses and the actual

For peer review only - http://bmjopen.bmj.com/site/about/guidelines.xhtml

economic impact, though the average productivity losses ($13.72) were lower than in the self-reported wage scenario ($16.96).

The overall costs found in our study are similar to or substantially less than one study previously reported in rural Malawi,[12] though, since only two patients had any drug and consumable costs in our population, they are difficult to compare. Ilboudo et al. also reported a much larger proportion of hospitalized cases: their study sampled 100 cases (which estimated 9% of the overall caseload from the cholera outbreak), 90 of whom were hospitalized, whereas we only had 7 hospitalized cases (only one of whom reported drugs and consumables costs). Transportation costs in our study were also lower for both patients and caregivers compared with their study, which may be attributable to differences in transportation methods and travel distances to health centers.

The largest differences in costs between our study and Ilboudo et al. were in food and lost productivity costs, likely also driven by the fact that their study had more hospitalized cases that required more expenses over several days. Their reported indirect costs of patient's productivity were a larger portion of overall costs (35.1% compared to our 9.2%), which may be attributed to their study cohort having longer inpatient stays. The caregiver lost productivity costs as a proportion of overall costs were lower in their study (23.5% compared to our 30.7%). Differences in productivity costs may be attributed to several reasons, such as differences in wages in Nsanje versus Machinga and Zomba districts, unemployment rates, and other socioeconomic factors. The differences may also be attributed to the severity of illness, potentially attenuated by vaccination. Ilboudo et al. assessed costs of illness from patients who

For peer review only - http://bmjopen.bmj.com/site/about/guidelines.xhtml

had cholera in the ±1 week prior to and after the OCV vaccination campaign that was underway (which followed the cholera outbreak). Thus, the proximity in timing to the outbreak may have meant that few individuals had any immunity to cholera and may have experienced more severe symptoms, whereas in our population, OCV coverage and immunity was likely higher. Given the relatively small sample size in both studies, however, more data is needed from the region to understand why these costs differed more than other cost components.

We also assessed financial and food insecurity for cases. Half of our cases had to borrow money from someone to pay for costs associated with their cholera illness and treatment, which is similar to the proportion reported by Ilboudo et al. A large proportion of our cases also faced food insecurity at some point in the prior year.

Our study had several limitations. One limitation was the late start of cholera surveillance (April 2016) relative to the cholera outbreak (~February 2015), in response to which a vaccination campaign was conducted (March/April 2015). It is possible that some post vaccination campaign cholera cases may have occurred in the year prior to the start of cholera surveillance, particularly during the November 2015 to April 2016 rainy season; these cases would not have been included in this study. The study was also not able to collect COI data from Chikwawa residents and infants <12 months, which may have provided different cost estimates. Given the small number of cholera cases, even after expanding the case criteria to clinically-diagnosed cases (of which there were only 4), it may not be representative of the general Nsanje population, particularly those who were sick and did not seek care. It is also possible that patients may have shown up for care but were undiagnosed or misdiagnosed for reasons such as diagnostic sensitivity. While we

For peer review only - http://bmjopen.bmj.com/site/about/guidelines.xhtml

330 were unable to assess any patterns or variation in the use of the three testing methods (RDT,
331 culture, or clinically-identified) by the health center, it is possible that our recruited patient
332 cohort is biased toward health centers that were better equipped to test, recruit, and treat patients.
333 For example, many of the more remote health centers in Nsanje lacked toilets or latrines for
334 proper stool collection and have limited access to electricity to ensure the viability of stool
335 samples, which may have limited the sensitivity of the stool culture result. Apart from the
336 logistical challenges of recruitment, the area also has a high number of migrants from
337 Mozambique; migrants may be less likely to seek care due to several reasons, including language
338 barriers, discrimination due to cultural differences, and fear of deportation.[23]
339
340 Health facility costs were also not included in our costing, which may be a significant portion of
341 the total costs as most patients reported $0 in direct medical costs. A recent study in rural Malawi
342 estimated that the average treatment cost to a health facility for a hospitalized cholera case was
343 $59.70 in US$ 2016 ($96.61 in US$ 2020).[12] A separate study on cholera treatment costs at the
344 facility level at this study site has estimated an economic cost of $41.86 in US$ 2018 ($50.79 in
345 US$ 2020) and a financial cost of $25.43 in US$ 2018 ($30.85 in US$ 2020).[24] Finally, there
346 was the potential for recall bias, especially for patients who may have had costs prior to the
347 initial visit to the health center from which they were recruited for the study. For costs incurred
348 from the day of enrollment onward, we attempted to minimize the potential for recall bias by
349 following up first within a week of the initial visit.
350
351 Despite the limitations, our study provides one of the few cost of illness studies of cholera illness
352 in sub-Saharan Africa from the household perspective. More studies are needed to more fully

For peer review only - http://bmjopen.bmj.com/site/about/guidelines.xhtml
For peer review only

evaluate the cholera costs of illness in Malawi. Currently, spending on health accounts for less than 10% of country revenues—below the 15% target set by the Abuja Declaration to which Malawi is a signatory.[25] Health care financing in Malawi is also unpredictable and unsustainable given the country's reliance on development partners' contributions that account for nearly two-thirds of total health expenditure.[26] Our study supports the need for further spending in the health sector; for example, building and staffing additional health centers that reduce the amount of time patients have to travel could reduce the cost burden on patients.

While the study was not designed to assess vaccine effectiveness, our surveillance efforts may also be a positive indication that the cholera vaccine may be associated with a reduction in the burden of cholera in the middle-term, as most of our cases were unvaccinated (while approximately half of the Nsanje population received the cholera vaccine). Since this study was conducted, in recognition of the high incidence of cholera and the need for additional tools to address the burden, the government of Malawi integrated OCVs into its national cholera control plan in 2017,[27] and there have been several OCV campaigns implemented since.[10, 28] Alternatively, given that cholera often occurs cyclically, it is possible that the time period during which surveillance occurred was a low or no cholera transmission interval. Ultimately, investing more heavily in WASH infrastructure would likely be the a most effective, though more costly, solution in the long-run to avert cholera.

In a country where the gross domestic product per capita per day is less than $2 ($625 annually in 2020 USD), a family who has to spend $14 on average for cholera illness will very likely have to make financial sacrifices to access treatment. Even though the direct medical costs associated with cholera care are low, the patient (who is willing and able to go to the health center) must

For peer review only - http://bmjopen.bmj.com/site/about/guidelines.xhtml

bear a high burden of direct non-medical and indirect lost productivity costs. For those who do not have the means of even going to a health facility to seek care, the consequences are likely even more severe; for example, the death of a family member whose wages supported the family has significant long-term impacts beyond a single cholera episode.

In conclusion, we find that households face a significant cost burden when one falls sick with cholera. These costs are significantly high to warrant additional investments in the health system to avert future cases of cholera.

AUTHORS CONTRIBUTIONS

AH and VM conceptualized the study, led the protocol development, field training, and survey implementation in collaboration with HJS, GDP, DV, MM, BN, FM, & ER. Data collection was supervised by DV, MM, and BN, and monitored by GDP, AH, VM, ER, and HJS. Data analysis and interpretation were done by AH supported by VM and WQ. The manuscript was written by AH, supported by VM and WQ. All authors have reviewed, edited, and approved the final manuscript.

COMPETING INTERESTS

None declared.

DATA SHARING

For peer review only - http://bmjopen.bmj.com/site/about/guidelines.xhtml

All data relevant to the study are included in the article or uploaded as supplementary information

FUNDING

This research was conducted by International Vaccine Institute and funded by Bill & Melinda Gates Foundation [OPP38590] and the UK Foreign, Commonwealth and Development Office and Wellcome [214662/Z/18/Z]. The International Vaccine Institute acknowledges its donors including the Republic of Korea, Swedish International Development Cooperation Agency and Republic of India.

For peer review only

REFERENCES

1. Ali M, Nelson AR, Lopez AL, et al. Updated global burden of cholera in endemic countries. *PLoS neglected tropical diseases* 2015;9(6):e0003832. doi: 10.1371/journal.pntd.0003832 [published Online First: 2015/06/05]
2. Lessler J, Moore SM, Luquero FJ, et al. Mapping the burden of cholera in sub-Saharan Africa and implications for control: an analysis of data across geographical scales. *Lancet (London, England)* 2018;391(10133):1908-15. doi: 10.1016/s0140-6736(17)33050-7 [published Online First: 2018/03/06]
3. Hsiao A, Hall AH, Mogasale V, et al. The health economics of cholera: A systematic review. *Vaccine* 2018;36(30):4404-24. doi: 10.1016/j.vaccine.2018.05.120 [published Online First: 2018/06/17]
4. Hsiao A, Desai SN, Mogasale V, et al. Lessons learnt from 12 oral cholera vaccine campaigns in resource-poor settings. *Bulletin of the World Health Organization* 2017;95(4):303-12. doi: 10.2471/blt.16.175166 [published Online First: 2017/05/10]
5. Pezzoli L. Global oral cholera vaccine use, 2013-2018. *Vaccine* 2019 doi: 10.1016/j.vaccine.2019.08.086 [published Online First: 2019/09/15]
6. Msyamboza KP, Kagoli M, M'Bang'ombe M, et al. Cholera outbreaks in Malawi in 1998-2012: social and cultural challenges in prevention and control. *Journal of infection in developing countries* 2014;8(6):720-6. doi: 10.3855/jidc.3506 [published Online First: 2014/06/12]
7. Swerdlow DL, Malenga G, Begkoyian G, et al. Epidemic cholera among refugees in Malawi, Africa: treatment and transmission. *Epidemiology and infection* 1997;118(3):207-14. [published Online First: 1997/06/01]
8. World Health Organization. Cholera Country Profile: Malawi 2010.
9. Government of Malawi. Malawi 2015 Floods Post Disaster Needs Assessment Report, March 2015.
10. Msyamboza KP, M'Bang'ombe M, Hausi H, et al. Feasibility and acceptability of oral cholera vaccine mass vaccination campaign in response to an outbreak and floods in Malawi. *The Pan African medical journal* 2016;23:203. doi: 10.11604/pamj.2016.23.203.8346 [published Online First: 2016/06/28]
11. World Health Organization. Ending Cholera—A Global Roadmap to 2030, 2017.
12. Ilboudo PG, Huang XX, Ngwira B, et al. Cost-of-illness of cholera to households and health facilities in rural Malawi. *PloS one* 2017;12(9):e0185041. doi: 10.1371/journal.pone.0185041 [published Online First: 2017/09/22]
13. Mukherjee P, Ghosh S, Ramamurthy T, et al. Evaluation of a rapid immunochromatographic dipstick kit for diagnosis of cholera emphasizes its outbreak utility. *Jpn J Infect Dis* 2010;63(4):234-38.
14. Sinha A, Sengupta S, Ghosh S, et al. Evaluation of a rapid dipstick test for identifying cholera cases during the outbreak. *The Indian journal of medical research* 2012;135(4):523-8. [published Online First: 2012/06/06]
15. World Health Organization, Department of Immunization Vaccines and Biologicals. Guidelines for estimating the economic burden of diarrhoeal disease, with focus on assessing the costs of rotavirus diarrhoea. Geneva, Switzerland, July 2005.
16. Poulos C, Riewpaiboon A, Stewart JF, et al. Costs of illness due to endemic cholera. *Epidemiology and infection* 2012;140(3):500-09.

For peer review only

For peer review only - http://bmjopen.bmj.com/site/about/guidelines.xhtml

17. Lee J-S, Mogasale V, Lim JK, et al. A multi-country study of the economic burden of dengue fever based on patient-specific field surveys in Burkina Faso, Kenya, and Cambodia. *PLoS neglected tropical diseases* 2019;13(2):e0007164. doi: 10.1371/journal.pntd.0007164
18. US Social Security Office of Retirement and Disability Policy. Social Security Programs Throughout the World: Africa, 2019 n.d. [Available from: Available at https://www.ssa.gov/policy/docs/progdesc/ssptw/2018-2019/africa/malawi.html.
19. Chima RI, Goodman CA, Mills A. The economic impact of malaria in Africa: a critical review of the evidence. *Health policy (Amsterdam, Netherlands)* 2003;63(1):17-36. doi: 10.1016/s0168-8510(02)00036-2 [published Online First: 2002/12/07]
20. WageIndicator.org; Minimum Wage – Malawi (January 1, 2020). https://wageindicator.org/salary/minimum-wage/malawi/archive/20200101 Accessed 1 February 2022.
21. World Bank. "Inflation, consumer prices (annual %) – Malawi. https://data.worldbank.org/indicator/FP.CPI.TOTL.ZG?locations=MW Accessed 31 January 2022.
22. Reserve Bank of Malawi. Exchange Rates. https://www.rbm.mw/Statistics/MajorRates Accessed 31 January 2022.
23. Legido-Quigley H, Pocock N, Tan ST, Pajin L, Suphanchaimat R, Wickramage K, McKee M, Pottie K. Healthcare is not universal if undocumented migrants are excluded. BMJ 2019; 366:l4160.
24. Morgan W, A L, Hutubessy R, et al. Cost of treatment of cholera in rural Malawi Health facilities. (Under Preparation)
25. World Health Organization. The Abuja Declaration: Ten Years On 2011 [Available from: Available at http://www.who.int/healthsystems/publications/abuja_declaration/en/.
26. Ministry of Health. Malawi Health Sector Strategic Plan II, 2017-2022. April 2017. https://extranet.who.int/countryplanningcycles/sites/default/files/planning_cycle_repository/malawi/health_sector_strategic_plan_ii_030417_smt_dps.pdf accessed 20 JAN 2022.
27. M'Bangombe M, Pezzoli L, Reeder B, et al. Oral cholera vaccine in cholera prevention and control, Malawi. *Bulletin of the World Health Organization* 2018;96(6):428-35. doi: 10.2471/blt.17.207175 [published Online First: 2018/06/16]
28. Sauvageot D, Saussier C, Gobeze A, et al. Oral cholera vaccine coverage in hard-to-reach fishermen communities after two mass Campaigns, Malawi, 2016. *Vaccine* 2017;35(38):5194-200. doi: 10.1016/j.vaccine.2017.07.104 [published Online First: 2017/08/15]

For peer review only - http://bmjopen.bmj.com/site/about/guidelines.xhtml

TABLES

Table 1. Demographic and medical characteristics of cholera cases in Nsanje District, Malawi, 2016–2020

	Outpatient n (%)	Inpatient n (%)	Total n (%)
**Total**	31 (78)	9 (22)	40 (100)
**Sex**			
Male	9 (29)	6 (67)	15 (38)
Female	17 (55)	3 (33)	20 (50)
Unknown/Missing	5 (16)	0 (0)	5 (13)
**Age**			
<5 years	10 (32)	4 (44)	14 (35)
≥5 years to <18 years	2 (6)	1 (11)	3 (8)
≥18 years to <35 years	9 (29)	0 (0)	9 (23)
≥35 years	3 (10)	3 (33)	6 (15)
Unknown/Missing	7 (23)	1 (11)	8 (20)
**Level of education**			
Never went to school	13 (42)	3 (33)	16 (40)
Preschool	2 (6)	0 (0)	2 (5)
Completed primary	10 (32)	3 (33)	13 (33)
Completed secondary	2 (6)	0 (0)	2 (5)
Unknown/Missing	4 (13)	3 (33)	7 (18)
**Employment status**			
Works for a wage	5 (16)	0 (0)	5 (13)
Does not work for a wage	26 (84)	9 (100)	35 (88)
**Average inpatient stay, days (min-max)**	—	2 (1–4)	—
**Average number of symptomatic days prior to seeking medical care (min–max)**	1.4 (0–4)	2.8 (1–8)	1.7 (0–8)
**Average number of days sick with cholera**	5 (2–17)	8 (2–19)	6 (2–19)
**Method of transportation for patients to health center**			
Walked	9 (29)	1 (11)	10 (25)
Biked	12 (39)	5 (56)	17 (43)
motorcycle	3 (10)	1 (11)	4 (10)
Minibus	5 (16)	1 (11)	6 (15)
private car	1 (3)	0 (0)	1 (3)
hired canoe	1 (3)	0 (0)	1 (3)
ambulance (from MOH)	0 (0)	1 (11)	1 (3)
**Vaccination status (OCV in 2015)**			
Not vaccinated	16 (52)	5 (56)	21 (53)

For peer review only - http://bmjopen.bmj.com/site/about/guidelines.xhtml

One dose	5	(16)	1	(11)	6	(15)
Two doses	6	(19)	0	(0)	6	(15)
Unknown/Missing	4	(13)	3	(33)	7	(18)
**Cholera confirmation**						
Rapid diagnostic test	5	(56)	4	(44)	9	(22)
Lab culture confirmation	25	(93)	2	(7)	27	(68)
Clinically-identified	1	(25)	3	(75)	4	(10)

495 * Sum of percentages may exceed 100 due to rounding.

496

For peer review only

For peer review only - http://bmjopen.bmj.com/site/about/guidelines.xhtml

Table 2. Mean direct and indirect costs (MWK and USD 2020) for cholera cases in Nsanje District, Malawi, 2016–2020* (averaged across all 40 patients)

	Inpatient (n=9)		Outpatient (n=31)		Total costs (n=40)		
	2020 MWK (min, max)	2020 USD (min, max)	2020 MWK (min, max)	2020 USD (min, max)	2020 MWK	2020 USD	% of total
**Direct, medical costs**	**97**	**0.13**	**265**	**0.36**	**228**	**0.31**	**2.2%**
Drugs and consumables[1]	97 (0–875)	0.13 (0–1.19)	265 (0–8,229)	0.36 (0–11.17)	228	0.31	2.6%
**Direct, non-medical costs**	**9,703**	**13.17**	**5,073**	**6.89**	**6,115**	**8.30**	**57.9%**
Patient food and/or water	505 (0–1,353)	0.69 (0–1.84)	753 (0–6,148)	1.02 (0–8.35)	697	0.95	6.6%
Patient transportation (roundtrip)	2,007 (0–8,750)	2.73 (0–11.88)	1,161 (0–9,836)	1.58 (0–13.35)	1,352	1.84	12.8%
Caregiver food and/or water	1,332 (0–2,734)	1.81 (0–3.71)	1,143 (0–12,686)	1.55 (0–17.22)	1,186	1.61	11.2%
Caregiver transportation (roundtrip)	5,858 (0–13,525)	7.95 (0–18.36)	2,016 (0–8,346)	2.74 (0–11.33)	2,880	3.91	27.3%
**Indirect (productivity losses)[2]**	**489**	**0.66**	**5,304**	**7.20**	**4,221**	**5.73**	**40.0%**
Patient	0 (0–0)	0 (0–0)	1,259 (0–12,519)	1.71 (0–17.00)	976	1.32	9.2%
Caregivers	489 (0–2,213)	0.66 (0–3.00)	4,045 (0–70,526)	5.49 (0–95.75)	3,245	4.41	.0.7%
**Total mean costs[3]**	**10,289**	**13.97**	**10,643**	**14.45**	**10,563**	**14.34**	**100.0%**

* All costs by category are averaged over the total number of patients in each category (i.e., patients or caregivers with zero costs are not excluded in the average)

[1] Only two patients (one inpatient, one outpatient) had any DMCs, but costs are averaged over the total number of patients

[2] Productivity losses are averaged over the total number of patients in each category (not averaged over the total number of caregivers); 6 outpatients and 2 inpatients had caregivers who reported wage losses, but wages are averaged over the total number of outpatients (n=31) and inpatients (n=9)

[3] Costs are first summed across categories for each patient and averaged across total patients.

For peer review only - http://bmjopen.bmj.com/site/about/guidelines.xhtml

Table 3. Scenario analyses of mean indirect costs for cholera cases with reported wage losses and assuming minimum wage in Nsanje District, Malawi, 2016–2020

	**Scenario 1: Among those with self-reported wages only[1]<br>Patients: n=5<br>Caregivers: n=15**		**% of total indirect costs**	**Scenario 2: Self-reported + minimum wage for patients aged 15–50 years<br>Patients: n=11<br>Caregivers: n=45**		**% of total indirect costs**	**Scenario 3: Self-reported + minimum wage for patients aged >5 years<br>Patients: n=19<br>Caregivers: n=45**		**% of total indirect costs**
	**2020 MWK[3] (min, max)**	**2020 USD (min, max)**		**MWK (min, max)**	**2020 USD (min, max)**		**MWK (min, max)**	**2020 USD (min, max)**	
**Indirect, productivity losses**									
Patients[1]	7,807 (5,008–12,519)	10.60 (6.80–17.00)	63%	6,506 (2,692–12,519)	8.83 (3.66–17.00)	69%	7,161 (2,019–16,154)	9.78 (2.74–21.93)	71%
Caregivers	8,558 (823–25,039)	6.36 (1.12–33.99)	38%	5,306 (823–25,039)	3.94 (1.12–33.99)	31%	5,306 (823–25,039)	3.94 (1.12–33.99)	29%
**Total indirect costs**	**16,365**	**16.96**		**11,812**	**12.77**		**12,467**	**13.72**	

[1] All 5 patients who worked for a wage were outpatient

[2] MWK= Malawian kwacha

For peer review only - http://bmjopen.bmj.com/site/about/guidelines.xhtml

Table 4. Financial and food insecurity of cholera cases in Nsanje District, Malawi, 2016–2020

	Outpatient (n=31) n (%)		Inpatient (n=9) n (%)		Total (n=40) n (%)	
**Borrowing money to pay for health care services**						
Did not borrow money	17	(55)	3	(33)	20	(50)
Borrowed money from a family member	6	(19)	0	(0)	6	(15)
Borrowed money from a friend	4	(13)	5	(56)	9	(23)
Borrowed money from a cooperative	0	(0)	1	(11)	1	(3)
Borrowed money from a neighbor	1	(3)	0	(0)	1	(3)
Borrowed money from the village chief	1	(3)	0	(0)	1	(3)
Borrowed money from boss/employer	1	(3)	0	(0)	1	(3)
Borrowed money from church	1	(3)	0	(0)	1	(3)
**Sold items to pay for health care services**						
Did not sell anything	29	(94)	4	(44)	33	(83)
Rented out rice and maize plots	0	(0)	4	(44)	4	(10)
Sold radio	1	(3)	0	(0)	1	(3)
Sold livestock	1	(3)	1	(11)	2	(5)
**Frequency of household going without food in the past year**						
Never	4	(13)	1	(11)	5	(13)
Once every 3 months	7	(23)	1	(11)	8	(20)
Once per month	3	(10)	2	(22)	5	(13)
Twice per month	2	(6)	1	(11)	3	(8)
Once per week	8	(26)	1	(11)	9	(23)
Almost every day	3	(10)	0	(0)	3	(8)
Unknown/Missing	4	(13)	3	(33)	7	(18)

For peer review only - http://bmjopen.bmj.com/site/about/guidelines.xhtml

Cholera Surveillance In Malawi (CSIMA) Program
NSANJE DISTRICT USE ONLY

Form S.I.#:__________________ **Study label**

(To be filled by Data Management) N☐☐-☐☐☐☐

D-1: Cost-of-Illness Survey
First Interview (Day 0,1,2,3, 4, 5, 6, or 7)
Case Group: ☐ Clinical Case ☐ RDT Case ☐ Laboratory Case

Place of interview: ☐Health facility / ☐Home / ☐Other, specify: ___________________________
If at health facility, indicate point of care: ☐Out-Patient / ☐In-Patient
If In-Patient, name of admission ward: ___________________________
Date of Admission (dd/mm/yyyy): _____/_____/__________
Date of Discharge (dd/mm/yyyy): _____/_____/__________ [Ç: Look this up in the study facility record]

For SURVEY FIELD WORKER and PATIENT IDENTITY VERIFICATION
Please fill out this section with relevant information from the Facility Recruitment Form (# refers to question number on Recruitment Form).

Patient's Full Name (#2 & #3): ____
Patient's Clan (*mfunda*) Name (#5): ____
Contact Phone Number (#8): ____
Health facility where patient was recruited (See Study ID 2-digit):______________________________
Patient's Sex (#10): ☐ Male ☐ Female
Patient's Age (#11, #11.1): ☐☐ Years **OR** ☐☐ Months (if child is ≤24 months)
Scheduled Date of Interview (DD/MM/YYYY) (#35): ☐☐/☐☐/☐☐☐☐

For INTERVIEWER

Your Name: ____
Have you (interviewer) called to confirm the interview 1 day in advance? ☐ Yes ☐ No
Did you take an empty bucket with you? ☐ Yes ☐ No ☐ None available

Instructions for Interviewer:
*Please check that you have completed the **SES-WASH Survey** before starting this **D-1 Cost-of-Illness Survey, First Interview**.*

Use this instrument to interview an adult (head of household, parent, or guardian) that is familiar with the patient and his/her treatment over the last few days. If the patient is ≥18 years old, attempt to interview the patient unless s/he requires a caretaker (e.g., patient is elderly and/or has mental illness).

*Any places that say "**SCRIPT**" should be read out loud by you, the interviewer, to the patient or person answering on behalf of the patient. Any places that say "**Interviewer**" are notes for you to be aware of before continuing to the next survey question.*

For peer review only - http://bmjopen.bmj.com/site/about/guidelines.xhtml

Cholera Surveillance In Malawi (CSIMA) Program
NSANJE DISTRICT USE ONLY

Form S.I.#:__________________ **Study label**

(To be filled by Data Management) N☐☐-☐☐☐☐

Verifying Patient Identity

SCRIPT: My records show that you (or the patient) went or were taken to the [**health facility**] about 0 to 7 days ago. Can you please tell me your name in the village or your father's name to verify your identify?

Interviewer: *Please also verify visually the patient's sex as indicated above. The age should be within 1 year.*

1	Is this correct?	☐ Yes → **continue to 2**	☐ No	☐ Don't know

Interviewer: *The patient (or parent/guardian) should have responded "**Yes**" to question 1. If s/he did not, ask if the patient is another household member or if there has been a mistake. If the problem cannot be resolved and the individual who was treated at the health facility cannot be identified, **STOP the interview**.*

2	Could you please tell me your relationship to the patient?	☐ 1 = Self (patient)	☐ 2 = Mother	☐ 3 = Father
		☐ 4 = Sister	☐ 5 = Brother	☐ 6 = Grandmother
		☐ 7 = Grandfather		
		☐ 8 = Other (specify): ______________		

SCRIPT: As you may recall, when you visited the [**health facility**] 0–7 days ago, you were read an informed consent form that described participation in this survey and study. We would like to remind you that there are no direct risks from participation in this study. Participation is free of charge, and will not affect your treatment in any way now nor in the future at any health facility.

The purpose of this current survey is to understand how your (or the patient's) cholera episode has affected him/her financially. The results of this study will help local health staff in improving our knowledge of how cholera affects people in Malawi so that we can be prepared for future cholera outbreaks.

As a thank you for participating in this Cost-of-Illness Survey, we will give you a bucket that you may use in various ways, such as drawing water or for washing your hands at the end of the survey. If you have a **health passport** available, it may be helpful to use it to help you answer these questions.

SECTION A: Patient History Before Day 0

SCRIPT: After the doctor examined you clinically / after taking a stool sample recently during your (or the patient's) visit to [**health facility**], the doctor suspected / we found the cholera bacteria present. I am going to ask you about how this recent cholera episode affected you (or the patient) and anyone that may have gone with you (or the patient) while seeking treatment.

First, I would like to understand how much you (or the patient) had to spend on any healthcare services (including traditional healers) **before** visiting the health facility on [**Day 0**].

3	How many ***days*** were you (or the patient) sick with diarrhea before going to [**health facility**] 3 to 7 days ago where you were diagnosed?	☐☐ Days<br>☐ 99 = Don't know

For peer review only - http://bmjopen.bmj.com/site/about/guidelines.xhtml

Cholera Surveillance In Malawi (CSIMA) Program
NSANJE DISTRICT USE ONLY

Form S.I.#:_________________ **Study label**

(To be filled by Data Management) N☐☐-☐☐☐☐

4	Before going to [**health facility**] for care 3 to 7 days ago, did you (or the patient) receive treatment for illness somewhere else?	☐ 1 = Yes<br>☐ 2 = No → **skip to 34 (SECTION C)**<br>☐ 9 = Don't know → **skip to 34 (SECTION C)**
5	How many treatment locations did you visit?<br>*Examples of treatment locations: traditional healer, CHAM facility, public facility, and private hospital. Each of these should be counted as separate treatment locations.*	☐☐ Treatment location(s)

SECTION B: Health Facility Visits Before Day 0

Instructions: *Please fill out the entire column (top to bottom) for each time the patient sought care at a treatment location before Day 0. Please provide the following details in* ***local Malawian kwacha (MWK) currency****. If additional days are needed, please use the additional pages provided.*

Questions #6 through #33 *must be filled out for each visit. The first column should be the patient's earliest visit. The last column filled out should be the visit closest to the health facility visit in which the patient was enrolled. Do not include the Day 0 health facility visit.*

Example: *If there was a visit to a traditional healer and a health facility on a single day, then this is 2 visits (2 columns) that must be recorded.*

Item		Visits Before Day 0			
		**Visit 1 (Earliest Visit)**	**Visit 2**	**Visit 3**	**Visit 4**
6	Where did you seek treatment?<br>**Code:**<br>1 = District hospital<br>2 = Health centre/post<br>3 = CHAM/private<br>4 = Traditional healer/herbalist<br>5 = Other (specify)<br>9 = Don't know	☐<br>If "**5**" entered, specify: ________	☐<br>If "**5**" entered, specify: ________	☐<br>If "**5**" entered, specify: ________	☐<br>If "**5**" entered, specify: ________
7	During this visit, did you (or the patient) have to buy food and/or water for yourself?	☐ 1 = Yes<br>☐ 2 = No → **skip to 9**<br>☐ 9 = Don't know → **skip to 9**	☐ 1 = Yes<br>☐ 2 = No → **skip to 9**<br>☐ 9 = Don't know → **skip to 9**	☐ 1 = Yes<br>☐ 2 = No → **skip to 9**<br>☐ 9 = Don't know → **skip to 9**	☐ 1 = Yes<br>☐ 2 = No → **skip to 9**<br>☐ 9 = Don't know → **skip to 9**
8	How much did you pay for the food and/or water?<br>*Write "0" if nothing was paid.*	**MWK**<br>☐ 9 = Don't know	**MWK**<br>☐ 9 = Don't know	**MWK**<br>☐ 9 = Don't know	**MWK**<br>☐ 9 = Don't know

For peer review only - http://bmjopen.bmj.com/site/about/guidelines.xhtml

Cholera Surveillance In Malawi (CSIMA) Program
NSANJE DISTRICT USE ONLY

Form S.I.#:________________ **Study label**

(To be filled by Data Management) N☐☐-☐☐☐☐

9	Not including food and/or water, how much did you pay for this visit? *Write "0" if nothing was paid.*	**MWK** ☐ 9 = Don't know	**MWK** ☐ 9 = Don't know	**MWK** ☐ 9 = Don't know	**MWK** ☐ 9 = Don't know
10	What was this payment for? *(Specify up to 3 choices)* **Code:** 1 = Medicine/drugs 2 = Diagnostic/lab fee 3 = Doctor, nurse, or herbalist fee 4 = Other (specify) 6 = Does not apply 9 = Don't know	Response 1: ☐ Response 2: ☐ Response 3: ☐ If "**4**" entered, specify: ________	Response 1: ☐ Response 2: ☐ Response 3: ☐ If "**4**" entered, specify: ________	Response 1: ☐ Response 2: ☐ Response 3: ☐ If "**4**" entered, specify: ________	Response 1: ☐ Response 2: ☐ Response 3: ☐ If "**4**" entered, specify: ________
11	Did you pay for anything outside of this treatment location? *Example: the healer asked you to buy **roots** for medicinal use after your visit.*	☐ 1 = Yes (specify): ________ ☐ 2 = No → **skip to 13** ☐ 9 = Don't know → **skip to 13**	☐ 1 = Yes (specify): ________ ☐ 2 = No → **skip to 13** ☐ 9 = Don't know → **skip to 13**	☐ 1 = Yes (specify): ________ ☐ 2 = No → **skip to 13** ☐ 9 = Don't know → **skip to 13**	☐ 1 = Yes (specify): ________ ☐ 2 = No → **skip to 13** ☐ 9 = Don't know → **skip to 13**
12	How much did it cost?	**MWK** ☐ 9 = Don't know	**MWK** ☐ 9 = Don't know	**MWK** ☐ 9 = Don't know	**MWK** ☐ 9 = Don't know
13	Who paid for the visit? *(Specify up to 3 choices)*	Response 1: ☐ Response 2: ☐	Response 1: ☐ Response 2: ☐	Response 1: ☐ Response 2: ☐	Response 1: ☐ Response 2: ☐

IVI_CSIMA_ v4.1, 24-April-2017

For peer review only

Cholera Surveillance In Malawi (CSIMA) Program
NSANJE DISTRICT USE ONLY

Form S.I.#:__________________ **Study label**

(To be filled by Data Management) N☐☐-☐☐☐☐

	**Code:**<br>1 = Self<br>2 = Patient's family<br>3 = Other (specify), including insurance<br>6 = Did not pay anything<br>9 = Don't know	Response 3: ☐<br>If "**3**" entered, specify:<br>__________	Response 3: ☐<br>If "**3**" entered, specify:<br>__________	Response 3: ☐<br>If "**3**" entered, specify:<br>__________	Response 3: ☐<br>If "**3**" entered, specify:<br>__________
**SCRIPT**: Now, I would like to understand how you **got to the treatment location and then back home**. I will also ask you questions about **whether anyone came with you** to the treatment location.					
14	How did you get to the treatment facility?<br>*(Specify up to 3 choices)*<br>**Code:**<br>1 = On foot/walking<br>2 = Bicycle<br>3 = Motorcycle<br>4 = Minibus<br>5 = Private car<br>6 = Horse cart<br>7 = Boat<br>8 = Other (specify)<br>9 = Don't know	**Travelled by:**<br>Response 1: ☐<br>Response 2: ☐<br>Response 3: ☐<br>If "**8**" entered, specify:<br>__________	**Travelled by:**<br>Response 1: ☐<br>Response 2: ☐<br>Response 3: ☐<br>If "**8**" entered, specify:<br>__________	**Travelled by:**<br>Response 1: ☐<br>Response 2: ☐<br>Response 3: ☐<br>If "**8**" entered, specify:<br>__________	**Travelled by:**<br>Response 1: ☐<br>Response 2: ☐<br>Response 3: ☐<br>If "**8**" entered, specify:<br>__________
15	How long did it take for the patient to get there?	**Travel time:**<br>☐☐☐ **Minutes**	**Travel time:**<br>☐☐☐ **Minutes**	**Travel time:**<br>☐☐☐ **Minutes**	**Travel time:**<br>☐☐☐ **Minutes**
16	How much did it cost for the patient to get there?<br>*Write "0" if nothing was paid.*	**MWK**<br>☐ 999 = Don't know	**MWK**<br>☐ 999 = Don't know	**MWK**<br>☐ 999 = Don't know	**MWK**<br>☐ 999 = Don't know
17	Did you travel to the location alone?	☐ 1 = Yes<br>→ **skip to 20**<br>☐ 2 = No	☐ 1 = Yes<br>→ **skip to 20**<br>☐ 2 = No	☐ 1 = Yes<br>→ **skip to 20**<br>☐ 2 = No	☐ 1 = Yes<br>→ **skip to 20**<br>☐ 2 = No
18	How many people came with you (excluding the patient)?	☐☐ People<br>☐ 99 = Don't know	☐☐ People<br>☐ 99 = Don't know	☐☐ People<br>☐ 99 = Don't know	☐☐ People<br>☐ 99 = Don't know

For peer review only - http://bmjopen.bmj.com/site/about/guidelines.xhtml

Cholera Surveillance In Malawi (CSIMA) Program
NSANJE DISTRICT USE ONLY

Form S.I.#:__________________ **Study label**

(To be filled by Data Management) N☐☐-☐☐☐☐

	If patient answered "No" to 17, this ***cannot*** *be "00"*				
19	How much did it cost for <u>each person</u> that came with you?<br><br>*Write "0" if nothing was paid.*	**MWK Per person**<br>☐ 999 = Don't know	**MWK Per person**<br>☐ 999 = Don't know	**MWK Per person**<br>☐ 999 = Don't know	**MWK Per person**<br>☐ 999 = Don't know
20	Did anybody follow you to the treatment location (did they come separately)?	☐ 1 = Yes<br>☐ 2 = No<br>**→ skip to 25**	☐ 1 = Yes<br>☐ 2 = No<br>**→ skip to 25**	☐ 1 = Yes<br>☐ 2 = No<br>**→ skip to 25**	☐ 1 = Yes<br>☐ 2 = No<br>**→ skip to 25**
21	How many people met you at the location?<br><br>*If patient answered "Yes" to 20, this* ***cannot*** *be "00"*	☐☐ People<br>☐ 99 = Don't know	☐☐ People<br>☐ 99 = Don't know	☐☐ People<br>☐ 99 = Don't know	☐☐ People<br>☐ 99 = Don't know
22	How did the person(s) get to the location?<br>*(Specify up to 3 choices)*<br><br>**Code:**<br>1 = On foot/walking<br>2 = Bicycle<br>3 = Motorcycle<br>4 = Minibus<br>5 = Private car<br>6 = Horse cart<br>7 = Boat<br>8 = Other (specify)<br>9 = Don't know	**Travelled by:**<br>Response 1: ☐<br>Response 2: ☐<br>Response 3: ☐<br>If "**8**" entered, specify:<br>____________	**Travelled by:**<br>Response 1: ☐<br>Response 2: ☐<br>Response 3: ☐<br>If "**8**" entered, specify:<br>____________	**Travelled by:**<br>Response 1: ☐<br>Response 2: ☐<br>Response 3: ☐<br>If "**8**" entered, specify:<br>____________	**Travelled by:**<br>Response 1: ☐<br>Response 2: ☐<br>Response 3: ☐<br>If "**8**" entered, specify:<br>____________
23	How long did it take the person(s)?<br><br>*Enter time <u>per person</u>.*	**Travel time:**<br>☐☐☐<br>**Minutes per person**<br>☐ 999 = Don't know	**Travel time:**<br>☐☐☐<br>**Minutes per person**<br>☐ 999 = Don't know	**Travel time:**<br>☐☐☐<br>**Minutes per person**<br>☐ 999 = Don't know	**Travel time:**<br>☐☐☐<br>**Minutes per person**<br>☐ 999 = Don't know

For peer review only - http://bmjopen.bmj.com/site/about/guidelines.xhtml

For peer review only

Cholera Surveillance In Malawi (CSIMA) Program
NSANJE DISTRICT USE ONLY

Form S.I.#:__________________ **Study label**

(To be filled by Data Management) N☐☐-☐☐☐☐

24	How much did it cost the person(s)?<br><br>*Enter cost per person. Write "0" if nothing was paid.*	**MWK Per person**<br>☐ 999 = Don't know	**MWK Per person**<br>☐ 999 = Don't know	**MWK Per person**<br>☐ 999 = Don't know	**MWK Per person**<br>☐ 999 = Don't know
25	While at the facility, how much did everyone who came with you or met you at the treatment facility spend on food and/or water (excluding patient)?<br><br>*Enter cost for all persons combined. Write "0" if no food and/or water were purchased.*	**MWK Total for all persons**<br>☐ Respondent unsure, but estimated<br>☐ 666 = Went alone<br>☐ 999 = Don't know	**MWK Total for all persons**<br>☐ Respondent unsure, but estimated<br>☐ 666 = Went alone<br>☐ 999 = Don't know	**MWK Total for all persons**<br>☐ Respondent unsure, but estimated<br>☐ 666 = Went alone<br>☐ 999 = Don't know	**MWK Total for all persons**<br>☐ Respondent unsure, but estimated<br>☐ 666 = Went alone<br>☐ 999 = Don't know
26	How much did the person(s) spend on lodging (total) if an overnight stay was needed?<br><br>*Enter cost for all persons combined. Write "0" if no overnight stay was needed.*	**MWK Total for all persons**<br>☐ 666 = Was alone<br>☐ 999 = Don't know	**MWK Total for all persons**<br>☐ 666 = Was alone<br>☐ 999 = Don't know	**MWK Total for all persons**<br>☐ 666 = Was alone<br>☐ 999 = Don't know	**MWK Total for all persons**<br>☐ 666 = Was alone<br>☐ 999 = Don't know
**SCRIPT:** Now, I would like to understand how you (or the patient) **travelled back home after the visit** to the treatment location.					
27	How did you get home?<br>*(Specify up to 3 choices)*	**Travelled by:**<br>Response 1: ☐	**Travelled by:**<br>Response 1: ☐	**Travelled by:**<br>Response 1: ☐	**Travelled by:**<br>Response 1: ☐

For peer review only - http://bmjopen.bmj.com/site/about/guidelines.xhtml

Cholera Surveillance In Malawi (CSIMA) Program
NSANJE DISTRICT USE ONLY

Form S.I.#:__________________ **Study label**

(To be filled by Data Management) N☐☐-☐☐☐☐

	**Code:**<br>1 = On foot/walking<br>2 = Bicycle<br>3 = Motorcycle<br>4 = Minibus<br>5 = Private car<br>6 = Horse cart<br>7 = Boat<br>8 = Other (specify)<br>9 = Don't know	Response 2: ☐<br>Response 3: ☐<br>If "**8**" entered, specify:<br>______________	Response 2: ☐<br>Response 3: ☐<br>If "**8**" entered, specify:<br>______________	Response 2: ☐<br>Response 3: ☐<br>If "**8**" entered, specify:<br>______________	Response 2: ☐<br>Response 3: ☐<br>If "**8**" entered, specify:<br>______________
28	How long did it take for the patient?	**Travel time:**<br>☐☐☐ **Minutes**<br>☐ 999 = Don't know	**Travel time:**<br>☐☐☐ **Minutes**<br>☐ 999 = Don't know	**Travel time:**<br>☐☐☐ **Minutes**<br>☐ 999 = Don't know	**Travel time:**<br>☐☐☐ **Minutes**<br>☐ 999 = Don't know
29	How much did it cost for the patient?<br>*Write "0" if nothing was paid.*	**MWK**<br>☐ 999 = Don't know	**MWK**<br>☐ 999 = Don't know	**MWK**<br>☐ 999 = Don't know	**MWK**<br>☐ 999 = Don't know
30	Did you travel back to your home <u>alone</u>?	☐ 1 = Yes<br>→ **skip to 33**<br>☐ 2 = No	☐ 1 = Yes<br>→ **skip to 33**<br>☐ 2 = No	☐ 1 = Yes<br>→ **skip to 33**<br>☐ 2 = No	☐ 1 = Yes<br>→ **skip to 33**<br>☐ 2 = No
31	How many people travelled home with you (<u>excluding</u> you)?<br>*If patient answered "No" to 30, this **cannot** be "00"*	☐☐ People<br>☐ 99 = Don't know	☐☐ People<br>☐ 99 = Don't know	☐☐ People<br>☐ 99 = Don't know	☐☐ People<br>☐ 99 = Don't know
32	How much did it cost for <u>each person</u> that came with you?<br>*Write "0" if nothing was paid.*	**MWK**<br>**Per person**<br>☐ 999 = Don't know	**MWK**<br>**Per person**<br>☐ 999 = Don't know	**MWK**<br>**Per person**<br>☐ 999 = Don't know	**MWK**<br>**Per person**<br>☐ 999 = Don't know
Interviewer: *Please add any additional notes regarding travelling to and from the treatment center in **Question #33**.*<br><br>***For example:*** *"While patient was at CHAM facility with his/her mother, the patient required food. The mother traveled home by foot to bring the home-cooked food to the CHAM facility for the patient to eat."*					
33	Additional notes regarding patient or companion travel.				

CHECK for Interviewer

IVI_CSIMA_ v4.1, 24-April-2017

For peer review only - http://bmjopen.bmj.com/site/about/guidelines.xhtml

Cholera Surveillance In Malawi (CSIMA) Program
NSANJE DISTRICT USE ONLY

Form S.I.#:__________________ **Study label**

(To be filled by Data Management) N☐☐-☐☐☐☐

How many treatment locations did the patient specify in Question #5?

☐☐ Treatment location(s)

Please check that the **number of treatment location(s) matches the number of columns** that you have filled out in **Section B** before proceeding to **Section C**. **Questions #6 through #33** must be filled out for each visit. If there was a visit to a traditional healer and a health facility on a single day, then this is 2 visits (2 columns) that must be recorded.

Have you verified?

☐ Yes → **continue to Section C**

SECTION C: Health Facility Visits Starting at Day 0

SCRIPT: Now, I would like to understand how much you (or the patient) had to spend on any healthcare services (including traditional healers) **starting on the day you first visited the health facility** where we diagnosed and treated you on Day 0.

Instructions: *Please fill out the entire column (top to bottom) for each time the patient sought care at a treatment location starting at **Day 0**. Please provide the following details in **local Malawian kwacha (MWK) currency**. If additional days are needed, please use the additional pages provided.*

Questions #34 through #61 *must be filled out for each visit. The first column should be the patient's first visit to the health facility in which the patient was enrolled (i.e., Day 0). The last column filled out should be the visit closest to today's interview. Do **not** include any visits to any type of treatment location today.*

Item		Visits Starting at Day 0			
		**Day 0**	**Visit 1**	**Visit 2**	**Visit 3**
34	Where did you seek treatment?<br><br>**Code:**<br>1 = District hospital<br>2 = Health centre/post<br>3 = CHAM/private<br>4 = Traditional healer/herbalist<br>5 = Other (specify)<br>9 = Don't know	☐<br>If "**5**" entered, specify:<br>______	☐<br>If "**5**" entered, specify:<br>______	☐<br>If "**5**" entered, specify:<br>______	☐<br>If "**5**" entered, specify:<br>______
35	During this visit, did you (or the patient) have to buy food and/or water for yourself?	☐ 1 = Yes<br>☐ 2 = No<br>→ **skip to 37**<br>☐ 9 = Don't know<br>→ **skip to 37**	☐ 1 = Yes<br>☐ 2 = No<br>→ **skip to 37**<br>☐ 9 = Don't know<br>→ **skip to 37**	☐ 1 = Yes<br>☐ 2 = No<br>→ **skip to 37**<br>☐ 9 = Don't know<br>→ **skip to 37**	☐ 1 = Yes<br>☐ 2 = No<br>→ **skip to 37**<br>☐ 9 = Don't know<br>→ **skip to 37**
36	How much did you pay for the food and/or water?	**MWK**	**MWK**	**MWK**	**MWK**

For peer review only - http://bmjopen.bmj.com/site/about/guidelines.xhtml

Cholera Surveillance In Malawi (CSIMA) Program
NSANJE DISTRICT USE ONLY

Form S.I.#:__________________ **Study label**

(To be filled by Data Management) N☐☐-☐☐☐☐

		☐ 9 = Don't know	☐ 9 = Don't know	☐ 9 = Don't know	☐ 9 = Don't know
37	Not including food and/or water, how much did you pay for this visit?<br><br>*Write "0" if nothing was paid.*	**MWK**<br>☐ 9 = Don't know	**MWK**<br>☐ 9 = Don't know	**MWK**<br>☐ 9 = Don't know	**MWK**<br>☐ 9 = Don't know
38	What was this payment for? *(Specify up to 3 choices)*<br><br>**Code:**<br>1 = Medicine/drugs<br>2 = Diagnostic/lab fee<br>3 = Doctor, nurse, or herbalist fee<br>4 = Other (specify)<br>6 = Does not apply<br>9 = Don't know	Response 1: ☐<br>Response 2: ☐<br>Response 3: ☐<br>If "**4**" entered, specify:<br>______________	Response 1: ☐<br>Response 2: ☐<br>Response 3: ☐<br>If "**4**" entered, specify:<br>______________	Response 1: ☐<br>Response 2: ☐<br>Response 3: ☐<br>If "**4**" entered, specify:<br>______________	Response 1: ☐<br>Response 2: ☐<br>Response 3: ☐<br>If "**4**" entered, specify:<br>______________
39	Did you pay for anything outside of this treatment location?<br><br>*Example: the healer asked you to buy **roots** for medicinal use after your visit.*	☐ 1 = Yes (specify):<br>______________<br>☐ 2 = No<br>**→ skip to 41**<br>☐ 9 = Don't know<br>**→ skip to 41**	☐ 1 = Yes (specify):<br>______________<br>☐ 2 = No<br>**→ skip to 41**<br>☐ 9 = Don't know<br>**→ skip to 41**	☐ 1 = Yes (specify):<br>______________<br>☐ 2 = No<br>**→ skip to 41**<br>☐ 9 = Don't know<br>**→ skip to 41**	☐ 1 = Yes (specify):<br>______________<br>☐ 2 = No<br>**→ skip to 41**<br>☐ 9 = Don't know<br>**→ skip to 41**
40	How much did it cost?	**MWK**<br>☐ 9 = Don't know	**MWK**<br>☐ 9 = Don't know	**MWK**<br>☐ 9 = Don't know	**MWK**<br>☐ 9 = Don't know
41	Who paid for the visit?	Response 1: ☐	Response 1: ☐	Response 1: ☐	Response 1: ☐

For peer review only - http://bmjopen.bmj.com/site/about/guidelines.xhtml
For peer review only

Cholera Surveillance In Malawi (CSIMA) Program
NSANJE DISTRICT USE ONLY

Form S.I.#:__________________ **Study label**

(To be filled by Data Management) **N**☐☐-☐☐☐☐

	(Specify up to 3 choices)<br><br>**Code:**<br>1 = Self<br>2 = Patient's family<br>3 = Other (specify), including insurance<br>6 = Did not pay anything<br>9 = Don't know	Response 2: ☐<br>Response 3: ☐<br><br>If "**3**" entered, specify:<br>__________	Response 2: ☐<br>Response 3: ☐<br><br>If "**3**" entered, specify:<br>__________	Response 2: ☐<br>Response 3: ☐<br><br>If "**3**" entered, specify:<br>__________	Response 2: ☐<br>Response 3: ☐<br><br>If "**3**" entered, specify:<br>__________
**SCRIPT:** Now, I would like to understand how you **got to the treatment location and then back home**. I will also ask you questions about **whether anyone came with you** to the treatment location.					
42	How did you get to the treatment facility?<br>*(Specify up to 3 choices)*<br><br>**Code:**<br>1 = On foot/walking<br>2 = Bicycle<br>3 = Motorcycle<br>4 = Minibus<br>5 = Private car<br>6 = Horse cart<br>7 = Boat<br>8 = Other (specify)<br>9 = Don't know	**Travelled by:**<br>Response 1: ☐<br>Response 2: ☐<br>Response 3: ☐<br><br>If "**8**" entered, specify:<br>__________	**Travelled by:**<br>Response 1: ☐<br>Response 2: ☐<br>Response 3: ☐<br><br>If "**8**" entered, specify:<br>__________	**Travelled by:**<br>Response 1: ☐<br>Response 2: ☐<br>Response 3: ☐<br><br>If "**8**" entered, specify:<br>__________	**Travelled by:**<br>Response 1: ☐<br>Response 2: ☐<br>Response 3: ☐<br><br>If "**8**" entered, specify:<br>__________
43	How long did it take for the patient to get there?	**Travel time:**<br>☐☐☐ **Minutes**	**Travel time:**<br>☐☐☐ **Minutes**	**Travel time:**<br>☐☐☐ **Minutes**	**Travel time:**<br>☐☐☐ **Minutes**
44	How much did it cost for the patient to get there?<br><br>*Write "0" if nothing was paid.*	**MWK**<br><br>☐ 999 = Don't know	**MWK**<br><br>☐ 999 = Don't know	**MWK**<br><br>☐ 999 = Don't know	**MWK**<br><br>☐ 999 = Don't know
45	Did you travel to the location <u>alone</u>?	☐ 1 = Yes<br>→ **skip to 48**<br><br>☐ 2 = No	☐ 1 = Yes<br>→ **skip to 48**<br><br>☐ 2 = No	☐ 1 = Yes<br>→ **skip to 48**<br><br>☐ 2 = No	☐ 1 = Yes<br>→ **skip to 48**<br><br>☐ 2 = No
46	How many people came with you	☐☐ People	☐☐ People	☐☐ People	☐☐ People

IVI_CSIMA_ v4.1, 24-April-2017

For peer review only - http://bmjopen.bmj.com/site/about/guidelines.xhtml

For peer review only

Cholera Surveillance In Malawi (CSIMA) Program
NSANJE DISTRICT USE ONLY

Form S.I.#:________________

(To be filled by Data Management)

Study label

N☐☐-☐☐☐☐

	(excluding the patient)? *If patient answered "No" to 45, this **cannot** be "00"*	☐ 99 = Don't know	☐ 99 = Don't know	☐ 99 = Don't know	☐ 99 = Don't know
47	How much did it cost for each person that came with you? *Write "0" if nothing was paid.*	**MWK Per person** ☐ 999 = Don't know	**MWK Per person** ☐ 999 = Don't know	**MWK Per person** ☐ 999 = Don't know	**MWK Per person** ☐ 999 = Don't know
48	Did anybody follow you to the treatment location (did they come separately)?	☐ 1 = Yes ☐ 2 = No **→ skip to 53**	☐ 1 = Yes ☐ 2 = No **→ skip to 53**	☐ 1 = Yes ☐ 2 = No **→ skip to 53**	☐ 1 = Yes ☐ 2 = No **→ skip to 53**
49	How many people met you at the location? *If patient answered "Yes" to 48, this **cannot** be "00"*	☐☐ People ☐ 99 = Don't know	☐☐ People ☐ 99 = Don't know	☐☐ People ☐ 99 = Don't know	☐☐ People ☐ 99 = Don't know
50	How did the person(s) get to the location? *(Specify up to 3 choices)* **Code:** 1 = On foot/walking 2 = Bicycle 3 = Motorcycle 4 = Minibus 5 = Private car 6 = Horse cart 7 = Boat 8 = Other (specify) 9 = Don't know	**Travelled by:** Response 1: ☐ Response 2: ☐ Response 3: ☐ If "**8**" entered, specify: ________	**Travelled by:** Response 1: ☐ Response 2: ☐ Response 3: ☐ If "**8**" entered, specify: ________	**Travelled by:** Response 1: ☐ Response 2: ☐ Response 3: ☐ If "**8**" entered, specify: ________	**Travelled by:** Response 1: ☐ Response 2: ☐ Response 3: ☐ If "**8**" entered, specify: ________
51	How long did it take the person(s)?	**Travel time:** ☐☐☐ **Minutes per person**	**Travel time:** ☐☐☐ **Minutes per person**	**Travel time:** ☐☐☐ **Minutes per person**	**Travel time:** ☐☐☐ **Minutes per person**

For peer review only - http://bmjopen.bmj.com/site/about/guidelines.xhtml
For peer review only

Cholera Surveillance In Malawi (CSIMA) Program

NSANJE DISTRICT USE ONLY

Form S.I.#:__________________

(To be filled by Data Management)

Study label

N☐☐-☐☐☐☐

	Enter time per person.	☐ 999 = Don't know	☐ 999 = Don't know	☐ 999 = Don't know	☐ 999 = Don't know
52	How much did it cost the person(s)? *Enter cost per person. Write "0" if nothing was paid.*	**MWK Per person** ☐ 999 = Don't know	**MWK Per person** ☐ 999 = Don't know	**MWK Per person** ☐ 999 = Don't know	**MWK Per person** ☐ 999 = Don't know
53	While at the facility, how much did everyone who came with you or met you at the treatment facility spend on food and/or water (excluding patient)? *Enter cost for all persons combined. Write "0" if no food and/or water were purchased.*	**MWK Total for all persons** ☐ Respondent unsure, but estimated ☐ 666 = Went alone ☐ 999 = Don't know	**MWK Total for all persons** ☐ Respondent unsure, but estimated ☐ 666 = Went alone ☐ 999 = Don't know	**MWK Total for all persons** ☐ Respondent unsure, but estimated ☐ 666 = Went alone ☐ 999 = Don't know	**MWK Total for all persons** ☐ Respondent unsure, but estimated ☐ 666 = Went alone ☐ 999 = Don't know
54	How much did the person(s) spend on lodging (total) if an overnight stay was needed? *Enter cost for all persons combined. Write "0" if no overnight stay was needed.*	**MWK Total for all persons** ☐ 666 = Was alone ☐ 999 = Don't know	**MWK Total for all persons** ☐ 666 = Was alone ☐ 999 = Don't know	**MWK Total for all persons** ☐ 666 = Was alone ☐ 999 = Don't know	**MWK Total for all persons** ☐ 666 = Was alone ☐ 999 = Don't know
**SCRIPT:** Now, I would like to understand how you (or the patient) **travelled back home after the visit** to the treatment location.					
55	How did you get home?	**Travelled by:**	**Travelled by:**	**Travelled by:**	**Travelled by:**

For peer review only - http://bmjopen.bmj.com/site/about/guidelines.xhtml

For peer review only

Cholera Surveillance In Malawi (CSIMA) Program
NSANJE DISTRICT USE ONLY

Form S.I.#:__________________ **Study label**

(To be filled by Data Management) **N**☐☐-☐☐☐☐

	(Specify up to 3 choices)<br><br>**Code:**<br>1 = On foot/walking<br>2 = Bicycle<br>3 = Motorcycle<br>4 = Minibus<br>5 = Private car<br>6 = Horse cart<br>7 = Boat<br>8 = Other (specify)<br>9 = Don't know	Response 1: ☐<br>Response 2: ☐<br>Response 3: ☐<br>If "**8**" entered, specify:<br>__________	Response 1: ☐<br>Response 2: ☐<br>Response 3: ☐<br>If "**8**" entered, specify:<br>__________	Response 1: ☐<br>Response 2: ☐<br>Response 3: ☐<br>If "**8**" entered, specify:<br>__________	Response 1: ☐<br>Response 2: ☐<br>Response 3: ☐<br>If "**8**" entered, specify:<br>__________
56	How long did it take for the patient?	**Travel time:**<br>☐☐☐ **Minutes**<br>☐ 999 = Don't know	**Travel time:**<br>☐☐☐ **Minutes**<br>☐ 999 = Don't know	**Travel time:**<br>☐☐☐ **Minutes**<br>☐ 999 = Don't know	**Travel time:**<br>☐☐☐ **Minutes**<br>☐ 999 = Don't know
57	How much did it cost for the patient?<br>*Write "0" if nothing was paid.*	**MWK**<br>☐ 999 = Don't know	**MWK**<br>☐ 999 = Don't know	**MWK**<br>☐ 999 = Don't know	**MWK**<br>☐ 999 = Don't know
58	Did you travel back to your home alone?	☐ 1 = Yes<br>→ **skip to 61**<br>☐ 2 = No	☐ 1 = Yes<br>→ **skip to 61**<br>☐ 2 = No	☐ 1 = Yes<br>→ **skip to 61**<br>☐ 2 = No	☐ 1 = Yes<br>→ **skip to 61**<br>☐ 2 = No
59	How many people travelled home with you (excluding you)?<br>*If patient answered "No" to 58, this* ***cannot*** *be "00"*	☐☐ People<br>☐ 99 = Don't know	☐☐ People<br>☐ 99 = Don't know	☐☐ People<br>☐ 99 = Don't know	☐☐ People<br>☐ 99 = Don't know
60	How much did it cost for each person that came with you?<br>*Write "0" if nothing was paid.*	**MWK**<br>**Per person**<br>☐ 999 = Don't know	**MWK**<br>**Per person**<br>☐ 999 = Don't know	**MWK**<br>**Per person**<br>☐ 999 = Don't know	**MWK**<br>**Per person**<br>☐ 999 = Don't know
Interviewer: *Please add any additional notes regarding travelling to and from the treatment center in* ***Question #61****.*<br>***For example:*** *"While patient was at CHAM facility with his/her mother, the patient required food. The mother traveled home by foot (30 minutes roundtrip) to bring the home-cooked food to the CHAM facility for the patient to eat."*					
61	Additional notes regarding patient or				

IVI_CSIMA_ v4.1, 24-April-2017

For peer review only - http://bmjopen.bmj.com/site/about/guidelines.xhtml

Cholera Surveillance In Malawi (CSIMA) Program
NSANJE DISTRICT USE ONLY

Form S.I.#:__________________ **Study label**

(To be filled by Data Management) N☐☐-☐☐☐☐

	companion travel.				

CHECK for Interviewer

SCRIPT: Did you go anywhere else to seek treatment for your cholera since your recent health facility visit 3 to 7 days ago? Remember, treatment locations also include the traditional healer and places other than the health facility where we provided you (or the patient) treatment.

Interviewer: *If the patient says "Yes," please fill out another column starting from* ***Question #34 through #61****. Repeat this process until the patient says "No."*

Have you verified that the patient has not gone anywhere else for care since his/her Day 0 visit?

☐ Yes → **continue to Section D**

SECTION D: Indirect Costs: Productivity Losses & Opportunity Costs

SCRIPT: Now, I would like to ask you about the ways you (or the patient's) cholera illness affected those around you. Sometimes, people with cholera feel so sick that they cannot perform any of their usual activities. Instead, they have to rest and stay in bed. Other people around the patient may not be as sick, and can still do their usual activities. But, they may not be able to perform their activities as well as normal because they may have to help or take care of the patient.

When you answer these questions, please think about the entire time period when you were sick with cholera, starting with before you (or the patient) went to the health facility for diagnosis.

62	In **total**, how many days have you (or the patient) been sick with cholera since the symptoms started?	☐☐ Days	☐ 99 = Don't know
63	How many days were you (or the patient) **completely unable** to do any of your usual activities while sick?	☐☐ Days	☐ 99 = Don't know
64	How many days were you (or the patient) able to perform **some but not all** of your usual activities while sick?	☐☐ Days	☐ 99 = Don't know
65	How many days were you (or the patient) able to perform **all** of your usual activities while sick?	☐☐ Days	☐ 99 = Don't know

ERROR CHECK: *Please check that the number of days of no activity, some activity, and normal activity add up to the total number of days ill. If the days do not add up correctly, please review* ***Questions #62 through #65*** *with the patient.*

Days of **no activity** while ill (**Question #63**)	+	Days of **some activity** while ill (**Question #64**)	+	Days of **normal activity** while ill (**Question #65**)	=	Total number of days ill (**Question #62**)
☐☐ Days	+	☐☐ Days	+	☐☐ Days	=	☐☐ Days

66	What would you (or the patient) be doing if you had not been sick?<br><br>*If the patient is an infant/toddler, select "5" for Leisure or play time. Check all that apply.*	☐ 1 = Going to school<br>☐ 2 = Working for a wage (e.g. for an employer, paid)<br>☐ 3 = Working for self (e.g. fishing or farming, unpaid)<br>☐ 4 = Attending church<br>☐ 5 = Leisure or play time<br>☐ 6 = Helping a parent<br>☐ 7 = Housework (e.g. cooking, cleaning, child care)<br>☐ 8 = Other (specify): ______________________

For peer review only - http://bmjopen.bmj.com/site/about/guidelines.xhtml

Cholera Surveillance In Malawi (CSIMA) Program
NSANJE DISTRICT USE ONLY

Form S.I.#:__________________ **Study label**

(To be filled by Data Management) N☐☐-☐☐☐☐

67	Do you (or the patient) work for a wage (cash or in-kind)?	☐ 1 = Yes, paid in cash (specify amount **per day**): ______________________ **MWK**<br>☐ 2 = Yes, paid in-kind (specify what): ______________________<br>☐ 3 = No
68	If you work for a wage, did your (or the patient's) employer or boss pay for the days you were <u>sick but did not come to work</u>? *For example, if the patient has sick leave.*	☐ 1 = Yes<br>☐ 2 = No → **skip to 70**<br>☐ 9 = Don't know → **skip to 70**
69	How many sick days did your (or the patient's) employer or boss pay you for?	☐☐ Days<br>☐ 66 = Patient does not work ☐ 99 = Don't know
70	In total, how many days of income did you (or the patient) lose because of this illness <u>since it started</u>?	☐☐ Days<br>☐ 66 = Patient does not work ☐ 99 = Don't know

SECTION E: Indirect Costs: Caretakers & Substitute Labor ("Helpers")

<u>SCRIPT</u>: Now, I would like to understand if anyone helped you (or the patient) while you were sick. This includes 2 types of people:

1. Someone who may have been paid to complete your usual tasks for you
2. Someone who had to help take care of you (or the patient) while you were sick

Sometimes, this is the same person.

71	Were you (or the patient) so sick with cholera that someone had to help you (or the patient) in any way?<br>*This includes helpers who had to skip their own usual work or activities to help you.*	☐ 1 = Yes<br>☐ 2 = No → **skip to 85 (SECTION F)**<br>☐ 9 = Don't know → **skip to 85 (SECTION F)**
72	How many people cared for you (or the patient) or did your usual activities for you while you were sick with cholera?	☐☐ People ☐ 99 = Don't know

For peer review only - http://bmjopen.bmj.com/site/about/guidelines.xhtml

Cholera Surveillance In Malawi (CSIMA) Program
NSANJE DISTRICT USE ONLY

Form S.I.#:__________________ **Study label**

(To be filled by Data Management) **N__ __-__ __ __ __**

**(SECTION E CONTINUED)**						
Interviewer: *Please enter responses for the number of people the patient indicated in* ***Question #72****. For example, if the patient responded "3", then lines A, B, and C should be filled out for each question.*						
**Helper number**	73. Patient's relationship with person who helped him/her while sick.<br><br>*Write "hired help" if this is someone who was paid to perform the patient's usual activities, or was hired to provide care for the person.*	74. Is this helper a member of your household?<br><br>**1** = Yes<br>**2** = No<br>**9** = Don't know	75. How old is this helper?<br><br>**1** = Adult (17+)<br>**2** = Teenager (12–16)<br>**3** = Child (<12)<br>**9** = Don't know	76. How many days or hours did s/he help you (or the patient)?<br><br>*Record the number and circle days or hours.*	77. Was this helper paid to help you (or the patient)?<br><br>**1** = Yes<br>**2** = No<br>→ **skip to 79**<br>**9** = Don't know<br>→ **skip to 79**	78. How much was this helper **paid per day** in Malawian kwacha (MWK) to help the patient?
**A**				**DAYS or HOURS**		**MWK**
**B**				**DAYS or HOURS**		**MWK**
**C**				**DAYS or HOURS**		**MWK**
**D**				**DAYS or HOURS**		**MWK**
**E**				**DAYS or HOURS**		**MWK**

For peer review only - http://bmjopen.bmj.com/site/about/guidelines.xhtml

Cholera Surveillance In Malawi (CSIMA) Program
NSANJE DISTRICT USE ONLY

Form S.I.#:_________________ **Study label**

(To be filled by Data Management) N☐☐-☐☐☐☐

Helper number	79. Did this helper perform:<br><br>**1** = <u>All</u> of the patient's activities<br>**2** = <u>Some</u> of the patient's activities<br>**3** = <u>None</u> of the patient's activities<br>**9** = Don't know	80. Did the helper cut back on his/her own usual activities?<br><br>**1** = Yes<br>**2** = No<br>→ **skip to 83**<br>**9** = Don't know<br>→ **skip to 83**	81. How many days did the helper cut back on his/her own duties?<br><br>*Record the number and <u>circle</u> days or hours.*	82. Was the helper able to do:<br><br>**1** = <u>Some</u> of his/her own activities<br>**2** = <u>None</u> of her/her own activities<br>**9** = Don't know	83. What would the helper have been doing if s/he had not been caring for the sick patient?<br><br>*Indicate all that apply.*<br><br>**1** = Going to school<br>**2** = Working for a wage (paid)<br>**3** = Working for self (unpaid)<br>**4** = Attending church<br>**5** = Leisure or play time<br>**6** = Helping parent<br>**7** = Housework<br>**8** = Other (specify)	84. If helper works for a wage, how much is he/she normally **paid per day** for his/her work in Malawian kwacha (MWK)?
**A**			**DAYS or HOURS**			**MWK**
**B**			**DAYS or HOURS**			**MWK**
**C**			**DAYS or HOURS**			**MWK**
**D**			**DAYS or HOURS**			**MWK**
**E**			**DAYS or HOURS**			**MWK**

CHECK for Interviewer

How many helpers did the patient specify in Question #72?

☐☐ People

Please check that the **number of people matches the number of rows** that you have filled out in **Section E** before proceeding to **Section F. Questions #73 through #84** must be filled out for <u>each</u> helper.

Have you verified?

☐ Yes → **continue to Section F**

SECTION F: Financial Impact of Cholera

<u>**SCRIPT**</u>: We are almost done with the interview. There are just a few more questions I would like to ask you to understand how this cholera event impacted your (or the patient's) financial situation.

85	Did you (or the patient) or your household have to borrow money from anyone in order to pay for the cholera treatment?	☐ 1 = Yes<br>☐ 2 = No → **skip to 89 (SECTION G)**	
86	Who did you (or the patient) or your household borrow money from?	☐ 1 = Family member<br>☐ 3 = Church	☐ 2 = Friend<br>☐ 4 = Neighbors

For peer review only - http://bmjopen.bmj.com/site/about/guidelines.xhtml

Cholera Surveillance In Malawi (CSIMA) Program
NSANJE DISTRICT USE ONLY

Form S.I.#:__________________ **Study label**

(To be filled by Data Management) **N☐☐-☐☐☐☐**

	Check all that apply.	☐ 5 = Village chief ☐ 6 = Boss/employer<br>☐ 7 = Social Grouping or Cooperative<br>☐ 8 = Other (specify): ________________________________
87	Did your (or the patient's) household have to sell any items in order to raise money to pay for the cholera treatment?	☐ 1 = Yes<br>☐ 2 = No → **skip to 89 (SECTION G)**
88	What was sold?<br><br>*Check all that apply.*	☐ 1 = Clothing ☐ 2 = Livestock, specify:_____________<br>☐ 3 = Radio ☐ 4 = Furniture<br>☐ 5 = Other (specify): ________________________________

For peer review only

**SECTION G: Conclusion & Scheduling Next Interview**		
**SCRIPT:** Now, I have some final questions about your (the patient's) current health status.		
89	Compared to how you felt before this illness, how is your overall physical wellness today?	☐ 1 = Worse<br>☐ 2 = The same<br>☐ 3 = Better
90	Do you feel that you (or the patient) has fully recovered from cholera?	☐ 1 = Yes → **continue to SECTION H to Conclude Interview**<br>☐ 2 = No → **continue to below script**<br>☐ 3 = Not sure → **continue to below script**
**SCRIPT:** It sounds like you are still feeling sick because of cholera. So, we would like to follow-up in approximately 7 days with another interview to check on your health. For the next interview, you will not need to answer the questions again about your living arrangements, household belongings, and water, sanitation, and hygiene practices.		

For peer review only - http://bmjopen.bmj.com/site/about/guidelines.xhtml

Cholera Surveillance In Malawi (CSIMA) Program
NSANJE DISTRICT USE ONLY

Form S.I.#:__________________ **Study label**

(To be filled by Data Management) N☐☐-☐☐☐☐

During the second interview, we will only ask you questions about any health services you have tried to seek between today and the next interview. The survey will be almost exactly like this one. If you complete that second survey, we will give you a bar of soap for your time and participation.

What is a convenient time for you? We will attempt to contact you at least 1 day in advance to remind you of the interview.

91	Date for second interview	**(DD/MM/YYYY):** ☐☐/☐☐/☐☐☐☐<br>**(HH:MM, 12-hr format):** ☐☐:☐☐ **AM** ☐ **PM** ☐

SCRIPT: At this second interview, I will ask you again to think about any places you have gone to seek additional treatment or care for this episode of cholera.

To help you remember these visits, here is a diary card where you can record basic information about the visit. If you are unable to fill out the card, please let me know. You will still receive a bar of soap at the next interview even if you do not fill out this diary card.

Interviewer: *Show the diary card to the patient and instruct the patient (or someone who can fill the card out on behalf of the patient) on how to fill out the card. Please ensure that they have a pen to fill it out. If they do not, please give them a pen. If it is afternoon when you are finishing the interview, you may want to ask if the patient has visited any place for treatment or care today. If the patient says "**yes**," you may as an example walk the patient (or person filling out card) through the card to begin to fill out today.*

92. Was the patient (or household member) given the diary card to fill out?

☐ Yes ☐ No (specify reason): ________________________________

Section H: Incentive & Thanking Patient

SCRIPT: Thank you very much for taking the time to answer my questions. That concludes our interview. To thank you for your time, here is a bucket that you may use as you like. We hope that it may be helpful for your household.

Section I: For SURVEY FIELD WORKER

I1. Interviewer Name: ________________________________

Interviewer Signature: ________________________________

I2. Date of Interview Completion (DD/MM/YYYY): ☐☐/☐☐/☐☐☐☐

I3. Please indicate any additional interview details:

☐ Interview completed in full
☐ Patient declined interview
☐ Patient not home
☐ Could not verify patient identity
☐ Patient asked to reschedule (fill out below)
☐ Other: ____________________

For peer review only - http://bmjopen.bmj.com/site/about/guidelines.xhtml

Cholera Surveillance In Malawi (CSIMA) Program
NSANJE DISTRICT USE ONLY

Form S.I.#:__________________ **Study label**

(To be filled by Data Management) N☐☐-☐☐☐☐

***** If a second interview has been schedule, please be sure to transfer and record the scheduled interview date from Question #91 to the D-2 Cost-of-Illness Survey form. *****

Section J: Rescheduling Interview

***** If the interview must be rescheduled for any other reason (e.g., person not home, person wants to continue interview at a later date, person does not want to be interviewed today), please also enter a date below. *****

J1. Date of Rescheduled Interview (DD/MM/YYYY): ☐☐/☐☐/☐☐☐☐

J2. ***Interviewer:*** *Please use this section to write any important details about the interview and/or follow-up interviews for this patient that you feel would be important to remember.*

____

____

____

For peer review only

For peer review only - http://bmjopen.bmj.com/site/about/guidelines.xhtml

BMJ Open

The economic impact of cholera in households in rural southern Malawi: a prospective study

Journal:	*BMJ Open*
Manuscript ID	bmjopen-2021-052337.R2
Article Type:	Original research
Date Submitted by the Author:	02-Mar-2022
Complete List of Authors:	Hsiao, Amber; Technische Universität Berlin Fakultät Wirtschaft und Management, Department of Health Care Management<br>Ramani, Enusa; International Vaccine Institute, Policy and Economic Research ; Technische Universität Berlin Fakultät Wirtschaft und Management, Department of Health Care Management<br>Seo, Hye-jin; International Vaccine Institute, Epidemiology, Public Health, Impact<br>Pak, GiDeok; International Vaccine Institute, Biostatistics & Data Management<br>Vuntade, Dan ; University of Malawi the Polytechnic, Department of Environmental Health<br>M'bang'ombe, Maurice; Ministry of Health Malawi<br>Ngwira, Bagrey; University of Malawi the Polytechnic, Department of Environmental Health<br>Quentin, Wilm; Technische Universität Berlin Fakultät Wirtschaft und Management, Department of Health Care Management<br>Marks, Florian ; International Vaccine Institute, Epidemiology, Public Health, Impact; Cambridge Institute of Therapeutic Immunology and Infectious Disease, Cambridge School of Clinical Medicine, Cambridge Biomedical Campus<br>Mogasale, Vittal; International Vaccine Institute, Policy and Economic Research
**Primary Subject Heading**:	Global health
Secondary Subject Heading:	Health economics, Infectious diseases, Health policy
Keywords:	Public health < INFECTIOUS DISEASES, HEALTH ECONOMICS, Health policy < HEALTH SERVICES ADMINISTRATION & MANAGEMENT, Economics < TROPICAL MEDICINE

SCHOLARONE™
Manuscripts

I, the Submitting Author has the right to grant and does grant on behalf of all authors of the Work (as defined in the below author licence), an exclusive licence and/or a non-exclusive licence for contributions from authors who are: i) UK Crown employees; ii) where BMJ has agreed a CC-BY licence shall apply, and/or iii) in accordance with the terms applicable for US Federal Government officers or employees acting as part of their official duties; on a worldwide, perpetual, irrevocable, royalty-free basis to BMJ Publishing Group Ltd ("BMJ") its licensees and where the relevant Journal is co-owned by BMJ to the co-owners of the Journal, to publish the Work in this journal and any other BMJ products and to exploit all rights, as set out in our licence.

The Submitting Author accepts and understands that any supply made under these terms is made by BMJ to the Submitting Author unless you are acting as an employee on behalf of your employer or a postgraduate student of an affiliated institution which is paying any applicable article publishing charge ("APC") for Open Access articles. Where the Submitting Author wishes to make the Work available on an Open Access basis (and intends to pay the relevant APC), the terms of reuse of such Open Access shall be governed by a Creative Commons licence – details of these licences and which Creative Commons licence will apply to this Work are set out in our licence referred to above.

Other than as permitted in any relevant BMJ Author's Self Archiving Policies, I confirm this Work has not been accepted for publication elsewhere, is not being considered for publication elsewhere and does not duplicate material already published. I confirm all authors consent to publication of this Work and authorise the granting of this licence.

For peer review only

The economic impact of cholera in households in rural southern Malawi: a prospective study

Amber Hsiao,[1] Enusa Ramani,[1,2] Hye Jin Seo,[2] Gi Deok Pak[2], Dan Vuntade[3], Maurice M'bang'ombe,[4] Bagrey Ngwira,[3] Wilm Quentin,[1] Florian Marks,[2,5,6] Vittal Mogasale[2*]

[1] Technische Universität Berlin, Department of Health Care Management, Berlin, Germany

[2] International Vaccine Institute, Seoul, Republic of Korea

[3] University of Malawi, Department of Community Health, Blantyre, Malawi

[4] Ministry of Health Malawi, Lilongwe, Malawi

[5] Cambridge Institute of Therapeutic Immunology and Infectious Disease, University of Cambridge School of Clinical Medicine, Cambridge Biomedical Campus, Cambridge, UK, CB20AW

[6] University of Antananarivo, Antananarivo, Madagascar

***Corresponding Author:**

Vittal Mogasale, MBBS, MPH, PhD

International Vaccine Institute, Policy & Economic Research Department,

SNU Research Park, 1 Gwanak-ro, Gwanak-gu Seoul 08826. The Republic of Korea.

T +82 2 881 1379; F + 82 2 872 2803

Email: vmogasale@gmail.com; vmogasale@ivi.int

Word count (excluding title page, abstract, references, tables/figures)

For peer review only - http://bmjopen.bmj.com/site/about/guidelines.xhtml

ABSTRACT

Introduction. Cholera remains a significant contributor to diarrheal illness, especially in sub-Saharan Africa. Few studies have estimated the cost of illness (COI) of cholera in Malawi, a cholera-endemic country. The present study estimated the COI of cholera in Nsanje, southern Malawi, as part of the Cholera Surveillance in Malawi (CSIMA) program following a mass cholera vaccination campaign in 2015.

Methods: Patients ≥12 months of age who were recruited as part of CSIMA were invited to participate in the COI survey. The COI tool captured household components of economic burden, including direct medical and non-medical costs, and indirect lost productivity costs.

Results: Between April 2016 to March 2020, 40 cholera cases were enrolled in the study, all of whom participated in the COI survey. Only two patients had any direct medical costs and five patients reported lost wages due to illness. The COI per patient was $14.34 (in 2020 USD), more than half of which was from direct non-medical costs from food, water, and transportation to the health center.

Conclusion: For the majority of Malawians who struggle to subsist on less than $2 a day, the COI of cholera represents a significant cost burden to families. While cholera treatment is provided for free in government-run health centers, additional investments in cholera control and prevention at the community level and financial support beyond direct medical costs may be necessary to alleviate the economic burden of cholera on households in southern Malawi.

For peer review only - http://bmjopen.bmj.com/site/about/guidelines.xhtml

49 **Strengths and limitations of this study**

50 • We recruited cholera-confirmed patients at the time they first sought care and interviewed
51 them regarding costs incurred due to illness within 7 days following their initial health
52 facility visit, which limited recall bias.

53 • The sample size was relatively small, which limits the generalizability of the findings.

54 • The cost of illness estimate is likely to be an underestimate as it only includes out-of-
55 pocket expenditures of patients who sought care.

For peer review only

For peer review only - http://bmjopen.bmj.com/site/about/guidelines.xhtml

INTRODUCTION

Cholera is an infection of the intestine caused by the bacterium *Vibrio cholerae* that leads to acute, rapidly-dehydrating diarrheal disease. Annually, an estimated 2.86 million cases of cholera occur in cholera endemic countries that result in 95,000 deaths.[1] A recent study estimated that cholera is especially pervasive throughout sub-Saharan Africa (sSA) where the human and economic burden of cholera is greatest, with more than 200 million people living in areas where cholera is present. Of these 200 million, 87 million live in areas with high incidence, defined as >1 case per 1,000 people per year.[2]

While improvements to water, sanitation, and hygiene (WASH) are the best long-term solution for preventing more diarrheal diseases, these measures remain unavailable to large proportions of people in sub-Saharan Africa, especially among those living in flood-prone rural areas. The availability of low-cost inactivated oral cholera vaccines (OCV) since 2011 has significantly changed the landscape and provided a cost-effective short-to-middle-term approach for cholera prevention and control.[3] A number of mass vaccination campaigns—many of which have taken place in sSA—have demonstrated that it can be successfully administered in a variety of settings, including high-risk settings to prevent an impending outbreak, or to quickly halt the transmission of an ongoing outbreak.[4 5]

In Malawi specifically, cholera has been reported since the 1970s and is endemic to the country.[6-8] The extent of the cholera burden in the country has not been well documented due to the lack of sustainable diagnostic and surveillance capacity.

For peer review only - http://bmjopen.bmj.com/site/about/guidelines.xhtml

One of the worst cholera outbreaks to date occurred in January 2015 following the nation's worst floods in history, which affected more than 1.1 million people in the Southern Region and displaced at least 230,000 people from their homes.[9] Within a month of the flood, cholera cases were being reported in Nsanje and Chikwawa, two of the worst-hit districts in southern Malawi. The flooding led to a multi-stakeholder response to coordinate a reactive OCV mass vaccination campaign in the region.[10] Due to the lack of diagnostic capacity, cholera cases were being diagnosed based on clinical presentation as per the World Health Organization (WHO) and national guidelines.[11]

The OCV mass vaccination campaign was then implemented primarily in Nsanje, which was identified as having the highest burden of cholera based on prior years' data provided by the Malawian Ministry of Health (MOH). From March 30, 2015, to April 23, 2015, the International Vaccine Institute (IVI), MOH, WHO, and John Snow, Inc. coordinated a reactive mass vaccination campaign that targeted 160,000 people (320,000 total doses of the OCV Shanchol® (Shantha Biotechnics Pvt., Ltd., India) over 1 year of age. Those living in high-risk areas of Nsanje—namely, those living in internally displaced camps and surrounding villages—were the primary targets.[10]

In the course of a 4-year effort to strengthen cholera surveillance capacity and infrastructure in Nsanje following the mass vaccination campaign, we investigated the cost-of-illness (COI) of cholera for patients who sought care. Only one study to date has reported on the cost-of-illness from cholera in southeastern Malawi (Lake Chilwa in the districts of Machinga and Zomba).[12]

For peer review only - http://bmjopen.bmj.com/site/about/guidelines.xhtml

The objective of this study is to provide additional field data on the economic burden of cholera in Malawi from a household perspective.

METHODS

As part of the Cholera Surveillance in Malawi (CSIMA) program, cholera surveillance was conducted across 22 Nsanje (population >292,000) and 18 Chikwawa (population >533,000) healthcare facilities from April 5, 2016, to March 31, 2020. IVI staff trained health surveillance assistants (HSAs) and health workers at all Nsanje and Chikwawa facilities to identify patients who sought care for acute watery diarrhea. HSAs are community health facilitators that live in the area served by the local healthcare facility who are involved in activities such as WASH and general health promotion.

Patients who met the inclusion criteria (who were eligible to receive OCV based being at least 12 months of age at the start of the vaccination campaign, and presented to an Nsanje health facility with acute watery diarrhea indicative of cholera as defined by the WHO[11]) were invited to participate in the diarrheal surveillance study. Participants who provided informed written consent provided a stool specimen to perform a rapid diagnostic test (RDT) for cholera (Crystal® VC RDT; Arkray Healthcare Pvt., India; previously Span Diagnostics, Surat, India) and/or a stool sample for culture confirmation at the national reference laboratory in Lilongwe. The use of RDTs has been accepted for use by the Malawi MOH, and there is documented experience with this test kit in other settings.[13, 14] While the use of the RDT is not intended to be a substitute for stool culture, the use of the kit in this setting ensured that one would not miss any

For peer review only - http://bmjopen.bmj.com/site/about/guidelines.xhtml

potential cholera cases during the early surveillance period while logistics for stool transportation and culture were being set-up.

Between April 6, 2016, and December 12, 2017, culture-confirmed and RDT-positive cholera cases were recruited for the overall study, as well as the COI survey component. However, due to low recruitment numbers of RDT- and/or culture-confirmed cholera cases, in part due to limited availability of RDT kits and stool collection logistical challenges, the inclusion criteria were expanded from December 12, 2017, through March 31, 2020, to include clinically-diagnosed cholera cases in an outbreak situation to avoid missing potential cases.

While cholera vaccines were administered to residents in both Nsanje and Chikwawa, the majority of doses were administered to residents of Nsanje. Thus, only patients recruited from Nsanje health centers were invited for the COI portion of the study. The COI from a household perspective was assessed for patients who provided written informed consent (regardless of vaccination status). The COI tool (supplementary material) was developed based on a tool that IVI had previously used for other COI studies, with further refinements using the guidelines for cost collection in cholera studies provided by the WHO.[15, 16] The survey tool captured household components of economic burden, divided further into direct medical costs (DMC), direct non-medical costs (DNMC), and indirect costs (IC) (as done in the previous studies[17]), with the payer being the patients themselves or caregivers/helpers of the patient (e.g., relatives). Experienced interviewers were trained on how to administer the survey and all survey responses were validated by the University of Malawi Polytechnic and MOH collaborators to ensure they were appropriate for the local context.

For peer review only - http://bmjopen.bmj.com/site/about/guidelines.xhtml

A series of a maximum of three interviews were planned for the COI assessment, with day 0 (i.e., day of diagnosis) being the day of the patient's health facility visit: at day 0–7, day 13–15 (beyond the mean duration of an episode of cholera illness), and day 20–22. To minimize recall bias, the first interview took place as soon as cholera was confirmed either by a test or a clinician, with a maximum of 7 days following the first healthcare facility visit. If a patient preferred to be interviewed while at the healthcare facility, the trained interviewer conducted the first COI interview at the time of the initial outpatient or inpatient visit. Patients who were not feeling well enough were contacted by an HSA to schedule a future interview time. Trained interviewers would then visit the patient's home at the scheduled time to conduct the first interview. At the end of each COI assessment, patients were asked whether they felt they had fully recovered from cholera. If the patient reported still being ill, the next interview was scheduled; otherwise, no additional interviews were conducted.

At each of the interviews, patients (or parent/guardian) were asked how much money they spent during each of their visits to a healthcare facility or other treatment locations (e.g., a traditional healer) since becoming ill, including any visits that may have occurred prior to the study enrollment date. DMCs included out-of-pocket payments for services, prescription drugs, and any other health facility fees. DNMCs included transportation and food expenses incurred by the patient and/or caregivers who went to the health facility with the patient.

Finally, IC accounted for the patient's and/or caregivers' productivity losses incurred due to lost wages. We calculated productivity losses using three scenarios based upon (1) self-reported wage losses, (2) minimum wage per day for all workers between 14–50 years of age (the legal

For peer review only - http://bmjopen.bmj.com/site/about/guidelines.xhtml

minimum and old-age pension eligibility age[18]) who did not report wage losses, and (3) minimum wage per day for all patients >5 years of age who did not report wage losses, given evidence from Africa that children are often involved in economic activities that add to a family's income.[19]

In all scenarios, if patients and/or caregivers reported wage losses, these were calculated based on self-reported daily lost productivity (half or full days of work lost due to taking care of the ill patient) and daily wages. For patients and caregivers who did not report wage losses (i.e., scenarios 2 and 3), we applied the minimum wage of 1,346.16 Malawian kwacha (MWK) per day ($1.83 USD/day as of January 1, 2020) to account for the monetary value of productivity losses.[20] Productivity losses were not calculated for caregivers who indicated that they did not cut back on their usual activities had they not been caring for the patient. They were also not calculated for caregivers who reported no income lost (e.g., a caregiver who is dedicated to unpaid housework).

Costs were collected in Malawian kwacha (MWK), inflated to 2020 MWK using World Bank inflation data,[21] and converted to 2020 US$ (736.5803 MWK=$1 US on March 31, 2020).[22] All analyses were conducted in Stata/IC 13.1 (College Station, TX) and Microsoft Excel.

Ethics approval

This study was approved by the National Health Sciences Research Committee (NHSRC) of the Ministry of Health in Malawi (protocol #16/2/1547; approval #1547) on March 29, 2016. IVI's Institutional Review Board (IRB) approved the study on April 6, 2016. All participants (or a

For peer review only - http://bmjopen.bmj.com/site/about/guidelines.xhtml

parent/guardian) provided written informed consent prior to interviews. For patients aged <12 years, consent was provided by the parent/guardian, and in these cases, the patient/caregiver provided the interview responses. For patients aged 12–17 years, ascent was provided by both the parent/guardian and child. Adults aged ≥18 years provided consent directly. The IVI IRB and the NHSRC approved the modification to expand to inclusion of clinically-diagnosed cases on May 31, 2017, and July 31, 2017, respectively. Training of all HSAs on the modification occurred on December 12 and 13, 2017, and the data collection continued until March 31, 2020.

Patient and Public Involvement

Patients including their caretakers were involved in this study as participants in the cost of illness interviews. Costs related to cholera illness were collected from the participants after obtaining informed consent. The public was not involved in this study. Patients and the public were not involved in study development, defining the research question and study design, recruitment, and the conduct of the study. Early findings of the study were disseminated through a workshop attended by country policymakers, health managers, and health facility staff.

RESULTS

During our surveillance period, 2,201 individuals met the diarrheal illness criteria for stool collection and testing for cholera in Nsanje. Of these patients, 40 (1.8%) were identified as cholera cases and were recruited for the surveillance study. On the day of their health facility visit, all 40 patients also consented for COI interview. Table 1 summarizes selected demographic characteristics, as well as OCV vaccination statuses, of all cases.

For peer review only

For peer review only - http://bmjopen.bmj.com/site/about/guidelines.xhtml

Of the 40 cases, 31 (78%) were seen in an outpatient setting. Duration of illness was 5 days (min–max: 2–17 days) in the outpatient setting and 8 days (min–max: 2–19 days) in the inpatient setting. Most patients (n=34) had fully recovered by the time the first interview occurred (scheduled within 7 days of recruitment day 0); 6 patients had a second interview (at day 13–15) and none required a third interview. A higher proportion of inpatient cases were males, whereas outpatient cases were majority female. Of the 40 cases, 4 were identified clinically, 9 using RDT, and 27 using culture confirmation. A larger proportion of children were seen in the inpatient setting, compared with outpatient.

The direct and indirect cost of cholera per patient was $14.34 when averaged across all patients, with the largest proportion of costs (57.9%) being from direct non-medical costs (food, water, and transportation) (Table 2). Direct medical costs for drugs and consumables were zero for nearly all patients; only 2 patients seen in the inpatient setting had any drug costs of $11.17 (8,229 MWK) and $1.19 (875 MWK) each, both of whom were seen at a private Christian Health Association of Malawi (CHAM) facility; these two patient costs increased the overall average across the cohort to $0.31.

Patient and caregiver food costs were $0.95 and $1.61 on average, respectively. Roundtrip transportation costs were relatively lower for patients at $1.84, compared to caregiver transportation costs at $3.91. Transportation time on average per patient was 112 minutes roundtrip (min, max: 10, 360).

For peer review only - http://bmjopen.bmj.com/site/about/guidelines.xhtml

We also performed various scenario analyses for indirect costs (IC) (Table 3) where costs were only averaged across patients and caregivers with non-zero productivity losses in each scenario. Eight patients were excluded from wage loss calculations due to missing age or birthdate. Thirty-four patients had at least one caregiver provide care during their illness, with a total of 53 caregivers across all patients. Caregivers only reported whether they were children or adults; (i.e., exact ages or birthdates were not ascertained); all caregivers reported being adults who we assumed were of working age.

In scenario 1, productivity losses (lost wages) due to being sick for the duration of illness were \$10.60 on average (n=5) for patients who reported wage losses, though one patient reported receiving paid sick leave for this time (\$10.89 USD), which would reduce the average to \$10.53 (n=4). Only 15 caregivers (who cared for 8 patients) reported \$6.36 on average in lost wages due to missing work. In scenario 2 where the minimum wage was assumed for all patients (n=11) and caregivers (n=45) of working age, productivity losses were \$8.83 for patients and \$3.93 for caregivers on average. In scenario 3, where the minimum wage was assumed for all patients >5 years of age (n=19), productivity losses were \$9.78 for patients on average. In this scenario analysis, there were 2 patients aged 5–14 years (\$14.16 on average) and 5 patients aged ≥60 years (\$8.59 on average). Caregiver costs in scenarios 2 and 3 were the same (\$3.94) since all caregivers were adults.

Half of all patients (n=20), and two-thirds of inpatients (n=6), reported having to borrow money from someone to pay for health care, food, or transportation as a result of having cholera (Table 4), but most did not have to sell personal belongings to pay for care. Experiencing at least some

For peer review only - http://bmjopen.bmj.com/site/about/guidelines.xhtml

food insecurity in the past year (defined as going without food at least once in the past 3 months) was an issue for a large proportion of patients (n=28).

DISCUSSION

Our study estimated patient and caregiver costs associated with seeking healthcare for the treatment of severe diarrheal illness diagnosed as cholera in Nsanje. We had few diarrheal cases overall. The cost of cholera illness per patient was $14.34, with the largest proportion of costs being from direct non-medical costs (food, water, and transportation) ($8.30), followed by indirect productivity losses ($5.73) in our primary analysis where costs were averaged across all patients in our cohort.

The majority of our patients were not admitted during their visit to the health center. Patients who sought care in the outpatient setting were symptomatic an average of 1.4 days prior to seeking care. Patients who were admitted were sick for an average of 2.8 days prior to seeking care and had an inpatient stay of 2 days. Our patient population was slightly skewed toward females and of those whose age was reported, most patients were adults aged 18 and older. The majority of patients were not vaccinated during the OCV campaign in 2015.

Overall, only two cholera patients had any DMCs; one was seen in the outpatient setting (875 MWK, or $1.19) and the other was admitted to a private CHAM facility (8,229 MWK, or $11.17). This finding was expected, as cholera treatment is provided free of charge in Malawi. On average, patients and their caregivers spent $8.61 in direct costs ($0.31 in medical costs and $8.30 in non-medical costs, including those with zero costs), almost all of which was due to food

For peer review only - http://bmjopen.bmj.com/site/about/guidelines.xhtml

and/or water and transportation costs. In our scenario analysis when only those with self-reported wages are considered, only 5 cholera patients (all outpatient cases) reported productivity losses of $8.83 on average, and 15 caregivers of patients reported wage losses, averaging $3.93. In our scenario analysis where we applied the minimum wage to all patients and caregivers who theoretically could be contributing to economic activities (i.e., household work), indirect productivity losses were as high as $9.78 per patient and $3.94 per caregiver. This latter scenario may overestimate the number of individuals who experience productivity losses and the actual economic impact, though the average productivity losses ($13.72) were lower than in the self-reported wage scenario ($16.96).

The overall costs found in our study are substantially less than one study previously reported in rural Malawi.[12] Ilboudo et al. reported household costs of $65.60 in US$ 2016 ($106.16 in US$ 2020).They reported a much larger proportion of hospitalized cases: their study sampled 100 cases (which estimated 9% of the overall caseload from the cholera outbreak), 90 of whom were hospitalized, whereas we only had 7 hospitalized cases (17.5% of total cases). Transportation costs in our study were also lower for both patients and caregivers compared with their study, which may be attributable to differences in transportation methods and travel distances to health centers.

The largest differences in costs between our study and Ilboudo et al. were in food and lost productivity costs, likely also driven by the fact that their study had more hospitalized cases that required more expenses over several days. Their reported indirect costs of patient's productivity were a larger portion of overall costs (35.1% compared to our 9.2%), which may be attributed to

For peer review only - http://bmjopen.bmj.com/site/about/guidelines.xhtml

their study cohort having longer inpatient stays. The caregiver lost productivity costs as a proportion of overall costs were lower in their study (23.5% compared to our 30.7%). Differences in productivity costs may be attributed to several reasons, such as differences in wages in Nsanje versus Machinga and Zomba districts, unemployment rates, and other socioeconomic factors. The differences may also be attributed to the severity of illness, potentially attenuated by vaccination. Ilboudo et al. assessed costs of illness from patients who had cholera in the ±1 week prior to and after the OCV vaccination campaign that was underway (which followed the cholera outbreak). Thus, the proximity in timing to the outbreak may have meant that few individuals had any immunity to cholera and may have experienced more severe symptoms, whereas in our population, OCV coverage and immunity was likely higher. Given the relatively small sample size in both studies, however, more data is needed from the region to understand why these costs differed more than other cost components.

We also assessed financial and food insecurity for cases. Half of our cases had to borrow money from someone to pay for costs associated with their cholera illness and treatment, which is similar to the proportion reported by Ilboudo et al. A large proportion of our cases also faced food insecurity at some point in the prior year.

Our study had several limitations. One limitation was the late start of cholera surveillance (April 2016) relative to the cholera outbreak (~February 2015), in response to which a vaccination campaign was conducted (March/April 2015). It is possible that some post vaccination campaign cholera cases may have occurred in the year prior to the start of cholera surveillance, particularly during the November 2015 to April 2016 rainy season; these cases would not have been included

For peer review only - http://bmjopen.bmj.com/site/about/guidelines.xhtml

in this study. The study was also not able to collect COI data from Chikwawa residents and infants <12 months, which may have provided different cost estimates. Given the small number of cholera cases, even after expanding the case criteria to clinically-diagnosed cases (of which there were only 4), it may not be representative of the general Nsanje population, particularly those who were sick and did not seek care. It is also possible that patients may have shown up for care but were undiagnosed or misdiagnosed for reasons such as diagnostic sensitivity. While we were unable to assess any patterns or variation in the use of the three testing methods (RDT, culture, or clinically-identified) by the health center, it is possible that our recruited patient cohort is biased toward health centers that were better equipped to test, recruit, and treat patients. For example, many of the more remote health centers in Nsanje lacked toilets or latrines for proper stool collection and have limited access to electricity to ensure the viability of stool samples, which may have limited the sensitivity of the stool culture result. Apart from the logistical challenges of recruitment, the area also has a high number of migrants from Mozambique; migrants may be less likely to seek care due to several reasons, including language barriers, discrimination due to cultural differences, and fear of deportation.[23]

Health facility costs were also not included in our costing, which may be a significant portion of the total costs as most patients reported $0 in direct medical costs. A recent study in rural Malawi estimated that the average treatment cost to a health facility for a hospitalized cholera case was $59.70 in US$ 2016 ($96.61 in US$ 2020).[12] A separate study on cholera treatment costs at the facility level at this study site has estimated an economic cost of $41.86 in US$ 2018 ($50.79 in US$ 2020) and a financial cost of $25.43 in US$ 2018 ($30.85 in US$ 2020).[24] Finally, there was the potential for recall bias, especially for patients who may have had costs prior to the

For peer review only - http://bmjopen.bmj.com/site/about/guidelines.xhtml

initial visit to the health center from which they were recruited for the study. For costs incurred from the day of enrollment onward, we attempted to minimize the potential for recall bias by following up first within a week of the initial visit.

Despite the limitations, our study provides one of the few cost of illness studies of cholera illness in sub-Saharan Africa from the household perspective. More studies are needed to more fully evaluate the cholera costs of illness in Malawi. Currently, spending on health accounts for less than 10% of country revenues—below the 15% target set by the Abuja Declaration to which Malawi is a signatory.[25] Health care financing in Malawi is also unpredictable and unsustainable given the country's reliance on development partners' contributions that account for nearly two-thirds of total health expenditure.[26] Our study supports the need for further spending in the health sector; for example, building and staffing additional health centers that reduce the amount of time patients have to travel could reduce the cost burden on patients.

While the study was not designed to assess vaccine effectiveness, our surveillance efforts may also be a positive indication that the cholera vaccine may be associated with a reduction in the burden of cholera in the middle-term, as most of our cases were unvaccinated (while approximately half of the Nsanje population received the cholera vaccine). Since this study was conducted, in recognition of the high incidence of cholera and the need for additional tools to address the burden, the government of Malawi integrated OCVs into its national cholera control plan in 2017,[27] and there have been several OCV campaigns implemented since.[10, 28] Alternatively, given that cholera often occurs cyclically, it is possible that the time period during which surveillance occurred was a low or no cholera transmission interval. Ultimately, investing

For peer review only - http://bmjopen.bmj.com/site/about/guidelines.xhtml

more heavily in WASH infrastructure would likely be the most effective, though more costly, solution in the long-run to avert cholera.

In a country where the gross domestic product per capita per day is less than $2 ($625 annually in 2020 USD), a family who has to spend $14 on average for cholera illness will very likely have to make financial sacrifices to access treatment. Even though the direct medical costs associated with cholera care are low, the patient (who is willing and able to go to the health center) must bear a high burden of direct non-medical and indirect lost productivity costs. For those who do not have the means of even going to a health facility to seek care, the consequences are likely even more severe; for example, the death of a family member whose wages supported the family has significant long-term impacts beyond a single cholera episode.

In conclusion, we find that households face a significant cost burden when one falls sick with cholera. These costs are significantly high to warrant additional investments in the health system to avert future cases of cholera.

AUTHORS CONTRIBUTIONS

AH and VM conceptualized the study, led the protocol development, field training, and survey implementation in collaboration with HJS, GDP, DV, MM, BN, FM, & ER. Data collection was supervised by DV, MM, and BN, and monitored by GDP, AH, VM, ER, and HJS. Data analysis and interpretation were done by AH supported by VM and WQ. The manuscript was written by

For peer review only - http://bmjopen.bmj.com/site/about/guidelines.xhtml

AH, supported by VM and WQ. All authors have reviewed, edited, and approved the final manuscript.

COMPETING INTERESTS

None declared.

DATA SHARING

All data relevant to the study are included in the article or uploaded as supplementary information

FUNDING

This research was conducted by International Vaccine Institute and funded by Bill & Melinda Gates Foundation [OPP38590] and the UK Foreign, Commonwealth and Development Office and Wellcome [214662/Z/18/Z]. The International Vaccine Institute acknowledges its donors including the Republic of Korea, Swedish International Development Cooperation Agency and Republic of India.

For peer review only - http://bmjopen.bmj.com/site/about/guidelines.xhtml

REFERENCES

1. Ali M, Nelson AR, Lopez AL, et al. Updated global burden of cholera in endemic countries. *PLoS neglected tropical diseases* 2015;9(6):e0003832. doi: 10.1371/journal.pntd.0003832 [published Online First: 2015/06/05]
2. Lessler J, Moore SM, Luquero FJ, et al. Mapping the burden of cholera in sub-Saharan Africa and implications for control: an analysis of data across geographical scales. *Lancet (London, England)* 2018;391(10133):1908-15. doi: 10.1016/s0140-6736(17)33050-7 [published Online First: 2018/03/06]
3. Hsiao A, Hall AH, Mogasale V, et al. The health economics of cholera: A systematic review. *Vaccine* 2018;36(30):4404-24. doi: 10.1016/j.vaccine.2018.05.120 [published Online First: 2018/06/17]
4. Hsiao A, Desai SN, Mogasale V, et al. Lessons learnt from 12 oral cholera vaccine campaigns in resource-poor settings. *Bulletin of the World Health Organization* 2017;95(4):303-12. doi: 10.2471/blt.16.175166 [published Online First: 2017/05/10]
5. Pezzoli L. Global oral cholera vaccine use, 2013-2018. *Vaccine* 2019 doi: 10.1016/j.vaccine.2019.08.086 [published Online First: 2019/09/15]
6. Msyamboza KP, Kagoli M, M'Bang'ombe M, et al. Cholera outbreaks in Malawi in 1998-2012: social and cultural challenges in prevention and control. *Journal of infection in developing countries* 2014;8(6):720-6. doi: 10.3855/jidc.3506 [published Online First: 2014/06/12]
7. Swerdlow DL, Malenga G, Begkoyian G, et al. Epidemic cholera among refugees in Malawi, Africa: treatment and transmission. *Epidemiology and infection* 1997;118(3):207-14. [published Online First: 1997/06/01]
8. World Health Organization. Cholera Country Profile: Malawi 2010.
9. Government of Malawi. Malawi 2015 Floods Post Disaster Needs Assessment Report, March 2015.
10. Msyamboza KP, M'Bang'ombe M, Hausi H, et al. Feasibility and acceptability of oral cholera vaccine mass vaccination campaign in response to an outbreak and floods in Malawi. *The Pan African medical journal* 2016;23:203. doi: 10.11604/pamj.2016.23.203.8346 [published Online First: 2016/06/28]
11. World Health Organization. Ending Cholera—A Global Roadmap to 2030, 2017.
12. Ilboudo PG, Huang XX, Ngwira B, et al. Cost-of-illness of cholera to households and health facilities in rural Malawi. *PloS one* 2017;12(9):e0185041. doi: 10.1371/journal.pone.0185041 [published Online First: 2017/09/22]
13. Mukherjee P, Ghosh S, Ramamurthy T, et al. Evaluation of a rapid immunochromatographic dipstick kit for diagnosis of cholera emphasizes its outbreak utility. *Jpn J Infect Dis* 2010;63(4):234-38.
14. Sinha A, Sengupta S, Ghosh S, et al. Evaluation of a rapid dipstick test for identifying cholera cases during the outbreak. *The Indian journal of medical research* 2012;135(4):523-8. [published Online First: 2012/06/06]
15. World Health Organization, Department of Immunization Vaccines and Biologicals. Guidelines for estimating the economic burden of diarrhoeal disease, with focus on assessing the costs of rotavirus diarrhoea. Geneva, Switzerland, July 2005.
16. Poulos C, Riewpaiboon A, Stewart JF, et al. Costs of illness due to endemic cholera. *Epidemiology and infection* 2012;140(3):500-09.

For peer review only

For peer review only - http://bmjopen.bmj.com/site/about/guidelines.xhtml

17. Lee J-S, Mogasale V, Lim JK, et al. A multi-country study of the economic burden of dengue fever based on patient-specific field surveys in Burkina Faso, Kenya, and Cambodia. *PLoS neglected tropical diseases* 2019;13(2):e0007164. doi: 10.1371/journal.pntd.0007164

18. US Social Security Office of Retirement and Disability Policy. Social Security Programs Throughout the World: Africa, 2019 n.d. [Available from: Available at https://www.ssa.gov/policy/docs/progdesc/ssptw/2018-2019/africa/malawi.html.

19. Chima RI, Goodman CA, Mills A. The economic impact of malaria in Africa: a critical review of the evidence. *Health policy (Amsterdam, Netherlands)* 2003;63(1):17-36. doi: 10.1016/s0168-8510(02)00036-2 [published Online First: 2002/12/07]

20. WageIndicator.org; Minimum Wage – Malawi (January 1, 2020). https://wageindicator.org/salary/minimum-wage/malawi/archive/20200101 Accessed 1 February 2022.

21. World Bank. “Inflation, consumer prices (annual %) – Malawi. https://data.worldbank.org/indicator/FP.CPI.TOTL.ZG?locations=MW Accessed 31 January 2022.

22. Reserve Bank of Malawi. Exchange Rates. https://www.rbm.mw/Statistics/MajorRates Accessed 31 January 2022.

23. Legido-Quigley H, Pocock N, Tan ST, Pajin L, Suphanchaimat R, Wickramage K, McKee M, Pottie K. Healthcare is not universal if undocumented migrants are excluded. BMJ 2019; 366:14160.

24. Morgan W, A L, Hutubessy R, et al. Cost of treatment of cholera in rural Malawi Health facilities. (Under Preparation)

25. World Health Organization. The Abuja Declaration: Ten Years On 2011 [Available from: Available at http://www.who.int/healthsystems/publications/abuja_declaration/en/.

26. Ministry of Health. Malawi Health Sector Strategic Plan II, 2017-2022. April 2017. https://extranet.who.int/countryplanningcycles/sites/default/files/planning_cycle_repository/malawi/health_sector_strategic_plan_ii_030417_smt_dps.pdf accessed 20 JAN 2022.

27. M'Bangombe M, Pezzoli L, Reeder B, et al. Oral cholera vaccine in cholera prevention and control, Malawi. *Bulletin of the World Health Organization* 2018;96(6):428-35. doi: 10.2471/blt.17.207175 [published Online First: 2018/06/16]

28. Sauvageot D, Saussier C, Gobeze A, et al. Oral cholera vaccine coverage in hard-to-reach fishermen communities after two mass Campaigns, Malawi, 2016. *Vaccine* 2017;35(38):5194-200. doi: 10.1016/j.vaccine.2017.07.104 [published Online First: 2017/08/15]

For peer review only

TABLES

Table 1. Demographic and medical characteristics of cholera cases in Nsanje District, Malawi, 2016–2020

	Outpatient n (%)	Inpatient n (%)	Total n (%)
**Total**	31 (78)	9 (22)	40 (100)
**Sex**			
Male	9 (29)	6 (67)	15 (38)
Female	17 (55)	3 (33)	20 (50)
Unknown/Missing	5 (16)	0 (0)	5 (13)
**Age**			
<5 years	10 (32)	4 (44)	14 (35)
≥5 years to <18 years	2 (6)	1 (11)	3 (8)
≥18 years to <35 years	9 (29)	0 (0)	9 (23)
≥35 years	3 (10)	3 (33)	6 (15)
Unknown/Missing	7 (23)	1 (11)	8 (20)
**Level of education**			
Never went to school	13 (42)	3 (33)	16 (40)
Preschool	2 (6)	0 (0)	2 (5)
Completed primary	10 (32)	3 (33)	13 (33)
Completed secondary	2 (6)	0 (0)	2 (5)
Unknown/Missing	4 (13)	3 (33)	7 (18)
**Employment status**			
Works for a wage	5 (16)	0 (0)	5 (13)
Does not work for a wage	26 (84)	9 (100)	35 (88)
**Average inpatient stay, days (min-max)**	—	2 (1–4)	—
**Average number of symptomatic days prior to seeking medical care (min–max)**	1.4 (0–4)	2.8 (1–8)	1.7 (0–8)
**Average number of days sick with cholera**	5 (2–17)	8 (2–19)	6 (2–19)
**Method of transportation for patients to health center**			
Walked	9 (29)	1 (11)	10 (25)
Biked	12 (39)	5 (56)	17 (43)
motorcycle	3 (10)	1 (11)	4 (10)
Minibus	5 (16)	1 (11)	6 (15)
private car	1 (3)	0 (0)	1 (3)
hired canoe	1 (3)	0 (0)	1 (3)
ambulance (from MOH)	0 (0)	1 (11)	1 (3)
**Vaccination status (OCV in 2015)**			
Not vaccinated	16 (52)	5 (56)	21 (53)

For peer review only - http://bmjopen.bmj.com/site/about/guidelines.xhtml

One dose	5	(16)	1	(11)	6	(15)
Two doses	6	(19)	0	(0)	6	(15)
Unknown/Missing	4	(13)	3	(33)	7	(18)
**Cholera confirmation**						
Rapid diagnostic test	5	(56)	4	(44)	9	(22)
Lab culture confirmation	25	(93)	2	(7)	27	(68)
Clinically-identified	1	(25)	3	(75)	4	(10)

503 * Sum of percentages may exceed 100 due to rounding.

504

For peer review only

For peer review only - http://bmjopen.bmj.com/site/about/guidelines.xhtml

Table 2. Mean direct and indirect costs (MWK and USD 2020) for cholera cases in Nsanje District, Malawi, 2016–2020* (averaged across all 40 patients)

	Inpatient (n=9)		Outpatient (n=31)		Total costs (n=40)		
	2020 MWK (min, max)	2020 USD (min, max)	2020 MWK (min, max)	2020 USD (min, max)	2020 MWK	2020 USD	% of total
**Direct, medical costs**	**97**	**0.13**	**265**	**0.36**	**228**	**0.31**	**2.2%**
Drugs and consumables[1]	97 (0–875)	0.13 (0–1.19)	265 (0–8,229)	0.36 (0–11.17)	228	0.31	2.6%
**Direct, non-medical costs**	**9,703**	**13.17**	**5,073**	**6.89**	**6,115**	**8.30**	**57.9%**
Patient food and/or water	505 (0–1,353)	0.69 (0–1.84)	753 (0–6,148)	1.02 (0–8.35)	697	0.95	6.6%
Patient transportation (roundtrip)	2,007 (0–8,750)	2.73 (0–11.88)	1,161 (0–9,836)	1.58 (0–13.35)	1,352	1.84	12.8%
Caregiver food and/or water	1,332 (0–2,734)	1.81 (0–3.71)	1,143 (0–12,686)	1.55 (0–17.22)	1,186	1.61	11.2%
Caregiver transportation (roundtrip)	5,858 (0–13,525)	7.95 (0–18.36)	2,016 (0–8,346)	2.74 (0–11.33)	2,880	3.91	27.3%
**Indirect (productivity losses)[2]**	**489**	**0.66**	**5,304**	**7.20**	**4,221**	**5.73**	**40.0%**
Patient	0 (0–0)	0 (0–0)	1,259 (0–12,519)	1.71 (0–17.00)	976	1.32	9.2%
Caregivers	489 (0–2,213)	0.66 (0–3.00)	4,045 (0–70,526)	5.49 (0–95.75)	3,245	4.41	.0.7%
**Total mean costs[3]**	**10,289**	**13.97**	**10,643**	**14.45**	**10,563**	**14.34**	**100.0%**

* All costs by category are averaged over the total number of patients in each category (i.e., patients or caregivers with zero costs are not excluded in the average)
[1] Only two patients (one inpatient, one outpatient) had any DMCs, but costs are averaged over the total number of patients
[2] Productivity losses are averaged over the total number of patients in each category (not averaged over the total number of caregivers); 6 outpatients and 2 inpatients had caregivers who reported wage losses, but wages are averaged over the total number of outpatients (n=31) and inpatients (n=9)
[3] Costs are first summed across categories for each patient and averaged across total patients.

For peer review only - http://bmjopen.bmj.com/site/about/guidelines.xhtml

Table 3. Scenario analyses of mean indirect costs for cholera cases with reported wage losses and assuming minimum wage in Nsanje District, Malawi, 2016–2020

	**Scenario 1: Among those with self-reported wages only[1]** Patients: n=5 Caregivers: n=15		**% of total indirect costs**	**Scenario 2: Self-reported + minimum wage for patients aged 15–50 years** Patients: n=11 Caregivers: n=45		**% of total indirect costs**	**Scenario 3: Self-reported + minimum wage for patients aged >5 years** Patients: n=19 Caregivers: n=45		**% of total indirect costs**
	**2020 MWK[3] (min, max)**	**2020 USD (min, max)**		**MWK (min, max)**	**2020 USD (min, max)**		**MWK (min, max)**	**2020 USD (min, max)**	
**Indirect, productivity losses**									
Patients[1]	7,807 (5,008–12,519)	10.60 (6.80–17.00)	63%	6,506 (2,692–12,519)	8.83 (3.66–17.00)	69%	7,161 (2,019–16,154)	9.78 (2.74–21.93)	71%
Caregivers	8,558 (823–25,039)	6.36 (1.12–33.99)	38%	5,306 (823–25,039)	3.94 (1.12–33.99)	31%	5,306 (823–25,039)	3.94 (1.12–33.99)	29%
**Total indirect costs**	**16,365**	**16.96**		**11,812**	**12.77**		**12,467**	**13.72**	

[1] All 5 patients who worked for a wage were outpatient

[2] MWK= Malawian kwacha

For peer review only - http://bmjopen.bmj.com/site/about/guidelines.xhtml

Table 4. Financial and food insecurity of cholera cases in Nsanje District, Malawi, 2016–2020

	Outpatient (n=31) n (%)		Inpatient (n=9) n (%)		Total (n=40) n (%)	
**Borrowing money to pay for health care services**						
Did not borrow money	17	(55)	3	(33)	20	(50)
Borrowed money from a family member	6	(19)	0	(0)	6	(15)
Borrowed money from a friend	4	(13)	5	(56)	9	(23)
Borrowed money from a cooperative	0	(0)	1	(11)	1	(3)
Borrowed money from a neighbor	1	(3)	0	(0)	1	(3)
Borrowed money from the village chief	1	(3)	0	(0)	1	(3)
Borrowed money from boss/employer	1	(3)	0	(0)	1	(3)
Borrowed money from church	1	(3)	0	(0)	1	(3)
**Sold items to pay for health care services**						
Did not sell anything	29	(94)	4	(44)	33	(83)
Rented out rice and maize plots	0	(0)	4	(44)	4	(10)
Sold radio	1	(3)	0	(0)	1	(3)
Sold livestock	1	(3)	1	(11)	2	(5)
**Frequency of household going without food in the past year**						
Never	4	(13)	1	(11)	5	(13)
Once every 3 months	7	(23)	1	(11)	8	(20)
Once per month	3	(10)	2	(22)	5	(13)
Twice per month	2	(6)	1	(11)	3	(8)
Once per week	8	(26)	1	(11)	9	(23)
Almost every day	3	(10)	0	(0)	3	(8)
Unknown/Missing	4	(13)	3	(33)	7	(18)

For peer review only - http://bmjopen.bmj.com/site/about/guidelines.xhtml

Cholera Surveillance In Malawi (CSIMA) Program
NSANJE DISTRICT USE ONLY

Form S.I.#:__________________ **Study label**

(To be filled by Data Management) N☐☐-☐☐☐☐

D-1: Cost-of-Illness Survey
First Interview (Day 0,1,2,3, 4, 5, 6, or 7)
Case Group: ☐ Clinical Case ☐ RDT Case ☐ Laboratory Case

Place of interview: ☐Health facility / ☐Home / ☐Other, specify: ___________________________
If at health facility, indicate point of care: ☐Out-Patient / ☐In-Patient
If In-Patient, name of admission ward: ___________________________
Date of Admission (dd/mm/yyyy): _____/_____/__________
Date of Discharge (dd/mm/yyyy): _____/_____/__________ [Ç: Look this up in the study facility record]

For SURVEY FIELD WORKER and PATIENT IDENTITY VERIFICATION
Please fill out this section with relevant information from the Facility Recruitment Form (# refers to question number on Recruitment Form).

Patient's Full Name (#2 & #3): ____

Patient's Clan (*mfunda*) Name (#5): ____

Contact Phone Number (#8): ____

Health facility where patient was recruited (See Study ID 2-digit):____________________________

Patient's Sex (#10): ☐ Male ☐ Female

Patient's Age (#11, #11.1): ☐☐ Years **OR** ☐☐ Months (if child is ≤24 months)

Scheduled Date of Interview (DD/MM/YYYY) (#35): ☐☐/☐☐/☐☐☐☐

For INTERVIEWER

Your Name: ____

Have you (interviewer) called to confirm the interview 1 day in advance? ☐ Yes ☐ No

Did you take an empty bucket with you? ☐ Yes ☐ No ☐ None available

Instructions for Interviewer:

*Please check that you have completed the **SES-WASH Survey** before starting this **D-1 Cost-of-Illness Survey, First Interview**.*

Use this instrument to interview an adult (head of household, parent, or guardian) that is familiar with the patient and his/her treatment over the last few days. If the patient is ≥18 years old, attempt to interview the patient unless s/he requires a caretaker (e.g., patient is elderly and/or has mental illness).

*Any places that say "**SCRIPT**" should be read out loud by you, the interviewer, to the patient or person answering on behalf of the patient. Any places that say "**Interviewer**" are notes for you to be aware of before continuing to the next survey question.*

For peer review only - http://bmjopen.bmj.com/site/about/guidelines.xhtml

Cholera Surveillance In Malawi (CSIMA) Program
NSANJE DISTRICT USE ONLY

Form S.I.#:__________________ **Study label**

(To be filled by Data Management) N☐☐-☐☐☐☐

Verifying Patient Identity

SCRIPT: My records show that you (or the patient) went or were taken to the [**health facility**] about 0 to 7 days ago. Can you please tell me your name in the village or your father's name to verify your identify?

Interviewer: *Please also verify visually the patient's sex as indicated above. The age should be within 1 year.*

1	Is this correct?	☐ Yes → **continue to 2**	☐ No	☐ Don't know

Interviewer: *The patient (or parent/guardian) should have responded "**Yes**" to question 1. If s/he did not, ask if the patient is another household member or if there has been a mistake. If the problem cannot be resolved and the individual who was treated at the health facility cannot be identified,* ***STOP the interview****.*

2	Could you please tell me your relationship to the patient?	☐ 1 = Self (patient)	☐ 2 = Mother	☐ 3 = Father
		☐ 4 = Sister	☐ 5 = Brother	☐ 6 = Grandmother
		☐ 7 = Grandfather		
		☐ 8 = Other (specify): ______________________		

SCRIPT: As you may recall, when you visited the [**health facility**] 0–7 days ago, you were read an informed consent form that described participation in this survey and study. We would like to remind you that there are no direct risks from participation in this study. Participation is free of charge, and will not affect your treatment in any way now nor in the future at any health facility.

The purpose of this current survey is to understand how your (or the patient's) cholera episode has affected him/her financially. The results of this study will help local health staff in improving our knowledge of how cholera affects people in Malawi so that we can be prepared for future cholera outbreaks.

As a thank you for participating in this Cost-of-Illness Survey, we will give you a bucket that you may use in various ways, such as drawing water or for washing your hands at the end of the survey. If you have a **health passport** available, it may be helpful to use it to help you answer these questions.

SECTION A: Patient History Before Day 0

SCRIPT: After the doctor examined you clinically / after taking a stool sample recently during your (or the patient's) visit to [**health facility**], the doctor suspected / we found the cholera bacteria present. I am going to ask you about how this recent cholera episode affected you (or the patient) and anyone that may have gone with you (or the patient) while seeking treatment.

First, I would like to understand how much you (or the patient) had to spend on any healthcare services (including traditional healers) **before** visiting the health facility on [**Day 0**].

3	How many ***days*** were you (or the patient) sick with diarrhea before going to [**health facility**] 3 to 7 days ago where you were diagnosed?	☐☐ Days<br>☐ 99 = Don't know

For peer review only - http://bmjopen.bmj.com/site/about/guidelines.xhtml

Cholera Surveillance In Malawi (CSIMA) Program
NSANJE DISTRICT USE ONLY

Form S.I.#:__________________ **Study label**

(To be filled by Data Management) **N☐☐-☐☐☐☐**

4	Before going to [**health facility**] for care 3 to 7 days ago, did you (or the patient) receive treatment for illness somewhere else?	☐ 1 = Yes<br>☐ 2 = No → **skip to 34 (SECTION C)**<br>☐ 9 = Don't know → **skip to 34 (SECTION C)**
5	How many treatment locations did you visit?<br><br>*Examples of treatment locations: traditional healer, CHAM facility, public facility, and private hospital. Each of these should be counted as separate treatment locations.*	☐☐ Treatment location(s)

SECTION B: Health Facility Visits Before Day 0

Instructions: *Please fill out the entire column (top to bottom) for each time the patient sought care at a treatment location before Day 0. Please provide the following details in* ***local Malawian kwacha (MWK) currency****. If additional days are needed, please use the additional pages provided.*

Questions #6 through #33 *must be filled out for each visit. The first column should be the patient's earliest visit. The last column filled out should be the visit closest to the health facility visit in which the patient was enrolled. Do not include the Day 0 health facility visit.*

Example: *If there was a visit to a traditional healer and a health facility on a single day, then this is 2 visits (2 columns) that must be recorded.*

Item		Visits Before Day 0			
		**Visit 1 (Earliest Visit)**	**Visit 2**	**Visit 3**	**Visit 4**
6	Where did you seek treatment?<br><br>**Code:**<br>1 = District hospital<br>2 = Health centre/post<br>3 = CHAM/private<br>4 = Traditional healer/herbalist<br>5 = Other (specify)<br>9 = Don't know	☐<br>If "**5**" entered, specify: ______	☐<br>If "**5**" entered, specify: ______	☐<br>If "**5**" entered, specify: ______	☐<br>If "**5**" entered, specify: ______
7	During this visit, did you (or the patient) have to buy food and/or water for yourself?	☐ 1 = Yes<br>☐ 2 = No → **skip to 9**<br>☐ 9 = Don't know → **skip to 9**	☐ 1 = Yes<br>☐ 2 = No → **skip to 9**<br>☐ 9 = Don't know → **skip to 9**	☐ 1 = Yes<br>☐ 2 = No → **skip to 9**<br>☐ 9 = Don't know → **skip to 9**	☐ 1 = Yes<br>☐ 2 = No → **skip to 9**<br>☐ 9 = Don't know → **skip to 9**
8	How much did you pay for the food and/or water?<br><br>*Write "0" if nothing was paid.*	**MWK**<br>☐ 9 = Don't know	**MWK**<br>☐ 9 = Don't know	**MWK**<br>☐ 9 = Don't know	**MWK**<br>☐ 9 = Don't know

For peer review only - http://bmjopen.bmj.com/site/about/guidelines.xhtml

Cholera Surveillance In Malawi (CSIMA) Program
NSANJE DISTRICT USE ONLY

Form S.I.#:__________________ **Study label**

(To be filled by Data Management) **N**☐☐-☐☐☐☐

9	Not including food and/or water, how much did you pay for this visit? *Write "0" if nothing was paid.*	**MWK** ☐ 9 = Don't know	**MWK** ☐ 9 = Don't know	**MWK** ☐ 9 = Don't know	**MWK** ☐ 9 = Don't know
10	What was this payment for? *(Specify up to 3 choices)* **Code:** 1 = Medicine/drugs 2 = Diagnostic/lab fee 3 = Doctor, nurse, or herbalist fee 4 = Other (specify) 6 = Does not apply 9 = Don't know	Response 1: ☐ Response 2: ☐ Response 3: ☐ If "**4**" entered, specify: ____	Response 1: ☐ Response 2: ☐ Response 3: ☐ If "**4**" entered, specify: ____	Response 1: ☐ Response 2: ☐ Response 3: ☐ If "**4**" entered, specify: ____	Response 1: ☐ Response 2: ☐ Response 3: ☐ If "**4**" entered, specify: ____
11	Did you pay for anything outside of this treatment location? *Example: the healer asked you to buy **roots** for medicinal use after your visit.*	☐ 1 = Yes (specify): ____ ☐ 2 = No → **skip to 13** ☐ 9 = Don't know → **skip to 13**	☐ 1 = Yes (specify): ____ ☐ 2 = No → **skip to 13** ☐ 9 = Don't know → **skip to 13**	☐ 1 = Yes (specify): ____ ☐ 2 = No → **skip to 13** ☐ 9 = Don't know → **skip to 13**	☐ 1 = Yes (specify): ____ ☐ 2 = No → **skip to 13** ☐ 9 = Don't know → **skip to 13**
12	How much did it cost?	**MWK** ☐ 9 = Don't know	**MWK** ☐ 9 = Don't know	**MWK** ☐ 9 = Don't know	**MWK** ☐ 9 = Don't know
13	Who paid for the visit? *(Specify up to 3 choices)*	Response 1: ☐ Response 2: ☐	Response 1: ☐ Response 2: ☐	Response 1: ☐ Response 2: ☐	Response 1: ☐ Response 2: ☐

For peer review only

For peer review only - http://bmjopen.bmj.com/site/about/guidelines.xhtml

Cholera Surveillance In Malawi (CSIMA) Program
NSANJE DISTRICT USE ONLY

Form S.I.#:__________________ **Study label**

(To be filled by Data Management) N☐☐-☐☐☐☐

	**Code:**<br>1 = Self<br>2 = Patient's family<br>3 = Other (specify), including insurance<br>6 = Did not pay anything<br>9 = Don't know	Response 3: ☐<br>If "**3**" entered, specify: ________	Response 3: ☐<br>If "**3**" entered, specify: ________	Response 3: ☐<br>If "**3**" entered, specify: ________	Response 3: ☐<br>If "**3**" entered, specify: ________
**SCRIPT**: Now, I would like to understand how you **got to the treatment location and then back home**. I will also ask you questions about **whether anyone came with you** to the treatment location.					
14	How did you get to the treatment facility?<br>*(Specify up to 3 choices)*<br>**Code:**<br>1 = On foot/walking<br>2 = Bicycle<br>3 = Motorcycle<br>4 = Minibus<br>5 = Private car<br>6 = Horse cart<br>7 = Boat<br>8 = Other (specify)<br>9 = Don't know	**Travelled by:**<br>Response 1: ☐<br>Response 2: ☐<br>Response 3: ☐<br>If "**8**" entered, specify: ________	**Travelled by:**<br>Response 1: ☐<br>Response 2: ☐<br>Response 3: ☐<br>If "**8**" entered, specify: ________	**Travelled by:**<br>Response 1: ☐<br>Response 2: ☐<br>Response 3: ☐<br>If "**8**" entered, specify: ________	**Travelled by:**<br>Response 1: ☐<br>Response 2: ☐<br>Response 3: ☐<br>If "**8**" entered, specify: ________
15	How long did it take for the patient to get there?	**Travel time:**<br>☐☐☐ **Minutes**	**Travel time:**<br>☐☐☐ **Minutes**	**Travel time:**<br>☐☐☐ **Minutes**	**Travel time:**<br>☐☐☐ **Minutes**
16	How much did it cost for the patient to get there?<br>*Write "0" if nothing was paid.*	**MWK**<br>☐ 999 = Don't know	**MWK**<br>☐ 999 = Don't know	**MWK**<br>☐ 999 = Don't know	**MWK**<br>☐ 999 = Don't know
17	Did you travel to the location alone?	☐ 1 = Yes<br>→ **skip to 20**<br>☐ 2 = No	☐ 1 = Yes<br>→ **skip to 20**<br>☐ 2 = No	☐ 1 = Yes<br>→ **skip to 20**<br>☐ 2 = No	☐ 1 = Yes<br>→ **skip to 20**<br>☐ 2 = No
18	How many people came with you (excluding the patient)?	☐☐ People<br>☐ 99 = Don't know	☐☐ People<br>☐ 99 = Don't know	☐☐ People<br>☐ 99 = Don't know	☐☐ People<br>☐ 99 = Don't know

For peer review only - http://bmjopen.bmj.com/site/about/guidelines.xhtml

Cholera Surveillance In Malawi (CSIMA) Program
NSANJE DISTRICT USE ONLY

Form S.I.#:__________________ **Study label**

(To be filled by Data Management) N☐☐-☐☐☐☐

	*If patient answered "No" to 17, this **cannot** be "00"*				
19	How much did it cost for <u>each person</u> that came with you?<br><br>*Write "0" if nothing was paid.*	**MWK Per person**<br><br>☐ 999 = Don't know	**MWK Per person**<br><br>☐ 999 = Don't know	**MWK Per person**<br><br>☐ 999 = Don't know	**MWK Per person**<br><br>☐ 999 = Don't know
20	Did anybody follow you to the treatment location (did they come separately)?	☐ 1 = Yes<br>☐ 2 = No<br>**→ skip to 25**	☐ 1 = Yes<br>☐ 2 = No<br>**→ skip to 25**	☐ 1 = Yes<br>☐ 2 = No<br>**→ skip to 25**	☐ 1 = Yes<br>☐ 2 = No<br>**→ skip to 25**
21	How many people met you at the location?<br><br>*If patient answered "Yes" to 20, this **cannot** be "00"*	☐☐ People<br><br>☐ 99 = Don't know	☐☐ People<br><br>☐ 99 = Don't know	☐☐ People<br><br>☐ 99 = Don't know	☐☐ People<br><br>☐ 99 = Don't know
22	How did the person(s) get to the location?<br>*(Specify up to 3 choices)*<br><br>**Code:**<br>1 = On foot/walking<br>2 = Bicycle<br>3 = Motorcycle<br>4 = Minibus<br>5 = Private car<br>6 = Horse cart<br>7 = Boat<br>8 = Other (specify)<br>9 = Don't know	**Travelled by:**<br>Response 1: ☐<br>Response 2: ☐<br>Response 3: ☐<br>If "**8**" entered, specify:<br>______________	**Travelled by:**<br>Response 1: ☐<br>Response 2: ☐<br>Response 3: ☐<br>If "**8**" entered, specify:<br>______________	**Travelled by:**<br>Response 1: ☐<br>Response 2: ☐<br>Response 3: ☐<br>If "**8**" entered, specify:<br>______________	**Travelled by:**<br>Response 1: ☐<br>Response 2: ☐<br>Response 3: ☐<br>If "**8**" entered, specify:<br>______________
23	How long did it take the person(s)?<br><br>*Enter time <u>per person</u>.*	**Travel time:**<br>☐☐☐<br>**Minutes per person**<br>☐ 999 = Don't know	**Travel time:**<br>☐☐☐<br>**Minutes per person**<br>☐ 999 = Don't know	**Travel time:**<br>☐☐☐<br>**Minutes per person**<br>☐ 999 = Don't know	**Travel time:**<br>☐☐☐<br>**Minutes per person**<br>☐ 999 = Don't know

For peer review only - http://bmjopen.bmj.com/site/about/guidelines.xhtml

Cholera Surveillance In Malawi (CSIMA) Program
NSANJE DISTRICT USE ONLY

Form S.I.#:_________________ **Study label**

(To be filled by Data Management) N☐☐-☐☐☐☐

24	How much did it cost the person(s)?<br><br>*Enter cost per person. Write "0" if nothing was paid.*	**MWK**<br>**Per person**<br>☐ 999 = Don't know	**MWK**<br>**Per person**<br>☐ 999 = Don't know	**MWK**<br>**Per person**<br>☐ 999 = Don't know	**MWK**<br>**Per person**<br>☐ 999 = Don't know
25	While at the facility, how much did everyone who came with you or met you at the treatment facility spend on food and/or water (excluding patient)?<br><br>*Enter cost for all persons combined. Write "0" if no food and/or water were purchased.*	**MWK**<br>**Total for all persons**<br>☐ Respondent unsure, but estimated<br>☐ 666 = Went alone<br>☐ 999 = Don't know	**MWK**<br>**Total for all persons**<br>☐ Respondent unsure, but estimated<br>☐ 666 = Went alone<br>☐ 999 = Don't know	**MWK**<br>**Total for all persons**<br>☐ Respondent unsure, but estimated<br>☐ 666 = Went alone<br>☐ 999 = Don't know	**MWK**<br>**Total for all persons**<br>☐ Respondent unsure, but estimated<br>☐ 666 = Went alone<br>☐ 999 = Don't know
26	How much did the person(s) spend on lodging (total) if an overnight stay was needed?<br><br>*Enter cost for all persons combined. Write "0" if no overnight stay was needed.*	**MWK**<br>**Total for all persons**<br>☐ 666 = Was alone<br>☐ 999 = Don't know	**MWK**<br>**Total for all persons**<br>☐ 666 = Was alone<br>☐ 999 = Don't know	**MWK**<br>**Total for all persons**<br>☐ 666 = Was alone<br>☐ 999 = Don't know	**MWK**<br>**Total for all persons**<br>☐ 666 = Was alone<br>☐ 999 = Don't know
**SCRIPT:** Now, I would like to understand how you (or the patient) **travelled back home after the visit** to the treatment location.					
27	How did you get home?<br>*(Specify up to 3 choices)*	**Travelled by:**<br>Response 1: ☐	**Travelled by:**<br>Response 1: ☐	**Travelled by:**<br>Response 1: ☐	**Travelled by:**<br>Response 1: ☐

For peer review only - http://bmjopen.bmj.com/site/about/guidelines.xhtml

Cholera Surveillance In Malawi (CSIMA) Program
NSANJE DISTRICT USE ONLY

Form S.I.#:________________ **Study label**

(To be filled by Data Management) **N**☐☐-☐☐☐☐

	**Code:**<br>1 = On foot/walking<br>2 = Bicycle<br>3 = Motorcycle<br>4 = Minibus<br>5 = Private car<br>6 = Horse cart<br>7 = Boat<br>8 = Other (specify)<br>9 = Don't know	Response 2: ☐<br>Response 3: ☐<br>If "**8**" entered, specify:<br>________	Response 2: ☐<br>Response 3: ☐<br>If "**8**" entered, specify:<br>________	Response 2: ☐<br>Response 3: ☐<br>If "**8**" entered, specify:<br>________	Response 2: ☐<br>Response 3: ☐<br>If "**8**" entered, specify:<br>________
28	How long did it take for the patient?	**Travel time:**<br>☐☐☐ **Minutes**<br>☐ 999 = Don't know	**Travel time:**<br>☐☐☐ **Minutes**<br>☐ 999 = Don't know	**Travel time:**<br>☐☐☐ **Minutes**<br>☐ 999 = Don't know	**Travel time:**<br>☐☐☐ **Minutes**<br>☐ 999 = Don't know
29	How much did it cost for the patient?<br>*Write "0" if nothing was paid.*	**MWK**<br>☐ 999 = Don't know	**MWK**<br>☐ 999 = Don't know	**MWK**<br>☐ 999 = Don't know	**MWK**<br>☐ 999 = Don't know
30	Did you travel back to your home <u>alone</u>?	☐ 1 = Yes<br>→ **skip to 33**<br>☐ 2 = No	☐ 1 = Yes<br>→ **skip to 33**<br>☐ 2 = No	☐ 1 = Yes<br>→ **skip to 33**<br>☐ 2 = No	☐ 1 = Yes<br>→ **skip to 33**<br>☐ 2 = No
31	How many people travelled home with you (<u>excluding</u> you)?<br>*If patient answered "No" to 30, this **cannot** be "00"*	☐☐ People<br>☐ 99 = Don't know	☐☐ People<br>☐ 99 = Don't know	☐☐ People<br>☐ 99 = Don't know	☐☐ People<br>☐ 99 = Don't know
32	How much did it cost for <u>each person</u> that came with you?<br>*Write "0" if nothing was paid.*	**MWK Per person**<br>☐ 999 = Don't know	**MWK Per person**<br>☐ 999 = Don't know	**MWK Per person**<br>☐ 999 = Don't know	**MWK Per person**<br>☐ 999 = Don't know
	Interviewer: *Please add any additional notes regarding travelling to and from the treatment center in **Question #33**.*<br><br>***For example:*** *"While patient was at CHAM facility with his/her mother, the patient required food. The mother traveled home by foot to bring the home-cooked food to the CHAM facility for the patient to eat."*				
33	Additional notes regarding patient or companion travel.				

CHECK for Interviewer

For peer review only

For peer review only - http://bmjopen.bmj.com/site/about/guidelines.xhtml

Cholera Surveillance In Malawi (CSIMA) Program
NSANJE DISTRICT USE ONLY

Form S.I.#:__________________ **Study label**

(To be filled by Data Management) N☐☐-☐☐☐☐

How many treatment locations did the patient specify in Question #5?
☐☐ Treatment location(s)

Please check that the **number of treatment location(s) matches the number of columns** that you have filled out in **Section B** before proceeding to **Section C**. **Questions #6 through #33** must be filled out for each visit. If there was a visit to a traditional healer and a health facility on a single day, then this is 2 visits (2 columns) that must be recorded.

Have you verified?
☐ Yes → **continue to Section C**

SECTION C: Health Facility Visits Starting at Day 0

SCRIPT: Now, I would like to understand how much you (or the patient) had to spend on any healthcare services (including traditional healers) **starting on the day you first visited the health facility** where we diagnosed and treated you on Day 0.

Instructions: *Please fill out the entire column (top to bottom) for each time the patient sought care at a treatment location starting at* ***Day 0****. Please provide the following details in* ***local Malawian kwacha (MWK) currency****. If additional days are needed, please use the additional pages provided.*

Questions #34 through #61 *must be filled out for each visit. The first column should be the patient's first visit to the health facility in which the patient was enrolled (i.e., Day 0). The last column filled out should be the visit closest to today's interview. Do* ***not*** *include any visits to any type of treatment location today.*

Item		Visits Starting at Day 0			
		**Day 0**	**Visit 1**	**Visit 2**	**Visit 3**
34	Where did you seek treatment?<br><br>**Code:**<br>1 = District hospital<br>2 = Health centre/post<br>3 = CHAM/private<br>4 = Traditional healer/herbalist<br>5 = Other (specify)<br>9 = Don't know	☐<br><br>If "**5**" entered, specify:<br>______	☐<br><br>If "**5**" entered, specify:<br>______	☐<br><br>If "**5**" entered, specify:<br>______	☐<br><br>If "**5**" entered, specify:<br>______
35	During this visit, did you (or the patient) have to buy food and/or water for yourself?	☐ 1 = Yes<br>☐ 2 = No<br>→ **skip to 37**<br>☐ 9 = Don't know<br>→ **skip to 37**	☐ 1 = Yes<br>☐ 2 = No<br>→ **skip to 37**<br>☐ 9 = Don't know<br>→ **skip to 37**	☐ 1 = Yes<br>☐ 2 = No<br>→ **skip to 37**<br>☐ 9 = Don't know<br>→ **skip to 37**	☐ 1 = Yes<br>☐ 2 = No<br>→ **skip to 37**<br>☐ 9 = Don't know<br>→ **skip to 37**
36	How much did you pay for the food and/or water?	**MWK**	**MWK**	**MWK**	**MWK**

For peer review only - http://bmjopen.bmj.com/site/about/guidelines.xhtml

Cholera Surveillance In Malawi (CSIMA) Program
NSANJE DISTRICT USE ONLY

Form S.I.#:__________________ **Study label**

(To be filled by Data Management) N☐☐-☐☐☐☐

		☐ 9 = Don't know	☐ 9 = Don't know	☐ 9 = Don't know	☐ 9 = Don't know
37	Not including food and/or water, how much did you pay for this visit?<br>*Write "0" if nothing was paid.*	**MWK**<br>☐ 9 = Don't know	**MWK**<br>☐ 9 = Don't know	**MWK**<br>☐ 9 = Don't know	**MWK**<br>☐ 9 = Don't know
38	What was this payment for? *(Specify up to 3 choices)*<br>**Code:**<br>1 = Medicine/drugs<br>2 = Diagnostic/lab fee<br>3 = Doctor, nurse, or herbalist fee<br>4 = Other (specify)<br>6 = Does not apply<br>9 = Don't know	Response 1: ☐<br>Response 2: ☐<br>Response 3: ☐<br>If "**4**" entered, specify: ________	Response 1: ☐<br>Response 2: ☐<br>Response 3: ☐<br>If "**4**" entered, specify: ________	Response 1: ☐<br>Response 2: ☐<br>Response 3: ☐<br>If "**4**" entered, specify: ________	Response 1: ☐<br>Response 2: ☐<br>Response 3: ☐<br>If "**4**" entered, specify: ________
39	Did you pay for anything outside of this treatment location?<br>*Example: the healer asked you to buy **roots** for medicinal use after your visit.*	☐ 1 = Yes (specify): ________<br>☐ 2 = No → **skip to 41**<br>☐ 9 = Don't know → **skip to 41**	☐ 1 = Yes (specify): ________<br>☐ 2 = No → **skip to 41**<br>☐ 9 = Don't know → **skip to 41**	☐ 1 = Yes (specify): ________<br>☐ 2 = No → **skip to 41**<br>☐ 9 = Don't know → **skip to 41**	☐ 1 = Yes (specify): ________<br>☐ 2 = No → **skip to 41**<br>☐ 9 = Don't know → **skip to 41**
40	How much did it cost?	**MWK**<br>☐ 9 = Don't know	**MWK**<br>☐ 9 = Don't know	**MWK**<br>☐ 9 = Don't know	**MWK**<br>☐ 9 = Don't know
41	Who paid for the visit?	Response 1: ☐	Response 1: ☐	Response 1: ☐	Response 1: ☐

IVI_CSIMA_ v4.1, 24-April-2017

For peer review only - http://bmjopen.bmj.com/site/about/guidelines.xhtml

Cholera Surveillance In Malawi (CSIMA) Program

NSANJE DISTRICT USE ONLY

Form S.I.#:__________________

(To be filled by Data Management)

Study label

N☐☐-☐☐☐☐

	(Specify up to 3 choices)<br><br>**Code:**<br>1 = Self<br>2 = Patient's family<br>3 = Other (specify), including insurance<br>6 = Did not pay anything<br>9 = Don't know	Response 2: ☐<br>Response 3: ☐<br><br>If "**3**" entered, specify:<br>__________	Response 2: ☐<br>Response 3: ☐<br><br>If "**3**" entered, specify:<br>__________	Response 2: ☐<br>Response 3: ☐<br><br>If "**3**" entered, specify:<br>__________	Response 2: ☐<br>Response 3: ☐<br><br>If "**3**" entered, specify:<br>__________
**SCRIPT:** Now, I would like to understand how you **got to the treatment location and then back home**. I will also ask you questions about **whether anyone came with you** to the treatment location.					
42	How did you get to the treatment facility?<br>*(Specify up to 3 choices)*<br><br>**Code:**<br>1 = On foot/walking<br>2 = Bicycle<br>3 = Motorcycle<br>4 = Minibus<br>5 = Private car<br>6 = Horse cart<br>7 = Boat<br>8 = Other (specify)<br>9 = Don't know	**Travelled by:**<br>Response 1: ☐<br>Response 2: ☐<br>Response 3: ☐<br><br>If "**8**" entered, specify:<br>__________	**Travelled by:**<br>Response 1: ☐<br>Response 2: ☐<br>Response 3: ☐<br><br>If "**8**" entered, specify:<br>__________	**Travelled by:**<br>Response 1: ☐<br>Response 2: ☐<br>Response 3: ☐<br><br>If "**8**" entered, specify:<br>__________	**Travelled by:**<br>Response 1: ☐<br>Response 2: ☐<br>Response 3: ☐<br><br>If "**8**" entered, specify:<br>__________
43	How long did it take for the patient to get there?	**Travel time:**<br>☐☐☐ **Minutes**	**Travel time:**<br>☐☐☐ **Minutes**	**Travel time:**<br>☐☐☐ **Minutes**	**Travel time:**<br>☐☐☐ **Minutes**
44	How much did it cost for the patient to get there?<br><br>*Write "0" if nothing was paid.*	**MWK**<br><br>☐ 999 = Don't know	**MWK**<br><br>☐ 999 = Don't know	**MWK**<br><br>☐ 999 = Don't know	**MWK**<br><br>☐ 999 = Don't know
45	Did you travel to the location <u>alone</u>?	☐ 1 = Yes<br>→ **skip to 48**<br><br>☐ 2 = No	☐ 1 = Yes<br>→ **skip to 48**<br><br>☐ 2 = No	☐ 1 = Yes<br>→ **skip to 48**<br><br>☐ 2 = No	☐ 1 = Yes<br>→ **skip to 48**<br><br>☐ 2 = No
46	How many people came with you	☐☐ People	☐☐ People	☐☐ People	☐☐ People

For peer review only

For peer review only - http://bmjopen.bmj.com/site/about/guidelines.xhtml

Cholera Surveillance In Malawi (CSIMA) Program

NSANJE DISTRICT USE ONLY

Form S.I.#:__________________ **Study label**

(To be filled by Data Management) N☐☐-☐☐☐☐

	(excluding the patient)?<br><br>*If patient answered "No" to 45, this **cannot** be "00"*	☐ 99 = Don't know	☐ 99 = Don't know	☐ 99 = Don't know	☐ 99 = Don't know
47	How much did it cost for each person that came with you?<br><br>*Write "0" if nothing was paid.*	**MWK Per person**<br><br>☐ 999 = Don't know	**MWK Per person**<br><br>☐ 999 = Don't know	**MWK Per person**<br><br>☐ 999 = Don't know	**MWK Per person**<br><br>☐ 999 = Don't know
48	Did anybody follow you to the treatment location (did they come separately)?	☐ 1 = Yes<br>☐ 2 = No<br>→ **skip to 53**	☐ 1 = Yes<br>☐ 2 = No<br>→ **skip to 53**	☐ 1 = Yes<br>☐ 2 = No<br>→ **skip to 53**	☐ 1 = Yes<br>☐ 2 = No<br>→ **skip to 53**
49	How many people met you at the location?<br><br>*If patient answered "Yes" to 48, this **cannot** be "00"*	☐☐ People<br><br>☐ 99 = Don't know	☐☐ People<br><br>☐ 99 = Don't know	☐☐ People<br><br>☐ 99 = Don't know	☐☐ People<br><br>☐ 99 = Don't know
50	How did the person(s) get to the location?<br>*(Specify up to 3 choices)*<br><br>**Code:**<br>1 = On foot/walking<br>2 = Bicycle<br>3 = Motorcycle<br>4 = Minibus<br>5 = Private car<br>6 = Horse cart<br>7 = Boat<br>8 = Other (specify)<br>9 = Don't know	**Travelled by:**<br>Response 1: ☐<br>Response 2: ☐<br>Response 3: ☐<br><br>If "**8**" entered, specify:<br>______________	**Travelled by:**<br>Response 1: ☐<br>Response 2: ☐<br>Response 3: ☐<br><br>If "**8**" entered, specify:<br>______________	**Travelled by:**<br>Response 1: ☐<br>Response 2: ☐<br>Response 3: ☐<br><br>If "**8**" entered, specify:<br>______________	**Travelled by:**<br>Response 1: ☐<br>Response 2: ☐<br>Response 3: ☐<br><br>If "**8**" entered, specify:<br>______________
51	How long did it take the person(s)?	**Travel time:**<br>☐☐☐<br>**Minutes per person**	**Travel time:**<br>☐☐☐<br>**Minutes per person**	**Travel time:**<br>☐☐☐<br>**Minutes per person**	**Travel time:**<br>☐☐☐<br>**Minutes per person**

For peer review only - http://bmjopen.bmj.com/site/about/guidelines.xhtml

Cholera Surveillance In Malawi (CSIMA) Program

NSANJE DISTRICT USE ONLY

Form S.I.#:__________________ **Study label**

(To be filled by Data Management) N☐☐-☐☐☐☐

	Enter time per person.	☐ 999 = Don't know	☐ 999 = Don't know	☐ 999 = Don't know	☐ 999 = Don't know
52	How much did it cost the person(s)?<br><br>*Enter cost per person. Write "0" if nothing was paid.*	**MWK Per person**<br>☐ 999 = Don't know	**MWK Per person**<br>☐ 999 = Don't know	**MWK Per person**<br>☐ 999 = Don't know	**MWK Per person**<br>☐ 999 = Don't know
53	While at the facility, how much did everyone who came with you or met you at the treatment facility spend on food and/or water (excluding patient)?<br><br>*Enter cost for all persons combined. Write "0" if no food and/or water were purchased.*	**MWK Total for all persons**<br>☐ Respondent unsure, but estimated<br>☐ 666 = Went alone<br>☐ 999 = Don't know	**MWK Total for all persons**<br>☐ Respondent unsure, but estimated<br>☐ 666 = Went alone<br>☐ 999 = Don't know	**MWK Total for all persons**<br>☐ Respondent unsure, but estimated<br>☐ 666 = Went alone<br>☐ 999 = Don't know	**MWK Total for all persons**<br>☐ Respondent unsure, but estimated<br>☐ 666 = Went alone<br>☐ 999 = Don't know
54	How much did the person(s) spend on lodging (total) if an overnight stay was needed?<br><br>*Enter cost for all persons combined. Write "0" if no overnight stay was needed.*	**MWK Total for all persons**<br>☐ 666 = Was alone<br>☐ 999 = Don't know	**MWK Total for all persons**<br>☐ 666 = Was alone<br>☐ 999 = Don't know	**MWK Total for all persons**<br>☐ 666 = Was alone<br>☐ 999 = Don't know	**MWK Total for all persons**<br>☐ 666 = Was alone<br>☐ 999 = Don't know
**SCRIPT:** Now, I would like to understand how you (or the patient) **travelled back home after the visit** to the treatment location.					
55	How did you get home?	**Travelled by:**	**Travelled by:**	**Travelled by:**	**Travelled by:**

For peer review only - http://bmjopen.bmj.com/site/about/guidelines.xhtml

For peer review only

Cholera Surveillance In Malawi (CSIMA) Program
NSANJE DISTRICT USE ONLY

Form S.I.#:__________________ **Study label**

(To be filled by Data Management) N☐☐-☐☐☐☐

	(Specify up to 3 choices)<br><br>**Code:**<br>1 = On foot/walking<br>2 = Bicycle<br>3 = Motorcycle<br>4 = Minibus<br>5 = Private car<br>6 = Horse cart<br>7 = Boat<br>8 = Other (specify)<br>9 = Don't know	Response 1: ☐<br>Response 2: ☐<br>Response 3: ☐<br>If "**8**" entered, specify:<br>__________	Response 1: ☐<br>Response 2: ☐<br>Response 3: ☐<br>If "**8**" entered, specify:<br>__________	Response 1: ☐<br>Response 2: ☐<br>Response 3: ☐<br>If "**8**" entered, specify:<br>__________	Response 1: ☐<br>Response 2: ☐<br>Response 3: ☐<br>If "**8**" entered, specify:<br>__________
56	How long did it take for the patient?	**Travel time:**<br>☐☐☐ **Minutes**<br>☐ 999 = Don't know	**Travel time:**<br>☐☐☐ **Minutes**<br>☐ 999 = Don't know	**Travel time:**<br>☐☐☐ **Minutes**<br>☐ 999 = Don't know	**Travel time:**<br>☐☐☐ **Minutes**<br>☐ 999 = Don't know
57	How much did it cost for the patient?<br>*Write "0" if nothing was paid.*	**MWK**<br>☐ 999 = Don't know	**MWK**<br>☐ 999 = Don't know	**MWK**<br>☐ 999 = Don't know	**MWK**<br>☐ 999 = Don't know
58	Did you travel back to your home alone?	☐ 1 = Yes<br>→ **skip to 61**<br>☐ 2 = No	☐ 1 = Yes<br>→ **skip to 61**<br>☐ 2 = No	☐ 1 = Yes<br>→ **skip to 61**<br>☐ 2 = No	☐ 1 = Yes<br>→ **skip to 61**<br>☐ 2 = No
59	How many people travelled home with you (excluding you)?<br>*If patient answered "No" to 58, this **cannot** be "00"*	☐☐ People<br>☐ 99 = Don't know	☐☐ People<br>☐ 99 = Don't know	☐☐ People<br>☐ 99 = Don't know	☐☐ People<br>☐ 99 = Don't know
60	How much did it cost for each person that came with you?<br>*Write "0" if nothing was paid.*	**MWK**<br>**Per person**<br>☐ 999 = Don't know	**MWK**<br>**Per person**<br>☐ 999 = Don't know	**MWK**<br>**Per person**<br>☐ 999 = Don't know	**MWK**<br>**Per person**<br>☐ 999 = Don't know

***Interviewer:** Please add any additional notes regarding travelling to and from the treatment center in **Question #61**.*

***For example:** "While patient was at CHAM facility with his/her mother, the patient required food. The mother traveled home by foot (30 minutes roundtrip) to bring the home-cooked food to the CHAM facility for the patient to eat."*

61	Additional notes regarding patient or				

For peer review only - http://bmjopen.bmj.com/site/about/guidelines.xhtml

Cholera Surveillance In Malawi (CSIMA) Program
NSANJE DISTRICT USE ONLY

Form S.I.#:__________________ **Study label**

(To be filled by Data Management) N☐☐-☐☐☐☐

	companion travel.				

CHECK for Interviewer

SCRIPT: Did you go anywhere else to seek treatment for your cholera since your recent health facility visit 3 to 7 days ago? Remember, treatment locations also include the traditional healer and places other than the health facility where we provided you (or the patient) treatment.

Interviewer: *If the patient says "Yes," please fill out another column starting from* ***Question #34 through #61****.* *Repeat this process until the patient says "No."*

Have you verified that the patient has not gone anywhere else for care since his/her Day 0 visit?

☐ Yes → **continue to Section D**

SECTION D: Indirect Costs: Productivity Losses & Opportunity Costs

SCRIPT: Now, I would like to ask you about the ways you (or the patient's) cholera illness affected those around you. Sometimes, people with cholera feel so sick that they cannot perform any of their usual activities. Instead, they have to rest and stay in bed. Other people around the patient may not be as sick, and can still do their usual activities. But, they may not be able to perform their activities as well as normal because they may have to help or take care of the patient.

When you answer these questions, please think about the entire time period when you were sick with cholera, starting with before you (or the patient) went to the health facility for diagnosis.

62	In **total**, how many days have you (or the patient) been sick with cholera since the symptoms started?	☐☐ Days	☐ 99 = Don't know
63	How many days were you (or the patient) **completely unable** to do any of your usual activities while sick?	☐☐ Days	☐ 99 = Don't know
64	How many days were you (or the patient) able to perform **some but not all** of your usual activities while sick?	☐☐ Days	☐ 99 = Don't know
65	How many days were you (or the patient) able to perform **all** of your usual activities while sick?	☐☐ Days	☐ 99 = Don't know

ERROR CHECK: *Please check that the number of days of no activity, some activity, and normal activity add up to the total number of days ill. If the days do not add up correctly, please review* ***Questions #62 through #65*** *with the patient.*

Days of **no activity** while ill (**Question #63**)	+	Days of **some activity** while ill (**Question #64**)	+	Days of **normal activity** while ill (**Question #65**)	=	Total number of days ill (**Question #62**)
☐☐ Days	+	☐☐ Days	+	☐☐ Days	=	☐☐ Days

66	What would you (or the patient) be doing if you had not been sick?<br><br>*If the patient is an infant/toddler, select "5" for Leisure or play time. Check all that apply.*	☐ 1 = Going to school<br>☐ 2 = Working for a wage (e.g. for an employer, paid)<br>☐ 3 = Working for self (e.g. fishing or farming, unpaid)<br>☐ 4 = Attending church<br>☐ 5 = Leisure or play time<br>☐ 6 = Helping a parent<br>☐ 7 = Housework (e.g. cooking, cleaning, child care)<br>☐ 8 = Other (specify): ____________________

For peer review only - http://bmjopen.bmj.com/site/about/guidelines.xhtml

Cholera Surveillance In Malawi (CSIMA) Program
NSANJE DISTRICT USE ONLY

Form S.I.#:__________________ **Study label**

(To be filled by Data Management) N☐☐-☐☐☐☐

67	Do you (or the patient) work for a wage (cash or in-kind)?	☐ 1 = Yes, paid in cash (specify amount **per day**): ______________ **MWK**<br>☐ 2 = Yes, paid in-kind (specify what): ______________<br>☐ 3 = No
68	If you work for a wage, did your (or the patient's) employer or boss pay for the days you were <u>sick but did not come to work</u>? *For example, if the patient has sick leave.*	☐ 1 = Yes<br>☐ 2 = No → **skip to 70**<br>☐ 9 = Don't know → **skip to 70**
69	How many sick days did your (or the patient's) employer or boss pay you for?	☐☐ Days<br>☐ 66 = Patient does not work ☐ 99 = Don't know
70	In total, how many days of income did you (or the patient) lose because of this illness <u>since it started</u>?	☐☐ Days<br>☐ 66 = Patient does not work ☐ 99 = Don't know

SECTION E: Indirect Costs: Caretakers & Substitute Labor ("Helpers")

<u>**SCRIPT**</u>**:** Now, I would like to understand if anyone helped you (or the patient) while you were sick. This includes 2 types of people:

1. Someone who may have been paid to complete your usual tasks for you
2. Someone who had to help take care of you (or the patient) while you were sick

Sometimes, this is the same person.

71	Were you (or the patient) so sick with cholera that someone had to help you (or the patient) in any way?<br><br>*This includes helpers who had to skip their own usual work or activities to help you.*	☐ 1 = Yes<br>☐ 2 = No → **skip to 85 (SECTION F)**<br>☐ 9 = Don't know → **skip to 85 (SECTION F)**
72	How many people cared for you (or the patient) or did your usual activities for you while you were sick with cholera?	☐☐ People ☐ 99 = Don't know

For peer review only - http://bmjopen.bmj.com/site/about/guidelines.xhtml

Cholera Surveillance In Malawi (CSIMA) Program
NSANJE DISTRICT USE ONLY

Form S.I.#:___________________ **Study label**

(To be filled by Data Management) **N□□-□□□□**

(SECTION E CONTINUED)						
Interviewer: *Please enter responses for the number of people the patient indicated in* ***Question #72****. For example, if the patient responded "3", then lines A, B, and C should be filled out for each question.*						
**Helper number**	73. Patient's relationship with person who helped him/her while sick. *Write "hired help" if this is someone who was paid to perform the patient's usual activities, or was hired to provide care for the person.*	74. Is this helper a member of your household? **1** = Yes **2** = No **9** = Don't know	75. How old is this helper? **1** = Adult (17+) **2** = Teenager (12–16) **3** = Child (<12) **9** = Don't know	76. How many days or hours did s/he help you (or the patient)? *Record the number and* *circle days or hours.*	77. Was this helper paid to help you (or the patient)? **1** = Yes **2** = No **→ skip to 79** **9** = Don't know **→ skip to 79**	78. How much was this helper **paid per day** in Malawian kwacha (MWK) to help the patient?
**A**				**DAYS or HOURS**		**MWK**
**B**				**DAYS or HOURS**		**MWK**
**C**				**DAYS or HOURS**		**MWK**
**D**				**DAYS or HOURS**		**MWK**
**E**				**DAYS or HOURS**		**MWK**

For peer review only - http://bmjopen.bmj.com/site/about/guidelines.xhtml

Cholera Surveillance In Malawi (CSIMA) Program
NSANJE DISTRICT USE ONLY

Form S.I.#:__________________ **Study label**

(To be filled by Data Management) N☐☐-☐☐☐☐

Helper number	79. Did this helper perform:<br><br>**1** = <u>All</u> of the patient's activities<br>**2** = <u>Some</u> of the patient's activities<br>**3** = <u>None</u> of the patient's activities<br>**9** = Don't know	80. Did the helper cut back on his/her own usual activities?<br><br>**1** = Yes<br>**2** = No<br>→ **skip to 83**<br>**9** = Don't know<br>→ **skip to 83**	81. How many days did the helper cut back on his/her own duties?<br><br>*Record the number and <u>circle</u> days or hours.*	82. Was the helper able to do:<br><br>**1** = <u>Some</u> of his/her own activities<br>**2** = <u>None</u> of her/her own activities<br>**9** = Don't know	83. What would the helper have been doing if s/he had not been caring for the sick patient?<br><br>*Indicate all that apply.*<br><br>**1** = Going to school<br>**2** = Working for a wage (paid)<br>**3** = Working for self (unpaid)<br>**4** = Attending church<br>**5** = Leisure or play time<br>**6** = Helping parent<br>**7** = Housework<br>**8** = Other (specify)	84. If helper works for a wage, how much is he/she normally **paid per day** for his/her work in Malawian kwacha (MWK)?
**A**			**DAYS or HOURS**			**MWK**
**B**			**DAYS or HOURS**			**MWK**
**C**			**DAYS or HOURS**			**MWK**
**D**			**DAYS or HOURS**			**MWK**
**E**			**DAYS or HOURS**			**MWK**

CHECK for Interviewer

How many helpers did the patient specify in Question #72?

☐☐ People

Please check that the **number of people matches the number of rows** that you have filled out in **Section E** before proceeding to **Section F. Questions #73 through #84** must be filled out for <u>each</u> helper.

Have you verified?

☐ Yes → **continue to Section F**

SECTION F: Financial Impact of Cholera

<u>SCRIPT</u>: We are almost done with the interview. There are just a few more questions I would like to ask you to understand how this cholera event impacted your (or the patient's) financial situation.

85	Did you (or the patient) or your household have to borrow money from anyone in order to pay for the cholera treatment?	☐ 1 = Yes<br>☐ 2 = No → **skip to 89 (SECTION G)**	
86	Who did you (or the patient) or your household borrow money from?	☐ 1 = Family member<br>☐ 3 = Church	☐ 2 = Friend<br>☐ 4 = Neighbors

IVI_CSIMA_ v4.1, 24-April-2017

For peer review only - http://bmjopen.bmj.com/site/about/guidelines.xhtml

Cholera Surveillance In Malawi (CSIMA) Program
NSANJE DISTRICT USE ONLY

Form S.I.#:_________________ **Study label**

(To be filled by Data Management) **N☐☐-☐☐☐☐**

	Check all that apply.	☐ 5 = Village chief ☐ 6 = Boss/employer<br>☐ 7 = Social Grouping or Cooperative<br>☐ 8 = Other (specify): ____________________
87	Did your (or the patient's) household have to sell any items in order to raise money to pay for the cholera treatment?	☐ 1 = Yes<br>☐ 2 = No → **skip to 89 (SECTION G)**
88	What was sold?<br><br>*Check all that apply.*	☐ 1 = Clothing ☐ 2 = Livestock, specify:_______<br>☐ 3 = Radio ☐ 4 = Furniture<br>☐ 5 = Other (specify): ____________________

For peer review only

**SECTION G: Conclusion & Scheduling Next Interview**		
**SCRIPT:** Now, I have some final questions about your (the patient's) current health status.		
89	Compared to how you felt before this illness, how is your overall physical wellness today?	☐ 1 = Worse<br>☐ 2 = The same<br>☐ 3 = Better
90	Do you feel that you (or the patient) has fully recovered from cholera?	☐ 1 = Yes → **continue to SECTION H to Conclude Interview**<br>☐ 2 = No → **continue to below script**<br>☐ 3 = Not sure → **continue to below script**
**SCRIPT:** It sounds like you are still feeling sick because of cholera. So, we would like to follow-up in approximately 7 days with another interview to check on your health. For the next interview, you will not need to answer the questions again about your living arrangements, household belongings, and water, sanitation, and hygiene practices.		

For peer review only - http://bmjopen.bmj.com/site/about/guidelines.xhtml

Cholera Surveillance In Malawi (CSIMA) Program
NSANJE DISTRICT USE ONLY

Form S.I.#:__________________ **Study label**

(To be filled by Data Management) N☐☐-☐☐☐☐

During the second interview, we will only ask you questions about any health services you have tried to seek between today and the next interview. The survey will be almost exactly like this one. If you complete that second survey, we will give you a bar of soap for your time and participation.

What is a convenient time for you? We will attempt to contact you at least 1 day in advance to remind you of the interview.

91	Date for second interview	**(DD/MM/YYYY):** ☐☐/☐☐/☐☐☐☐<br>**(HH:MM, 12-hr format):** ☐☐:☐☐ **AM** ☐ **PM** ☐

SCRIPT: At this second interview, I will ask you again to think about any places you have gone to seek additional treatment or care for this episode of cholera.

To help you remember these visits, here is a diary card where you can record basic information about the visit. If you are unable to fill out the card, please let me know. You will still receive a bar of soap at the next interview even if you do not fill out this diary card.

Interviewer: *Show the diary card to the patient and instruct the patient (or someone who can fill the card out on behalf of the patient) on how to fill out the card. Please ensure that they have a pen to fill it out. If they do not, please give them a pen. If it is afternoon when you are finishing the interview, you may want to ask if the patient has visited any place for treatment or care today. If the patient says "**yes**," you may as an example walk the patient (or person filling out card) through the card to begin to fill out today.*

92. Was the patient (or household member) given the diary card to fill out?

☐ Yes ☐ No (specify reason): ______________________________

Section H: Incentive & Thanking Patient

SCRIPT: Thank you very much for taking the time to answer my questions. That concludes our interview. To thank you for your time, here is a bucket that you may use as you like. We hope that it may be helpful for your household.

Section I: For SURVEY FIELD WORKER

I1. Interviewer Name: ______________________________

Interviewer Signature: ______________________________

I2. Date of Interview Completion (DD/MM/YYYY): ☐☐/☐☐/☐☐☐☐

I3. Please indicate any additional interview details:

☐ Interview completed in full ☐ Patient declined interview

☐ Patient not home ☐ Could not verify patient identity

☐ Patient asked to reschedule (fill out below) ☐ Other: ______________________

IVI_CSIMA_ v4.1, 24-April-2017

For peer review only - http://bmjopen.bmj.com/site/about/guidelines.xhtml

Cholera Surveillance In Malawi (CSIMA) Program

NSANJE DISTRICT USE ONLY

Form S.I.#:__________________

(To be filled by Data Management)

Study label

N☐☐-☐☐☐☐

***** If a second interview has been schedule, please be sure to transfer and record the scheduled interview date from Question #91 to the D-2 Cost-of-Illness Survey form. *****

Section J: Rescheduling Interview

***** If the interview must be rescheduled for any other reason (e.g., person not home, person wants to continue interview at a later date, person does not want to be interviewed today), please also enter a date below. *****

J1. Date of Rescheduled Interview (DD/MM/YYYY): ☐☐/☐☐/☐☐☐☐

J2. ***Interviewer:*** *Please use this section to write any important details about the interview and/or follow-up interviews for this patient that you feel would be important to remember.*

____

____

____

For peer review only

For peer review only - http://bmjopen.bmj.com/site/about/guidelines.xhtml
